# Enzyme catalysed Pictet-Spengler formation of chiral 1,1'-disubstituted- and spiro-tetrahydroisoquinolines

**DOI:** 10.1038/ncomms14883

**Published:** 2017-04-03

**Authors:** Benjamin R. Lichman, Jianxiong Zhao, Helen C. Hailes, John M. Ward

**Affiliations:** 1 Department of Biochemical Engineering, University College London, Gower Street, London WC1E 6BT, UK; 2 Department of Chemistry, University College London, Christopher Ingold Building, 20 Gordon Street, London, WC1H 0AJ, UK

## Abstract

The Pictet–Spengler reaction (PSR) involves the condensation and ring closure between a β-arylethylamine and a carbonyl compound. The combination of dopamine and ketones in a PSR leads to the formation of 1,1′-disubstituted tetrahydroisoquinolines (THIQs), structures that are challenging to synthesize and yet are present in a number of bioactive natural products and synthetic pharmaceuticals. Here we have discovered that norcoclaurine synthase from *Thalictrum flavum* (*Tf*NCS) can catalyse the PSR between dopamine and unactivated ketones, thus facilitating the facile biocatalytic generation of 1,1′-disubstituted THIQs. Variants of *Tf*NCS showing improved conversions have been identified and used to synthesize novel chiral 1,1′-disubstituted and spiro-THIQs. Enzyme catalysed PSRs with unactivated ketones are unprecedented, and, furthermore, there are no equivalent stereoselective chemical methods for these transformations. This discovery advances the utility of enzymes for the generation of diverse THIQs *in vitro* and *in vivo*.

## Abstract (web summary)

The Pictet-Spengler condensation of β-arylethylamine and carbonyl compounds is an important step in the synthesis of bioactive alkaloids. Here, the authors report a Pictet-Spengler reaction between dopamine and unactivated ketones catalysed by norcoclaurine synthase and its engineered variants.

The Pictet–Spengler reaction (PSR) involves the condensation and ring closure between a β-arylethylamine and a carbonyl compound, typically an aldehyde^1^. Ketones can be employed in PSRs to yield 1,1′-disubstituted tetrahydroisoquinolines (THIQs), which feature a nitrogen substituted quaternary center (α-tertiary amine). Such structures are the basis of several natural products and pharmaceutical compounds including the *Erythrina* alkaloids and FR115427 (Fig. 1). However, compounds containing α-tertiary amines are typically challenging to synthesise^2^, and the use of unactivated ketones in the Pictet–Spengler reaction has been limited by their low reactivity and steric bulk.

General chemical strategies for PSRs with unactivated ketones can involve Brønsted acid catalysis^3^,^4^, Lewis acid catalysis^5^,^6^ or use of fluorinated solvents^7^. However, to our knowledge, there are no general methods for stereoselective chemical PSRs with ketones. Importantly, efficient routes to chiral 1,1′-disubstituted THIQs remain a significant unsolved challenge within synthetic chemistry^8^.

Several enzymes catalysing stereoselective PSRs have been characterized from plant benzylisoquinoline alkaloid biosynthesis^9^,^10^,^11^,^12^ and indole alkaloid biosynthesis^13^,^14^. Pictet–Spenglerases have also been identified in bacterial secondary metabolism^15^,^16^,^17^. The plant enzymes have a central role in engineered microbial systems for the production of alkaloids^18^,^19^,^20^,^21^,^22^. Furthermore, the wide substrate promiscuity of these enzymes has led to their use in biocatalytic syntheses, but the carbonyl substrates employed have always been aldehydes^23^,^24^,^25^,^26^,^27^,^28^,^29^,^30^,^31^,^32^. The only example of a Pictet–Spenglerase accepting non-aldehyde carbonyl substrates was the report of a norcoclaurine synthase (NCS) from *Coptis japonica* (*Cj*NCS2, also known as *Cj*PR10A) turning over 4-hydroxyphenylpyruvate and pyruvic acid, activated α-keto acids^10^. There have, however, been no previous reports of enzymatic Pictet–Spengler catalysis with unactivated ketones.

Here we present the first examples of enzyme catalysed PSRs with unactivated ketones. We have discovered that *Thalictrum flavum* NCS (*Tf*NCS), the Pictet–Spenglerase involved in benzylisoquinoline alkaloid biosynthesis, can accept a wide range of unactivated ketones *in vitro* in high yields. We have also identified *Tf*NCS variants with improved ketone tolerance and used these enzymes for the facile biocatalytic formation of novel chiral 1,1′-disubstituted- and spiro-THIQs.

## Results

### Observation of ketone acceptance

Encouraged by the wide aldehyde substrate scope demonstrated by NCSs^27^,^28^,^29^,^30^,^31^, we investigated their ability to accept ketones as substrates (Fig. 2). First, we tested WT NCSs Δ29*Tf*NCS^9^,^31^,^33^ (29 amino-acid N-terminal truncation) and *Cj*NCS2 (refs 10, 28) for the conversion of dopamine **1** and the ketone 4-hydroxyphenylacetone **2** into a 1,1′-disubstituted THIQ (Fig. 3a). The ketone **2** was selected as it is structurally similar to the natural NCS aldehyde substrate 4-hydroxyphenylacetaldehyde (4-HPAA)^34^. The NCS enzymes were recombinantly expressed in *Escherichia coli* and examined as crude lysates (Supplementary Fig. 1). The lysates were incubated with dopamine **1** and ketone **2,** and reactions were then analysed by HPLC-MS. For reactions containing Δ29*Tf*NCS, a new product with an *m/z* of 286.1 was observed, corresponding to the 1,1′-disubstituted THIQ product **3** (Fig. 3b). This product **3** was not observed in reactions with *Cj*NCS2, phosphate buffer or the empty vector (EV) control. The formation of **3** by Δ29*Tf*NCS was found to be strongly influenced by the co-solvent employed, with reactions containing DMSO producing larger quantities (more than fivefold) of the product **3** than those with acetonitrile (Supplementary Fig. 2); this was perhaps a result of enzyme stability effects. The observation that Δ29*Tf*NCS could accept ketone 4-hydroxyphenylacetone **2** provided a starting point for a wider examination of this novel enzyme activity.

### *Tf*NCS variant screen

In order to identify the scope of the NCS ketone PSR, variants of Δ29*Tf*NCS were screened with dopamine and a range of ketones (Fig. 4; Supplementary Figs 3 and 4). Enzyme variants tested were rationally chosen based on the NCS dopamine-first mechanism and prior docking studies with the natural aldehyde substrate 4-HPAA^28^,^31^. Previously examined amino-acid residues that were found to play a minor role in the enzyme mechanism were targeted (Y108F and D141E/N). Prior docking studies had also identified Met-97 as occupying a position proximal to the iminium intermediate; it was thought that substitutions of this residue (M97F/L/V) may increase the space available in the active site for bulkier ketone substrates. The active site entrance loop region (amino acids 76–80) was also examined as both experimental and docking studies had suggested this region influences the carbonyl substrate acceptance^31^. Variants with altered hydrophobic interactions in this loop region were therefore investigated (L76A/V, A79F/I, F80L).

Along with ketone **2** (Fig. 3), Δ29*Tf*NCS activity was observed with the related methyl ketone phenylacetone **4** (Fig. 4). A number of cyclohexanones also appeared to be turned over by the enzymes, which was remarkable given the structural differences between these compounds and the natural substrate. Enzyme products were observed for cyclohexanone (**6**), and 4-methyl- (**7**), 4-*tert*-butyl- (**8**) and 4-phenyl- (**9**) substituted cyclohexanones (Fig. 4, Supplementary Fig. 3). The single isomer ketone (3R)-methylcyclohexanone ((*R*)-**10**) was also converted into a new product by the enzymes.

No products were identified for several other ketones when incubated with Δ29*Tf*NCS variants and dopamine (Supplementary Fig. 4). In most cases, the lack of activity can be explained by steric effects. Ethyl ketones (3-pentanone) or very bulky methyl ketones (adamantylmethylketone) did not appear to be accepted. Although the Δ29*Tf*NCS variants accepted 3- and 4-substituted cyclohexanones, substitution on the 2-position was not accepted, nor was tri-substituted 3,3,5-trimethylcyclohexanone. The less reactive conjugated ketones were not turned over (acetophenone, cyclohexenone), whilst other ketones (propiophenone, isophorone) were not accepted presumably due to a combination of steric effects and lower reactivity.

All enzyme variants examined with ketones **2**, **4** and **6**-**10** showed conversions (Fig. 4b). The variants M97L, M97V, D141E and D141N showed consistently lower conversions than WT, perhaps due to the amino-acid substitutions adversely affecting the enzyme mechanism or structure in a general manner. For example, the Met-97 side-chain points into the active site, positioned near to the iminium moiety of the reaction intermediate. The shortening of this side chain to L or V may increase the conformational freedom of the iminium intermediate, thereby reducing catalytic efficiency. Also, the Asp-141 carboxylate is likely to interact electrostatically with the amino group of the dopamine substrate; variation of this amino-acid side chain to glutamate (with an extra methylene group) caused a reduction in conversions, whilst removal of the charge (D141N) had a more severe effect. Similar effects were shown previously with the substrates 4-HPAA and hexanal^31^. The variants L76V, F80L, M97F and Y108F generally appeared inferior to wild type (WT) with the methyl ketones (**2** and **4**), but for specific cyclohexanones (**6**-**10**) slightly exceeded WT conversions. The most notable amino-acid substitutions were those on the Ala-79 residue: A79I gave conversions twice that of WT for the methyl ketones (**2** and **4**), whilst A79F showed increased conversions for all the cyclohexanone substrates (**6**-**10**). Overall, the mutant screening identified the range of ketones accepted by Δ29*Tf*NCS along with variants showing improved conversions. Enzyme reactions were then scaled-up into biocatalytic syntheses both in order to fully characterize the enzyme products and to demonstrate the synthetic potential of this system.

### Biocatalytic formation of chiral 1,1′-disubstituted THIQs

Novel chiral 1,1′-disubstituted- THIQs were obtained from dopamine and methyl ketones via preparative scale (50 μmole) *in vitro* biotransformations. The ketones employed were phenylacetone **4** and 4-methoxyphenylacetone **5**, and the reactions were catalysed by the Δ29*Tf*NCS variant A79I (Fig. 5a). Conversion yields, based on the depletion of dopamine, were high: 91% and 74% for **4** and **5**, respectively. The THIQs were purified in good yields as hydrochloride salts using an extraction method which did not involve chromatographic separation (method adapted from Maresh *et al*.^29^). Isolated yields for the pure compounds **11** and **12** were 87% and 69%, respectively.

Methyl ketone-derived THIQ products **11** and **12** contained a chiral α-tertiary amine moiety and optical rotations were recorded for both compounds indicating a degree of enantiopurity (Fig. 5a). The stereoselectivity of the reactions was investigated further by determining the enantiomeric excess (e.e.) of (*S*)-**11**. In order to achieve this, *rac*-**11** was required as a racemic standard, and was obtained via a three step chemical synthesis (Fig. 5b; synthetic procedure adapted from Horiguchi *et al*.^5^). The synthesis was completed with an overall yield of 11%; this contrasts with the high yielding one-step enzymatic synthesis of **11**. Comparison of *rac*-**11** and (*S*)-**11** by chiral HPLC revealed that (*S*)-**11** was formed in 95% e.e. (Supplementary Fig. 5). On the basis of the previously observed selectivity of the enzyme with phenylacetaldehydes (95% e.e., *S*-isomer)^28^, *S*-stereochemistry was assigned to the enzymatic products (*S*)-**11** and (*S*)-**12**. This e.e. data demonstrated that NCS can perform stereoselective Pictet–Spengler catalysis with ketones: we know of no chemical or enzymatic catalysts that have been shown to be capable of such an activity.

### Biocatalytic formation of spiro-THIQs

Novel spiro-THIQs (**13**–**17**) were obtained in a similar manner to that described above for the chiral 1,1′-disubstituted THIQs, though the enzyme variant A79F was used instead of A79I (Fig. 6). Conversion yields, based on dopamine depletion, were generally excellent (75–99%), though isolated yields were significantly lower (27–58%). The syntheses involving methyl ketones (**11** and **12**) demonstrated superior purification efficiency compared to those with cyclohexanones (**13**–**17**). This difference is likely to have been due to a variation in the extraction efficiencies and material lost in washing procedures, since no alternative reaction products were observed to account for the consumption of dopamine.

The cyclohexanone-derived compounds isolated (**13**–**17**) all possessed a spiro-fused ring. Products generated from the 4-substituted cyclohexanones (**14**–**16**) all had a fixed stereocenter in the C-4 position, with the substituent exclusively in an equatorial orientation as determined by 2D NOESY NMR spectroscopy (Fig. 6a). The product derived from the chiral ketone (3*R*)-methylcyclohexanone (*R*)-**10** was exclusively the (1*R*,3*R*)-**17** enantiomer, with an equatorial C3-methyl substituent (Fig. 6b). This was also verified by 2D NOESY NMR spectroscopy, and is the first reported (1*R*)-THIQ to be formed by NCS. Notably, for all the 3- and 4-substituted cyclohexanones examined it appeared that the sole product was that with the substituent in the more stable and less sterically hindered equatorial conformation.

### Active site entrance loop

The active site entrance loop (residues 76–80) appeared to influence the enzyme tolerance for ketone substrates. Previously, an amino-acid substitution on this loop (L76A) was shown to modulate the aldehyde substrate tolerance^31^. In the screens using cell lysates here particular variants seemed to show greater product formation than WT (Fig. 4). To investigate these further, assays were conducted using known concentrations of purified enzymes (Supplementary Fig. 1). These assays demonstrated that the amino-acid substitutions A79I and A79F improved conversions for phenylacetone and cyclohexanone respectively (Supplementary Fig. 6).

In this study, the enzyme *Cj*NCS2 showed poor tolerance towards ketone substrates. No activity was observed with 4-hydroxyphenylacetone (Fig. 3b), and with cyclohexanone purified *Cj*NCS2 showed only a 6% conversion relative to WT Δ29*Tf*NCS (Supplementary Fig. 6b). A major difference between *Cj*NCS2 and *Tf*NCS is at the aforementioned active site entrance loop. *Cj*NCS2 has an extra amino acid in this region, which is likely to affect its structure and interactions with the reaction intermediates (*Tf*NCS: 76-LPGAF-80, *Cj*NCS2: 71-LP**A**GIF-76). On the basis of both the improved Ala-79 variant conversions and the poor *Cj*NCS2 conversions, it is clear that the loop encompassing residues 76–80 plays a key role in the carbonyl substrate tolerance of NCS.

### Computational docking

In order to probe the molecular basis of the NCS ketone activities, the effects of the amino-acid substitutions and the stereochemical outcomes of the reactions, a computational docking study was conducted. The iminium intermediates of the ketone PSRs were docked into the active site of the *Tf*NCS crystal structure^35^. The study revealed predicted binding modes that corresponded to the NCS dopamine-first enzyme mechanism, in which dopamine is buried in the active site whilst the ketone R-group is oriented into the bulk solvent at the active site entrance (Fig. 7; Supplementary Figs 7 and 8; Supplementary Table 1)^28^,^31^.

The mechanistically relevant binding modes of cyclohexanone-dopamine iminium intermediates showed a nearly identical arrangement to the natural reaction intermediate (Fig. 7a,b). Crucially, the iminium nitrogen occupied a position between Tyr-108 and Glu-110 and the catechol hydroxyl groups were adjacent to Lys-122. The ketone-derived cyclohexane ring portion was positioned near to Met-97 and residues on the active site entrance loop (76–80). The width of the active site tunnel matched exactly that of the cyclohexanone α-carbons. Certain substrates that were not accepted by the enzyme in assays, such as 2-substituted cyclohexanones or ethyl ketones, are more sterically bulky at these α-carbons and this may have inhibited binding. In contrast there appeared to be limited restriction on the equatorial 4-subsitutent of the cyclohexanone intermediates (**14**–**16**), which were oriented outside the active site in the docking binding modes (Fig. 7b; Supplementary Fig. 8). This can account for the diversity and size of 4-substituents accepted by the enzyme.

The results of docking methyl ketone intermediates into the NCS active site also provided mechanistically relevant binding modes in which the catechol hydroxyl was bound to Lys-122 (Fig. 7c,d). However, the cavity between Tyr-108 and Glu-110, which is typically occupied by the iminium nitrogen was instead occupied by the α-carbon methyl group. In the *trans*-iminium binding modes the iminium nitrogen proton pointed away from the mechanistically important residues Glu-110 and Asp-141 (Fig. 7c), whilst for the *cis*-iminium binding modes the nitrogen was positioned directly between Glu-110 and Asp-141 (Fig. 7d). It is not known whether the *cis-* or the *trans*-iminium is the enzymatic reaction intermediate. Furthermore, the docking results were ambiguous with respect to the stereochemical outcome of the reaction. Overall, although the docking of cyclohexanone intermediates fits extremely well with experimental observations, it appears that the methyl ketone docking results do not fully account for the observed enzyme activities.

## Discussion

Enzymatic Pictet–Spengler conversion of unactivated ketones is unprecedented. Reactions with these unactivated ketones are more energetically and sterically challenging than the previously described conversions of aldehydes or α-keto acids. It is therefore remarkable that conversions and yields are so high for such demanding substrates—indeed the enzyme seems to have catalytic ability beyond what is necessary for its natural reaction. Inorganic phosphate is capable of catalysing the aqueous formation of 1-substituted-THIQs from dopamine and aldehydes^36^, yet it cannot turn over ketones. The mechanism of phosphate catalysis and NCS enzymatic catalysis must therefore differ. Furthermore, the enzymatic ketone activity does not appear to have fully co-evolved with aldehyde reactivity, as demonstrated by poor *Cj*NCS2 conversion of ketones compared to *Tf*NCS, whereas both of these enzymes show good conversions of a range of aldehydes^27^,^28^,^29^,^30^,^31^. It is not known whether *in vivo* conditions in plant vacuoles, where NCS has been shown to reside^11^, are conducive to activity with ketones, or whether this activity is only revealed *in vitro*.

Amino-acid substitutions in the *Tf*NCS active site modified the ketone substrate tolerance of the enzyme. In particular, variants of Ala-79 demonstrated improved turnover of ketones. The active site entrance loop (amino acids 76–80) is an exposed hydrophobic region of the enzyme that interacts with ketone substrates at initial substrate binding and then as the reaction progresses. Modification of these hydrophobic interactions, and especially the increase of such interactions through the substitutions A79F and A79I, may improve the affinity of hydrophobic substrates with the enzyme. This hypothesis is supported by the docking study which predicted comparatively large affinities for the bulkier hydrophobic substrates 4-*tert*-butylcyclohexanone **8** and 4-phenylcyclohexanone **9** (Supplementary Table 1). The increase in side-chain steric interactions caused by the A79F or A79I substitutions may also aid the reaction progression by encouraging the reaction intermediate to adopt a conformation conducive to cyclisation. However, these hypotheses cannot yet account for the poor ketone activity of *Cj*NCS2. Our work has also demonstrated that NCS would be tractable for larger scale mutation and directed evolution studies; such studies are likely to increase the variety of substrates (aldehydes and ketones) capable of being accepted by the enzymes, and will also improve our understanding of the reaction mechanism.

**Figure 1:**
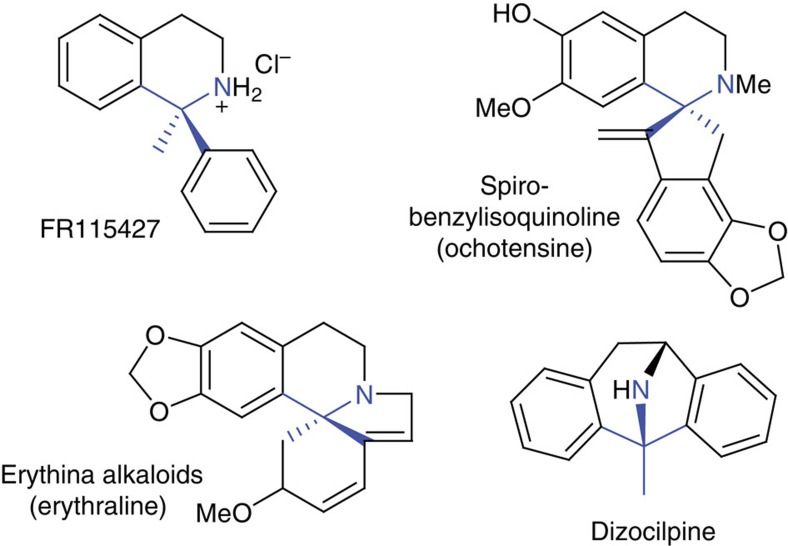
Pictet–Spengler formation of 1,1′-disubstituted tetrahydroisoquinolines. Examples of 1,1′-disubstituted tetrahydroisoquinolines (THIQs), with the α-tertiary amine moieties highlighted in blue. FR115427 and dizocilpine are synthetic anticonvulsants. The *Erythrina* and spiro-benzylisoquinoline alkaloids are subgroups of plant benzylisoquinoline alkaloids.

**Figure 2:**
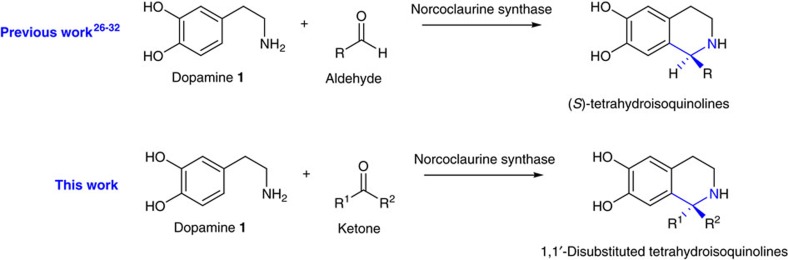
Norcoclaurine synthase activities. Previous work using NCSs demonstrated biocatalytic formation of 1-monosubstituted-THIQs from dopamine and aldehydes. In this work we demonstrate the biocatalytic route to 1,1′-disubstituted THIQs, from dopamine and ketones, via a Pictet–Spengler reaction, catalysed by NCS. The amines and substituted alpha-carbons is highlighted in blue.

**Figure 3:**
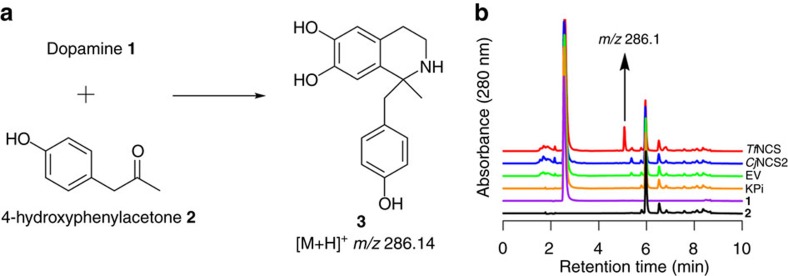
NCS activity with 4-hydroxyphenylacetone. (**a**) Proposed enzyme catalysed Pictet–Spengler condensation between dopamine **1** and an unactivated ketone (4-hydroxyphenylacetone **2**). (**b**) HPLC analysis of reactions between **1** and **2**. Substrates **1** and **2** were incubated together with phosphate buffer (KPi), control lysate with empty vector (EV), lysate containing Δ21*Cj*NCS2 or lysate containing Δ29*Tf*NCS. Formation of a new compound was observed in the *Tf*NCS sample. When analysed by MS, this compound had an *m/z* of 286.1, corresponding to THIQ **3**.

**Figure 4:**
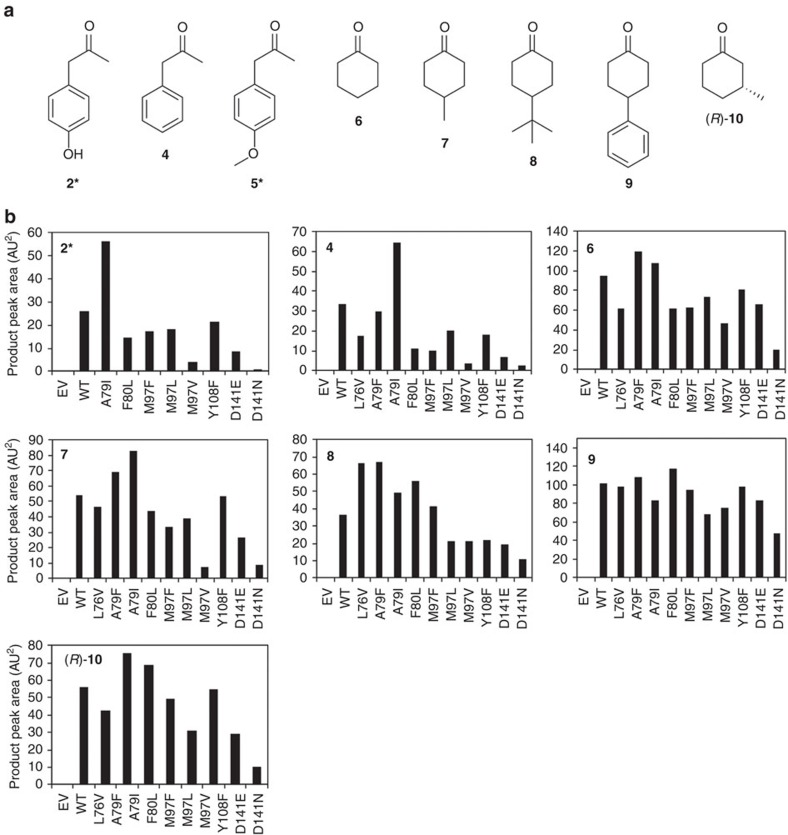
Ketones screened with Δ29*Tf*NCS variants. (**a**) Structures of ketones tested that showed conversion by Δ29*Tf*NCS variants. Ketones accepted: 4-hydroxyphenylacetone 2*, phenylacetone **4**, 4-methoxyphenylacetone 5*, cyclohexanone **6**, 4-methylcyclohexanone **7**, 4-*tert*-butylcyclohexanone **8**, 4-phenylcyclohexanone **9** and (3*R*)-methylcyclohexanone (*R*)-**10**. (**b**) Charts showing HPLC product peak area in reactions with dopamine, different ketones and different Δ29*Tf*NCS variants (EV, empty vector control). See Supplementary Fig. 3 for representative HPLC chromatograms and Supplementary Fig. 4 for structures of ketones not accepted by any Δ29*Tf*NCS variant. *Commercially available ketone **2** (4-hydroxyphenylacetone) was tested in the initial screen but degradation of the ketone starting material restricted its suitability for use in scale up reactions. Therefore, the more stable ketone **5** (4-methoxy-phenylacetone) replaced ketone **2** in the scale up biotransformations (Fig. 5a).

**Figure 5:**
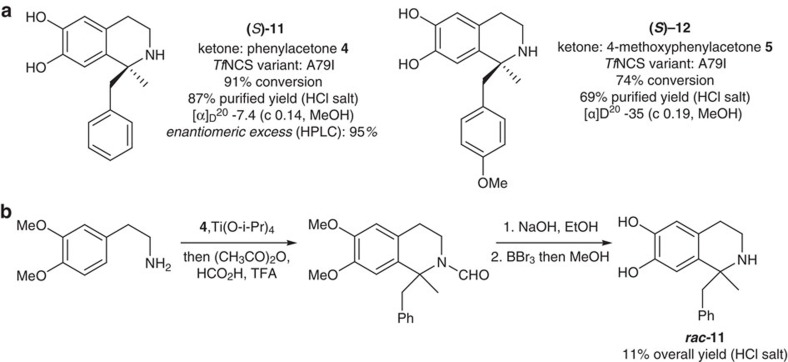
Biocatalytic formation of chiral 1,1′-disubstituted THIQs using Δ29*Tf*NCS variants. (**a**) Chiral 1,1′-disubstituted THIQs isolated from biotransformations. (**b**) Synthetic route towards *rac*-**11**. See Supplementary Fig. 5 for chiral HPLC analysis, Supplementary Methods for full synthetic procedures, Supplementary Figs 9 and 10 for achiral HPLC analysis, and Supplementary Figs 11-13 for NMR spectra.

**Figure 6:**
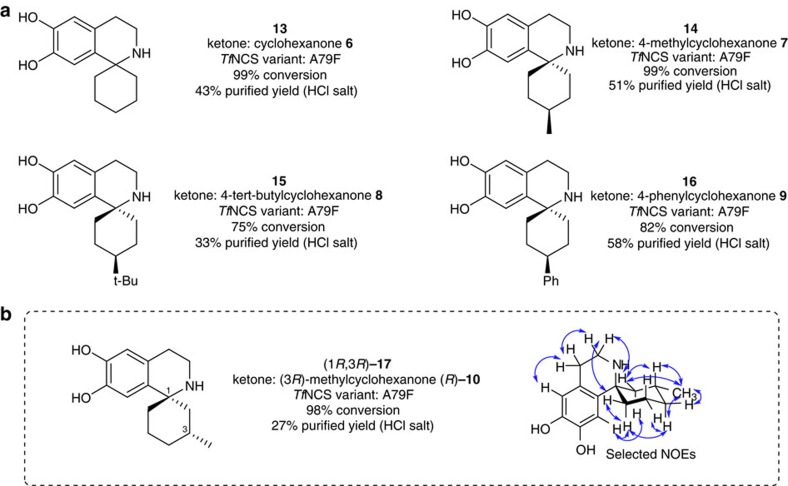
Biocatalytic formation of spiro-1,1′-disubstituted THIQs using Δ29*Tf*NCS variants. (**a**) Spiro-THIQ products isolated from biotransformations. All compounds feature a fixed stereocentre at the C-4 position. (**b**) Chiral (*R*)-Spiro-THIQ product (1*R*,3*R*)-**17** isolated from biotransformation. Important NOE (nuclear Overhauser effect) correlations are highlighted. See Supplementary Methods for full synthetic procedures, Supplementary Figs 9 and 10 for HPLC analysis and Supplementary Figs 14-18 for NMR spectra.

**Figure 7:**
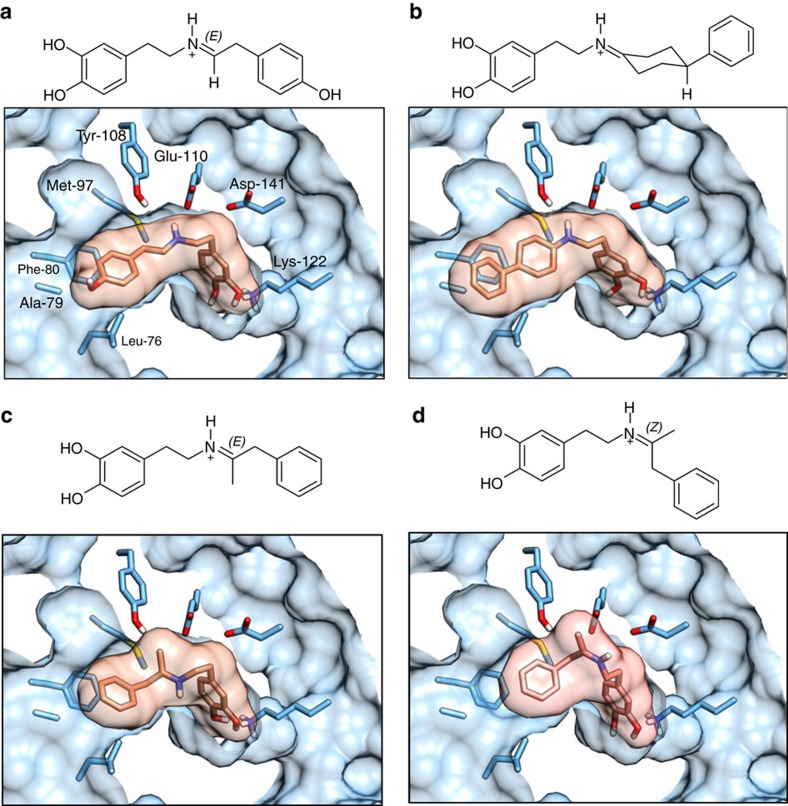
Reaction intermediate binding modes. Iminium reaction intermediates were docked in the *Tf*NCS active site using AutoDock Vina^42^. The structure of the docked iminium ligand is shown above a depiction of the mechanistically relevant binding modes. The side chains of mechanistically important amino acids or amino acids modified in this study are shown as sticks. (**a**) Intermediate derived from 4-HPAA, the natural aldehyde substrate. (**b**) Intermediate derived from 4-phenylcyclohexanone **9**, a representative example of the docking results of cyclohexanone-derived compounds. (**c**) Intermediate derived from phenylacetone **4** with *trans* (*E*) double bond. (**d**) Intermediate derived from phenylacetone **4** with *cis* (*Z*) double bond. Complete docking results can be found in Supplementary Figs 7 and 8 and Supplementary Table 1.

The activity with substituted cyclohexanones enables the biocatalytic formation of spiro-THIQs. Compounds with similar structures are present in plants as part of the *Erythrina* and spiro-BIA subfamilies e.g. the schelhammerans or homoerythrinane alkaloids (Fig. 1a)^37^,^38^. Although the biosyntheses of these compounds have not been fully elucidated, it is known that these structures are not formed directly via a PSR^39^,^40^. The results here demonstrate that an alternative enzymatic route to these compounds is feasible. Furthermore, the rigidity and three-dimensional nature of spirocycles makes them attractive moieties in drug discovery^41^, and a facile enzymatic approach to such structures will be of use in the future.

We have demonstrated the remarkable capacity of NCS to catalyse the formation of chiral 1,1′-disubstituted- and spiro-THIQs. This enzymatic catalytic activity of NCS with ketones rivals even the most advanced chemical THIQ Pictet–Spengler catalysts. Recently, there has been significant progress in the development of benzylisoquinoline alkaloid metabolic engineering^20^,^21^. NCS plays a vital role in these systems and, with this discovery, can now form the foundation of novel cascade systems incorporating ketones in biosynthetic pathways. Overall, this study has considerably expanded the repertoire of accessible THIQs, opening the door to a vast array of biocatalytically produced bioactive 1,1′-disubstituted THIQs.

## Methods

### Gene sequences

Codon-optimized genes encoding C-terminal His-tagged Δ29*Tf*NCS variants and C-terminal His-tagged codon Δ21*Cj*NCS2 were obtained in pJ411 and pD451 vectors respectively (DNA2.0; Menlo Park, CA, USA). Δ29*Tf*NCS Met-97 variants were prepared from WT Δ29*Tf*NCS using QuikChange Lightening mutagenesis kit (Agilent, Santa Clara, CA, USA) and mutagenesis primers (Forward: 5′-ATTCTGGACNTCACCTTTGTCCCGGGTGAATTCCCGCAC-3′, Reverse: 5′-CTACCGCCACAACCATGCTAAGACCTGNAGTGGAAACAG-3′, purchased from Eurofins, Luxembourg). Full-length *Cj*NCS2 used in purified enzyme assays and was obtained as previously reported^28^. The empty vector control used was a pET29a vector. All sequences were verified by Sanger sequencing provided by Source BioScience. *Tf*NCS and *Cj*NCS2 protein sequences can be found on UniprotKB with the identifiers Q67A25 and A2A1A1 respectively.

### Protein expression

Plasmids were transformed into *E. coli* BL21(DE3) cells by a standard heat-shock protocol. Transformants were inoculated into 20 ml of terrific broth (TB) medium (50 μg ml^−1^ kanamycin; Miller, Merck Millipore). Starter cultures were incubated 16 h at 37 °C, shaking at 250 rev min^−1^. Fresh TB media (with 50 μg ml^−1^ kanamycin) was inoculated with the overnight culture (4% v/v inoculant). The cultures were incubated for 2 h at 37 °C, followed by 1 h at 25 °C, shaking at 250 rev min^−1^. Expression was induced by the addition of isopropyl β-D-1-thiogalactopyranoside (IPTG, final concentration 500 μM). Cultures were incubated for 3 h (25 °C, 250 rev min^−1^) before collecting by centrifugation (10,000 *g*, 10 mins, 4 °C). The cell pellets were stored at −20 °C until further processing.

### Lysate preparation

Lysates used in initial substrate screens were prepared from 1 ml cultures. Cell pellets underwent five freeze–thaw cycles before resuspension in 100 μl 50 mM HEPES pH 7.5. The insoluble material was removed by centrifugation and the supernatant was used in reactions. For larger scale biotransformations or purified enzyme assays, cell pellets were resuspended in 50 mM HEPES pH 7.5 (10% of the original culture volume) and sonicated (10 s ON, 10 s OFF) until homogenous. The insoluble fraction was pelleted by centrifugation (10,000 *g*, 30 min, 4 °C). The supernatant was removed and filtered through a glass fibre prefilter and 0.2 μm cellulose acetate syringe filter (Sartorius Stedim Biotech, Göttingen, Germany). The clarified lysate was used directly in biotransformations or purified further.

### Enzyme purification

The filtered supernatant was passed through a 2 ml Ni-Sepharose column (HP resin, GE) previously equilibrated with binding buffer (0.1 M HEPES, 20 mM imidazole, 100 mM NaCl, pH 7.5; 10 ml), and the column was then washed with binding buffer (10 ml) followed by wash buffer (0.1 M HEPES, 40 mM imidazole, 100 mM NaCl, pH 7.5; 20 ml). The bound protein was eluted (0.1 M HEPES, 500 mM imidazole, 100 mM NaCl, pH 7.5; 5 ml). The eluent containing pure enzyme was buffer exchanged into assay buffer (50 mM HEPES pH 7.5), using a PD-10 column (GE). Protein purity was established by SDS–PAGE (Supplementary Fig. 1). Protein concentration was determined using absorbance at 280 nm. For storage, glycerol was added (10% v/v); the protein was flash-frozen in liquid nitrogen and stored at −80 °C.

### Chemical reagents

All reagents were obtained from commercial sources (Sigma-Aldrich, St. Louis, MO, USA) and used as received unless otherwise stated. 4-Hydroxyphenylacetone was provided by AldrichCPR and was only of sufficient purity to use in screens and not in the biotransformations. Dopamine hydrochloride was provided by Alfa Aesar.

### HPLC analysis

Achiral HPLC analysis was performed with a system consisting of a LC Packing FAMOS Autosampler, a Dionex P680 HPLC Pump, a Dionex TCC-100 Column oven and a Dionex UVD170U Ultraviolet detector. Separation conditions: HiChrom ACE C18-5 (150 × 4.6 mm) column, 1 ml min^−1^ gradient of H_2_O (0.1% v/v TFA) and acetonitrile 90:10 to 30:70 over 5 min, 30 °C, 280 nm absorbance detection and 20 μl injection volume. Chiral HPLC analysis was conducted on an Agilent Technologies 1260 Infinity machine with a ultraviolet detector, a Supelco Astec Chirobiotic T column (25 cm × 4.6 mm, 5 μm), flow rate 1 ml min^−1^, at 25 °C, eluent ethanol-ammonium acetate (15:85; 5 mM, pH 3.5) and detection at 230 nm.

### Chemical analytics

MS analysis was performed in the UCL Chemistry Mass Spectrometry Facility. Compounds were ionized using electrospray ionization and detected in positive mode on a Waters LCT Premier XE (**3**, **11**, **12**, **17**, **18**) or a Q-TOF Agilent 6510 (**14**, **15**, **16**). All compounds were identified by their [M+H]^+^ signal. NMR spectra were recorded at 298 K at 600 MHz using a Bruker Avance 600. Chemical shifts (in p.p.m.) are quoted relative to tetramethylsilane and referenced to residual protonated solvent. Coupling constants (*J*) were measured in Hertz (Hz) and multiplicities for ^1^H NMR coupling are shown as s (singlet), d (doublet), t (triplet) and m (multiplet). Two-dimensional COSY, HSQC, HMBC and NOESY spectra were used for compound identification.

### Compound screening

For initial detection of activity with 4-hydroxyphenylacetone, reactions contained: dopamine **1** 10 or 15 mM, 4-hydroxyphenylacetone **2** 10 or 15 mM, 10 or 15% v/v DMSO or MeCN co-solvent (when **2** was limiting or in excess, respectively), 50% v/v clarified cell lysate (for Δ29*Tf*NCS, Δ21*Cj*NCS and empty vector control). For phosphate control: 750 mM potassium phosphate pH 7 was present, and no cell lysate. Reactions were incubated for 3 h at 37 °C, quenched with HCl (final concentration 100 mM), centrifuged and analysed by HPLC. Fractions from the Δ29*Tf*NCS sample containing the new compound of interest **3** (retention time (RT)=5.1 min) were collected and analysed by MS. For subsequent screens with Δ29*Tf*NCS variants: 20% v/v lysate, 15 mM dopamine **1**, 10 mM ketone, 10% v/v DMSO, 37 °C, 6 h. Reactions were quenched with HCl (final concentration 100 mM), centrifuged and analysed by HPLC.

### Biotransformations

Reactions (5 ml) contained: 20% v/v lysate, 5 mM ascorbic acid, 10 mM ketone, 15 mM dopamine **1**, 10% v/v DMSO and 50 mM HEPES pH 7.5. The reaction was incubated for 6 h (37 °C, 250 rev min^−1^). The reactions were stored at −20 °C until further processing. For synthesis of **17**, conditions were modified: 50% v/v lysate, 5 mM ascorbic acid, 10 mM ketone, 20 mM dopamine, 10% v/v DMSO and 50 mM HEPES pH 7.5. The reaction was incubated for 16 h (37 °C, 250 rev per min^−1^). The reaction was centrifuged and filtered before extraction. Crude conversions were measured by depletion of dopamine using HPLC analysis. For HPLC traces of crude biotransformations, see Supplementary Fig. 9.

### Compound extraction

The reaction (5 ml) was diluted to 10 ml with water. The solution was extracted with ethyl acetate (3 × 20 ml). The fractions were combined and washed with brine (3 × 20 ml). The organic layer was dried with magnesium sulfate, filtered and the solvent removed *in vacuo*. The material was resuspended in 20 ml of a 1:1 mixture of dimethylcarbonate (DMC) and 0.1 M HCl. The lower organic layer was extracted with 0.1 M HCl (2 × 10 ml). The aqueous layers were combined and water was removed *in vacuo* (first by a rotary evaporator and the last few millilitres by freeze-drying) to yield the product as a solid hydrochloride salt. For compounds **14**, **15** and **16** dichloromethane was used in place of DMC.

### Complete chemical syntheses and analyses

Detailed synthetic methods, including the synthesis of *rac*-**11**, can be found in the Supplementary Methods. For HPLC and NMR analyses of purified compounds see Supplementary Figs 10–18.

### Purified enzyme assays

Assays with purified enzymes contained 15 mM dopamine **1**, 10 mM ketone, 5 mM ascorbate, 50 mM HEPES pH 7.5, 10% v/v DMSO, 20% v/v empty vector lysate, 0.25 mg ml^−1^ purified enzyme. Empty vector lysate was included to replicate reaction conditions in previous screens and biotransformations. Reactions were quenched with HCl (final concentration 100 mM) and analysed by HPLC. Each reaction was performed in duplicate. Product peak areas were normalized to molar enzyme concentrations and reported as a proportion of WT Δ29*Tf*NCS peak area for direct comparison.

### Computational docking

Reaction intermediates were energy optimized using MM2 energy minimization (ChemBio3D, CambridgeSoft). The receptor used was subunit A from the *Tf*NCS crystal structure 2VQ5 (residues 40–191, with ligands removed)^35^. Docking was performed using AutoDock Vina (exhaustiveness=10)^42^. Docking box parameters (x,y,z): center (22.27, 21.16,-27.51); size (23.56, 17.37, 21.78). The exception to the method was the iminium intermediate of **17**—no MM2 minimization was conducted and modified docking box parameters were used: center (22.15, 21.15, −27.94), size (16.61, 16.56, 13.43). For each docking calculation, the nine clusters with the lowest free energy were visualized and their structure analysed. The mechanistically relevant binding modes (corresponding to the dopamine-first mechanism^28^,^31^) were selected, and their ranking and predicted affinity was recorded (Supplementary Table 1). Figures of docking results were prepared using UCSF Chimera.

### Data availability

The data that support the findings of this study are available from the corresponding authors upon reasonable request.

## Additional information

**How to cite this article:** Lichman, B. R. *et al*. Enzyme catalysed Pictet-Spengler formation of chiral 1,1′-disubstituted- and spiro-tetrahydroisoquinolines. *Nat. Commun.* **8,** 14883 doi: 10.1038/ncomms14883 (2017).

**Publisher's note:** Springer Nature remains neutral with regard to jurisdictional claims in published maps and institutional affiliations.

## Supplementary Material

Supplementary Information Supplementary Figures, Supplementary Table, Supplementary Methods.
