# Supplementary material for: Enzyme catalysed Pictet-Spengler formation of chiral 1,1'-disubstituted- and spiro-tetrahydroisoquinolines
Source: Nat Commun. 2017 Apr 3;8:14883. doi: 10.1038/ncomms14883 (PMC5382262; doi:10.1038/ncomms14883)
Supplement: Supplementary Information — Supplementary Figures, Supplementary Table, Supplementary Methods. [file ncomms14883-s1.pdf]

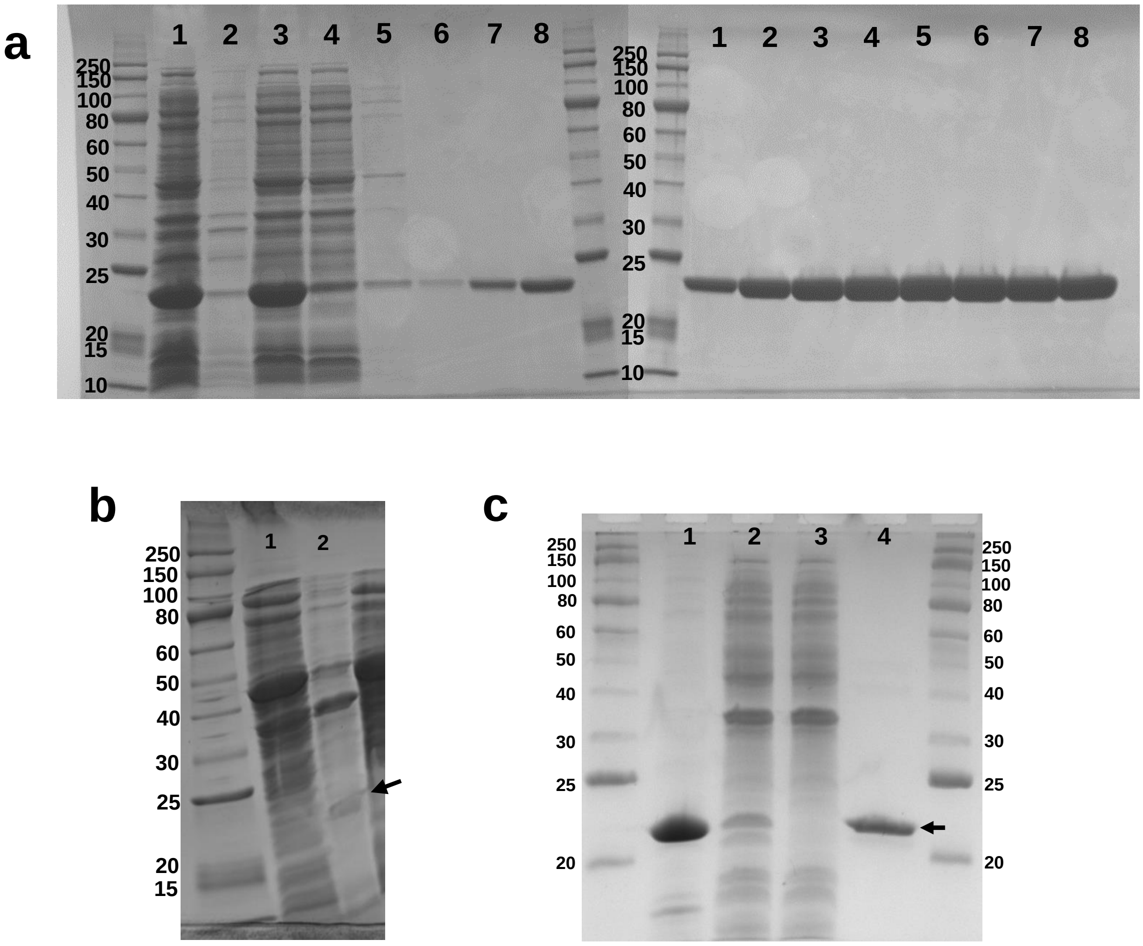

Supplementary Figure 1. SDS-PAGE analysis of norcoclaurine synthase (NCS) recombinant expression and purification. a. WT-Δ29*Tf*NCS expression and purification. LHS Lane 1: crude lysate; 2: pellet; 3: clarified lysate (supernatant after centrifugation, as used for biotransformations), 4: NiNTA flow-through; 5-6: 20 mM imidazole wash; 7-8: 40 mM imidazole wash; RHS Lane 1-8: 500 mM imidazole elution (purified enzyme). Δ29*Tf*NCS variants showed similar expression levels and behaved similarly during the purification procedures. **b.** Δ21*Cj*NCS2 expression. Lane 1: Crude lysate; 2: clarified lysate (supernatant after centrifugation, as used for biotransformations). Δ21*Cj*NCS2 is highlighted. **c.** Full length *Cj*NCS2 purification. Lane 1: pellet (insoluble fraction); 2: Lysate; 3: Flow-through; 4: Purified *Cj*NCS2, *Cj*NCS2 is highlighted.

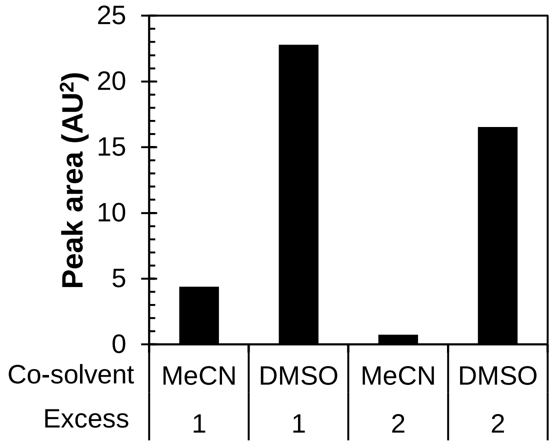

Supplementary Figure 2. The effect of solvent on conversions. Conditions affecting the production of the new product (corresponding to product **3**) in *Tf*NCS catalysed reactions between dopamine **1** and 4-hydroxyphenylacetone **2.** The peak area of proposed THIQ product **3** (*m/z* = 286.1, RT = 5.1 mins) with different amounts of substrate and co-solvents. Excess (**1** or **2**) refers to which starting material was used in excess. Conditions: 15 mM excess substrate, 10 mM limiting substrate, 50% v v^{-1} clarified lysate with *Tf*NCS, 10 or 15% v v^{-1} co-solvent (when **2** is limiting or in excess respectively). Reactions incubated for 3 hours at 37 °C.

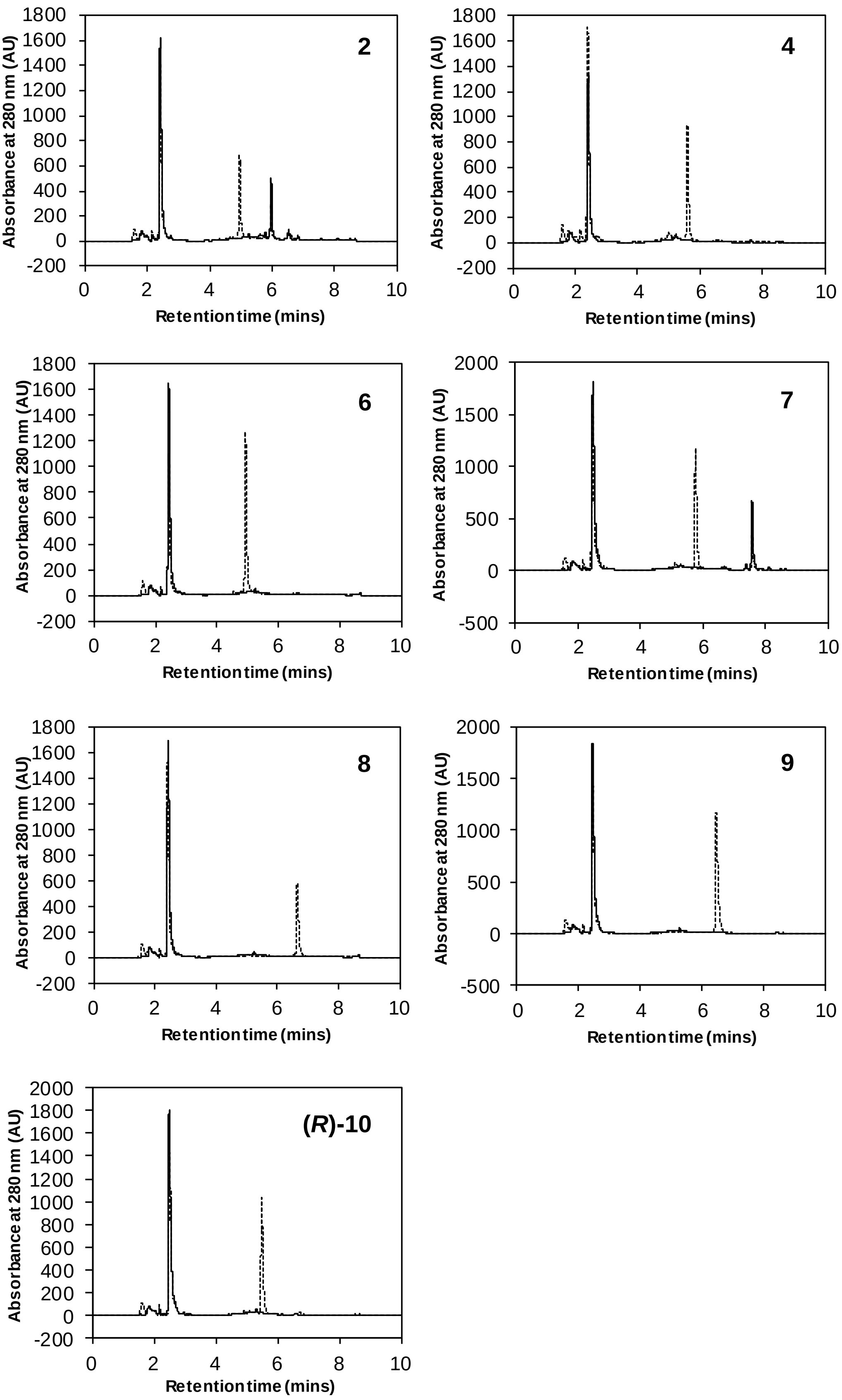

Supplementary Figure 3. Representative HPLC chromatograms from ketone screen. Solid line is empty vector control (EV), dashed line is best performing variant. Dopamine is peak at 2.5 min; new product peaks can be observed in samples containing active Δ29*Tf*NCS variants.

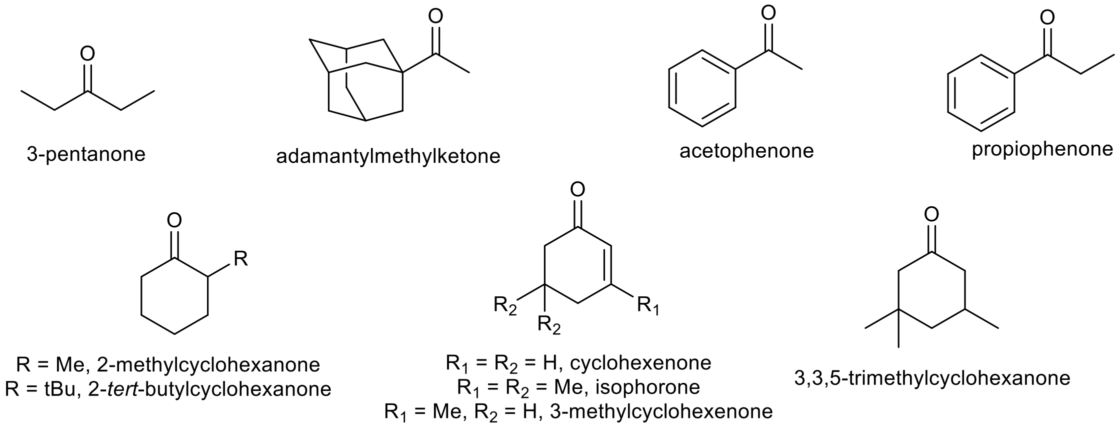

Supplementary Figure 4. Ketones for which no products were observed with Δ29*Tf*NCS variants.

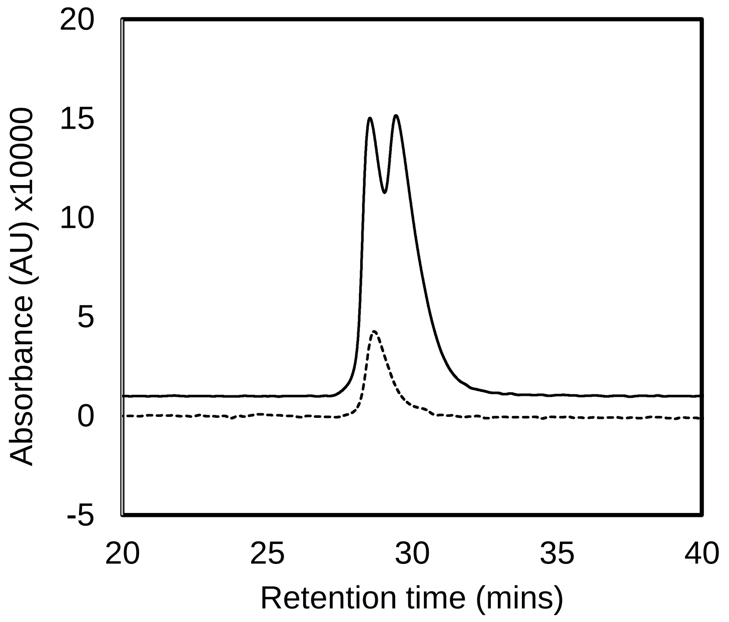

Supplementary Figure 5. Chiral-HPLC analysis of (*S*)-11 and *rac*-11. Dashed line shows (*S*)-**11** derived from enzyme catalyzed sample (Main Figure 5a). Solid line shows *rac*-11 derived from chemical synthesis (Main Figure 5b). The *enantiomeric excess* of (*S*)-**11** was determined to be 95%.

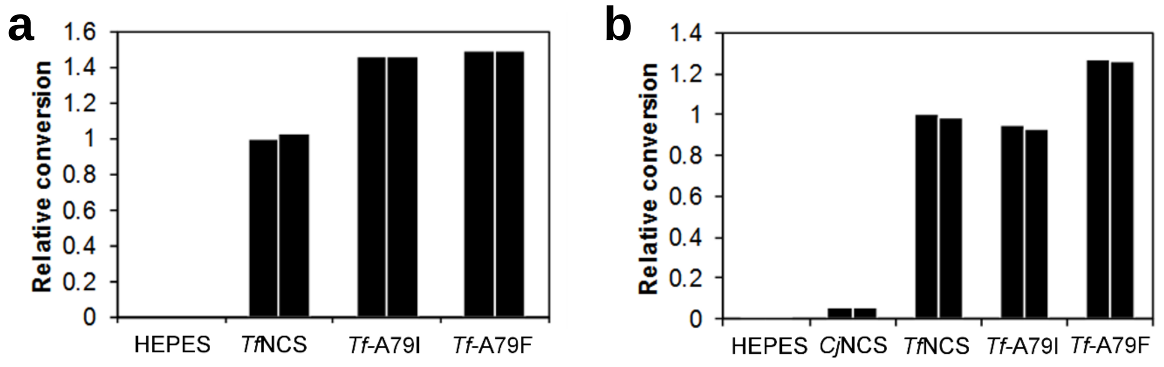

Supplementary Figure 6. Formation of 1,1'-disubstituted-THIQs with purified enzymes. a. Formation of THIQ **11** from dopamine **1** and phenylacetone **4** with purified enzymes. Assays were performed with two technical replicates and the conversions are normalized to wild-type *Tf*NCS. **b**. Formation of THIQ **13** from dopamine **1** and cyclohexanone **6** with purified enzymes. Assays were performed with two technical replicates and the conversions are normalized to wild-type *Tf*NCS. HEPES = buffer only negative control, *Tf*NCS = Δ29*Tf*NCS, *Cj*NCS = *Cj*NCS2, *Tf*-A79I/F = Δ29*Tf*NCS-A79I/F.

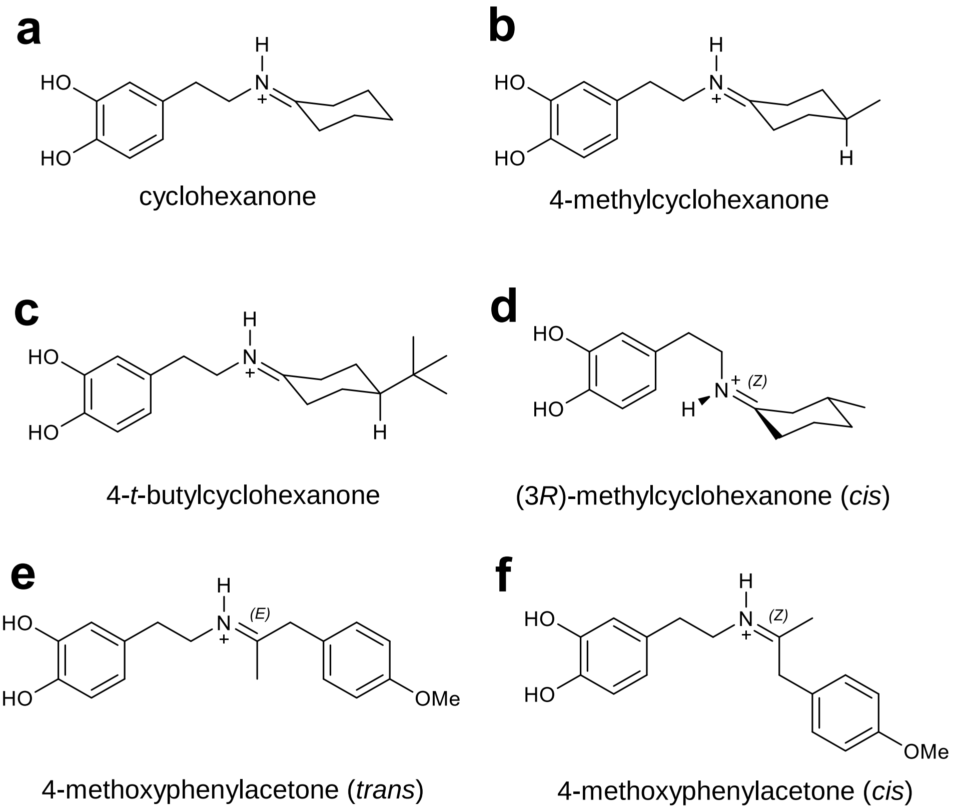

Supplementary Figure 7. Structures of iminium intermediates used in computational docking calculations. Mechanistically relevant binding modes of iminium intermediates derived from 4-HPAA, 4-phenylcyclohexanone and phenylacetone can be found in main **Figure 7**.

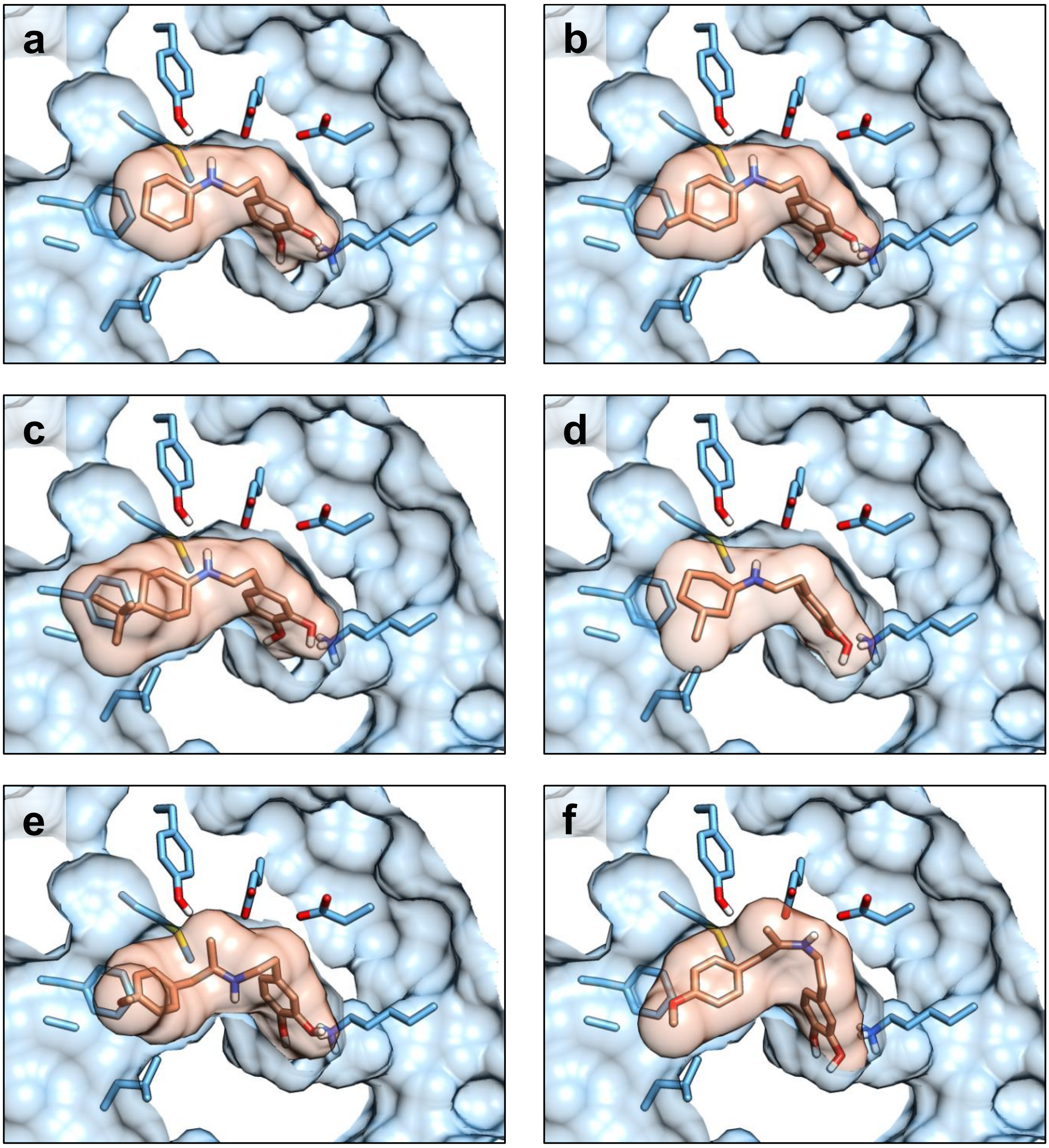

Supplementary Figure 8. **Mechanistically relevant docking modes of iminium intermediates.** See **Supplementary Table 1** below for energies and ranks of binding modes. See **Supplementary Figure 7** above for corresponding ligand structures.

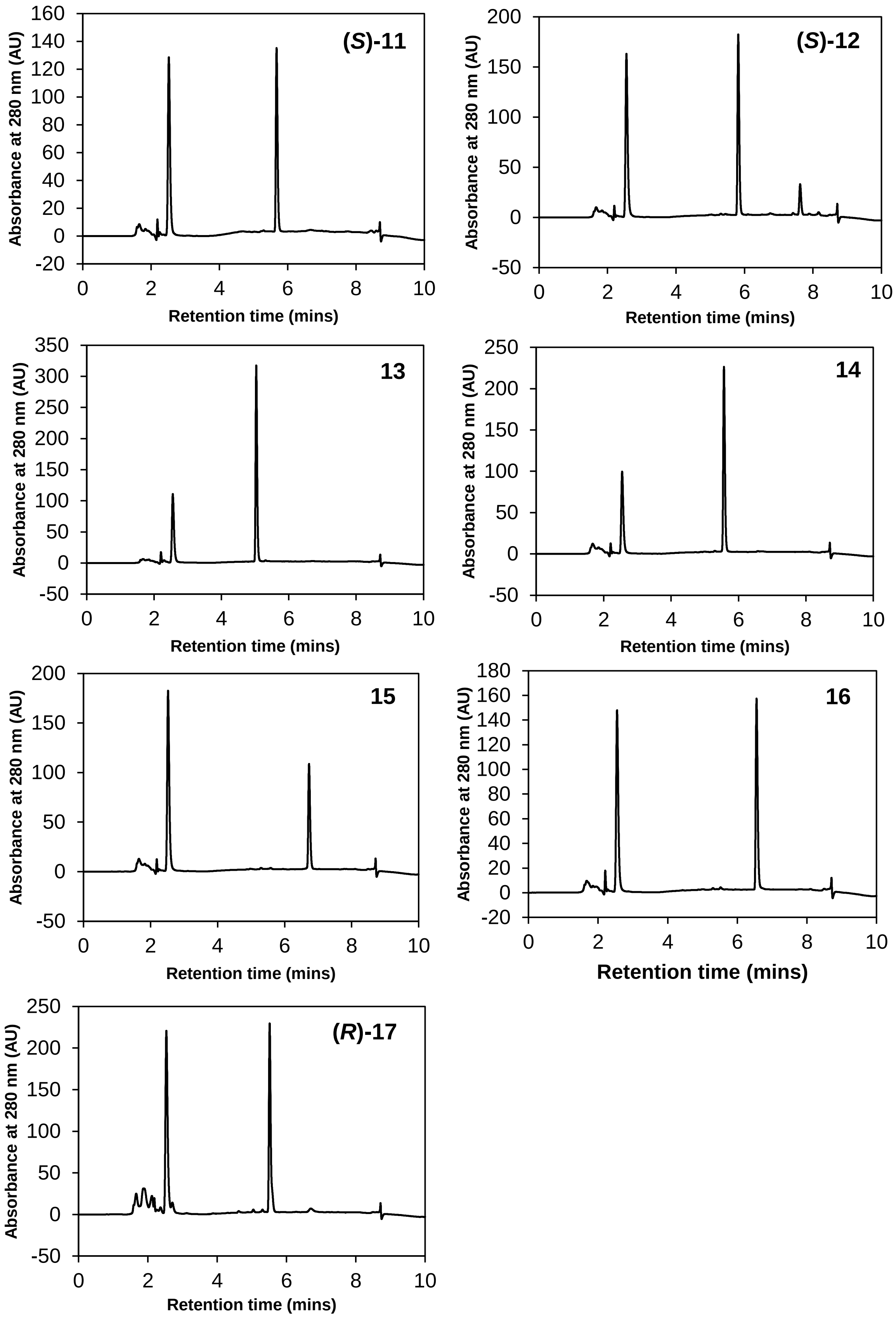

Supplementary Figure 9. HPLC chromatograms of biotransformations prior to extraction. Dopamine elutes at 2.5 mins. Tetrahydroisoquinolines typically elute between 5 and 7 mins.

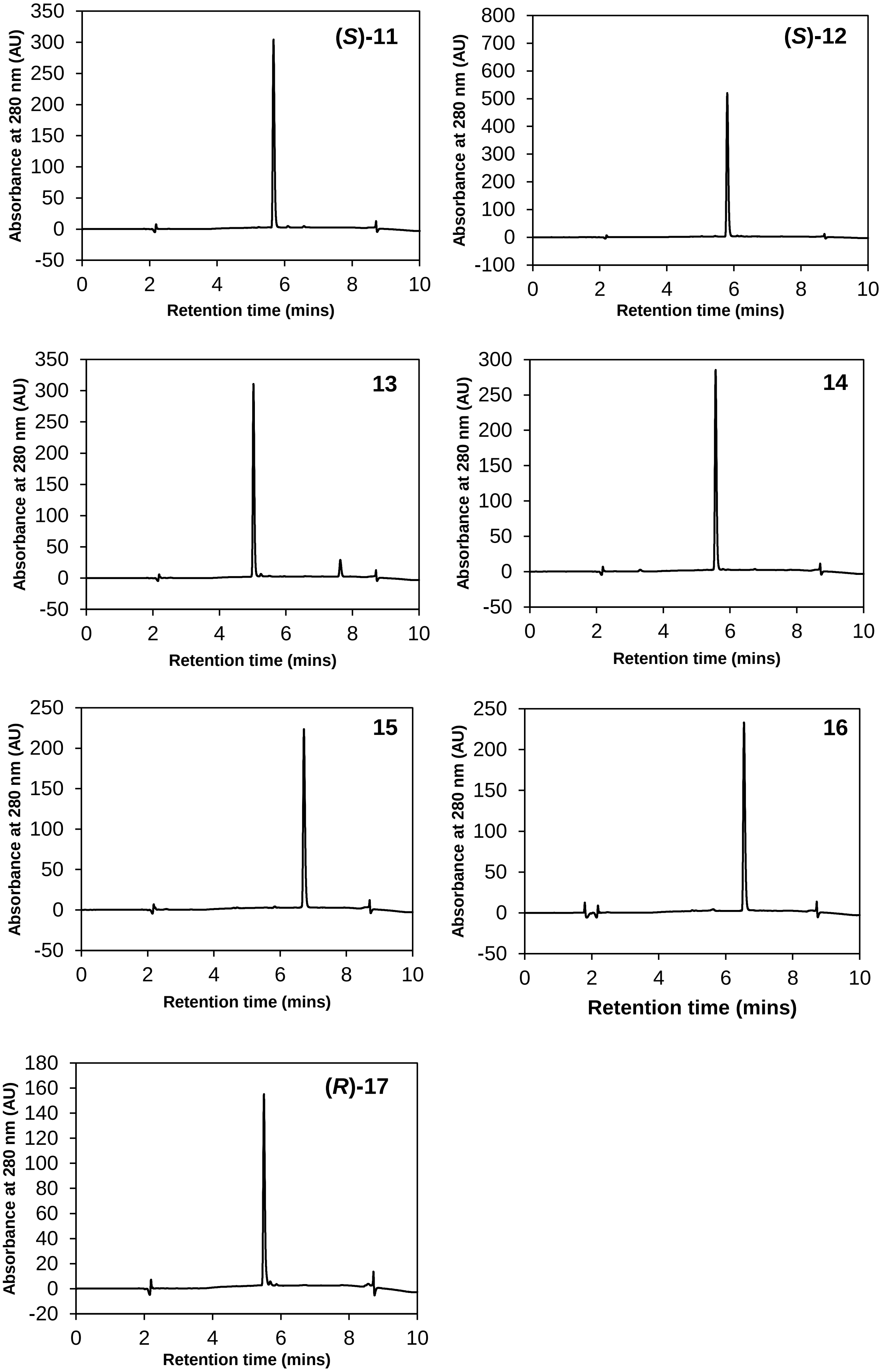

Supplementary Figure 10. HPLC chromatograms of purified tetrahydroisoquinolines.

a. ^{1}H

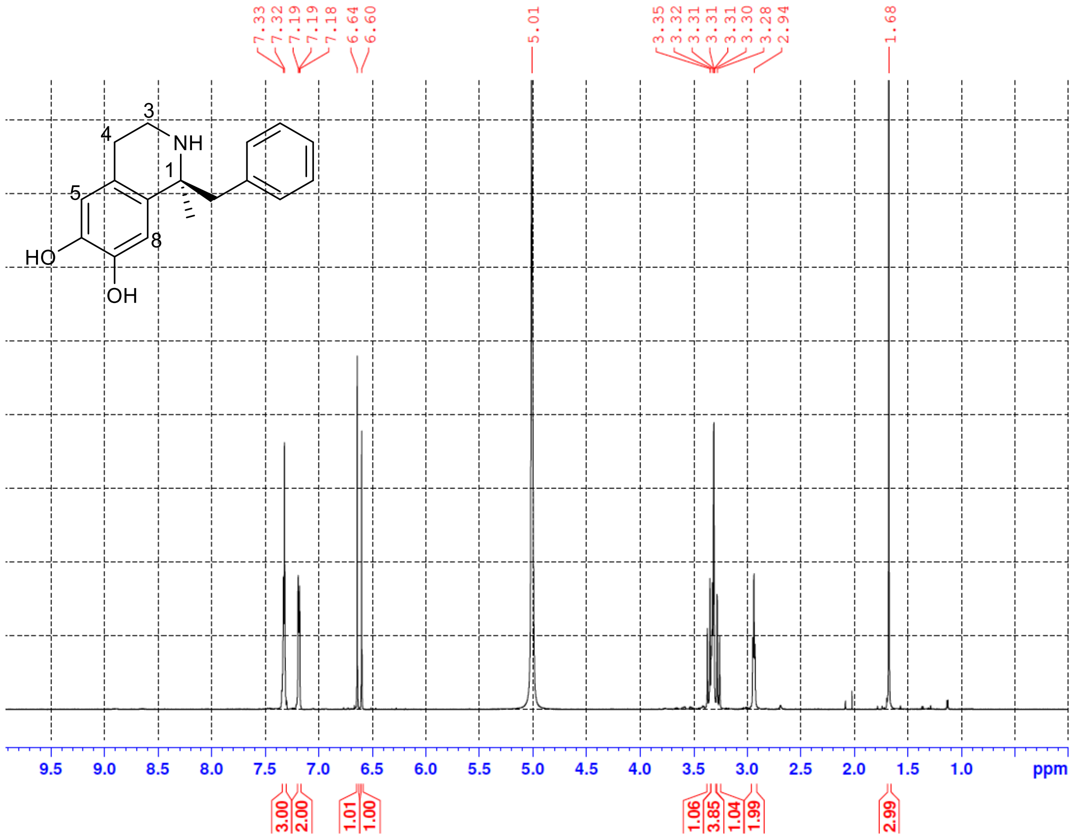

b. ^{13}C DEPT

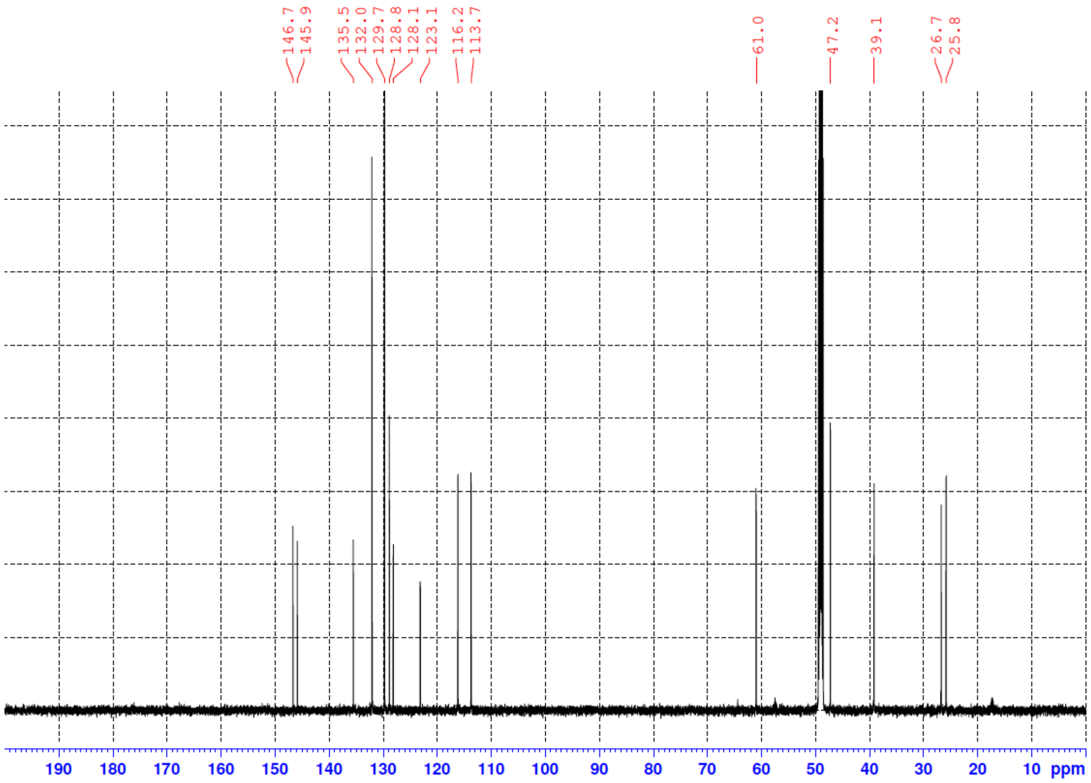

Supplementary Figure 11. NMR spectra of (*S*)-**11** (continued below).

c. COSY

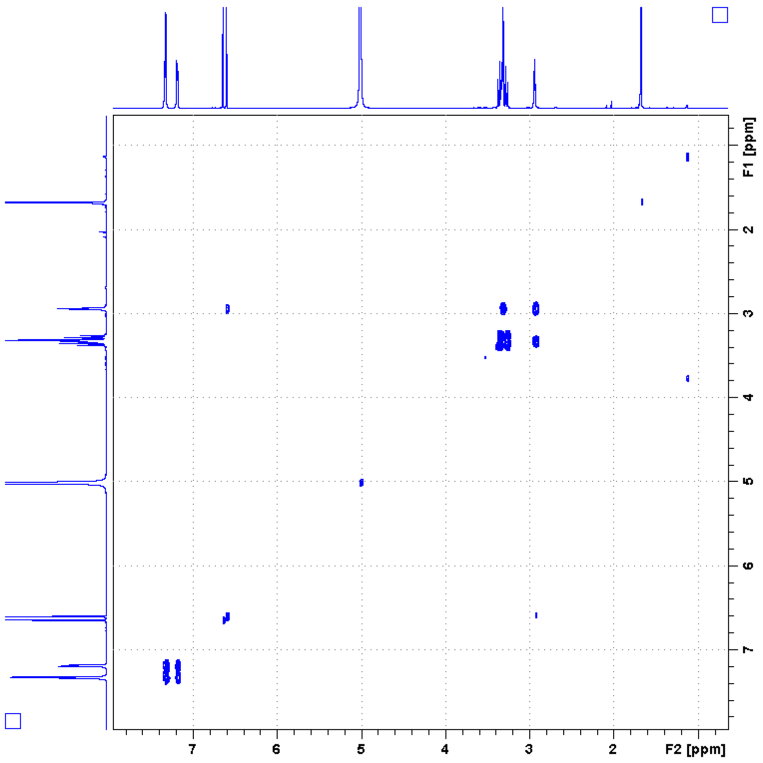

d. HSQC

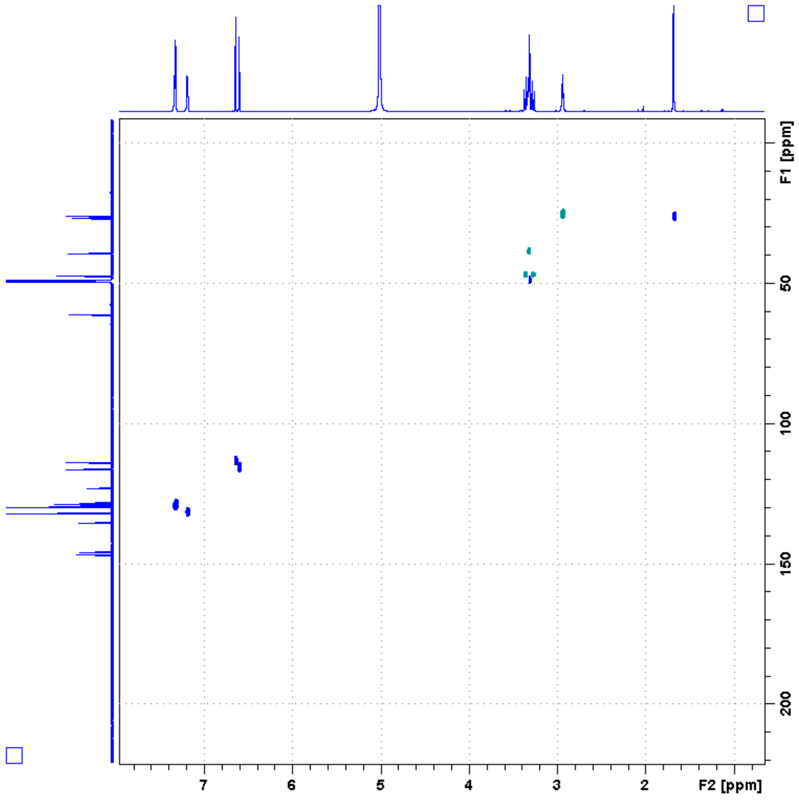

Supplementary Figure 11. NMR spectra of (*S*)-**11** (continued below).

e. HMBC

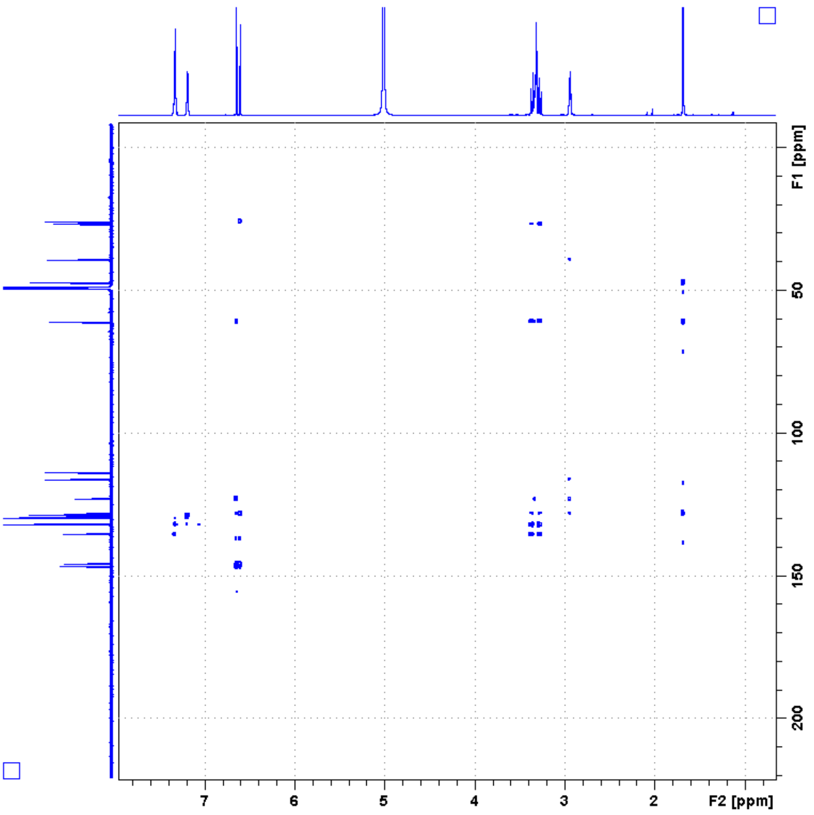

f. NOESY

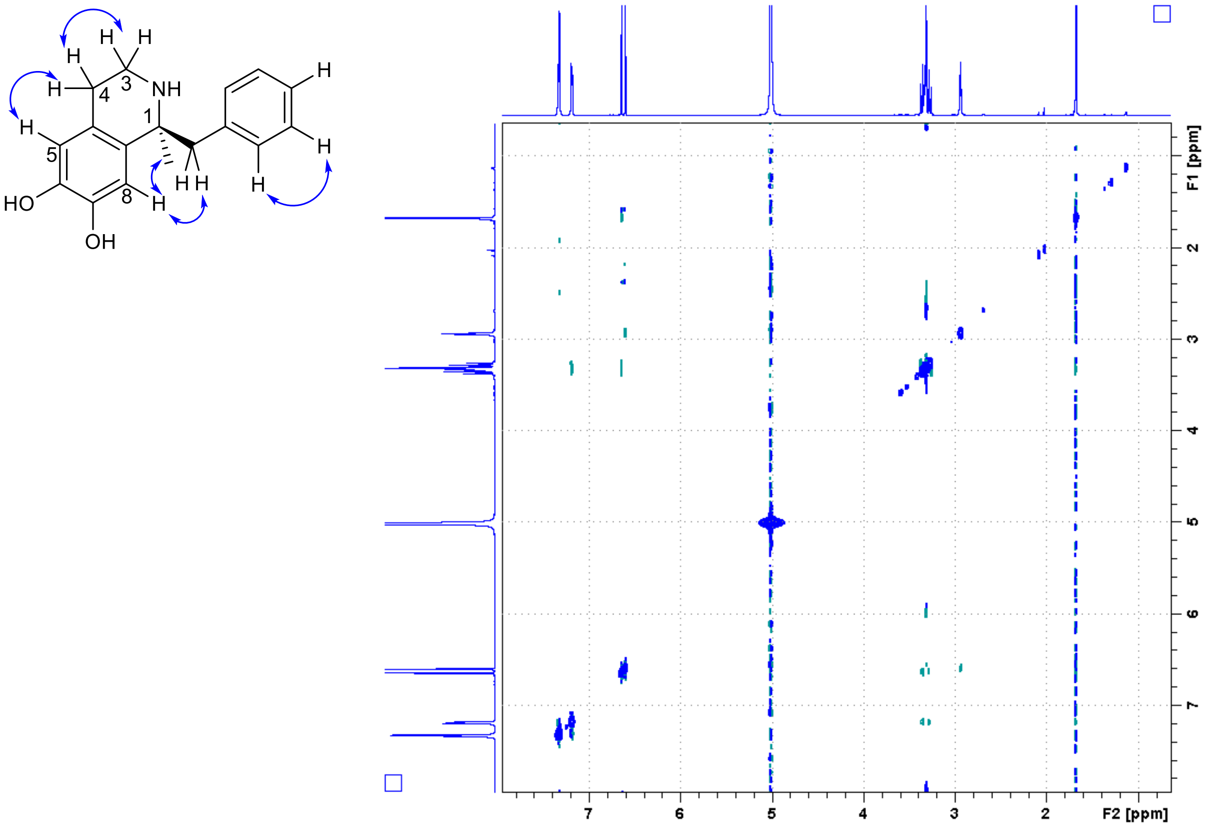

Supplementary Figure 11. NMR spectra of (*S*)-**11**.

a. ^{1}H

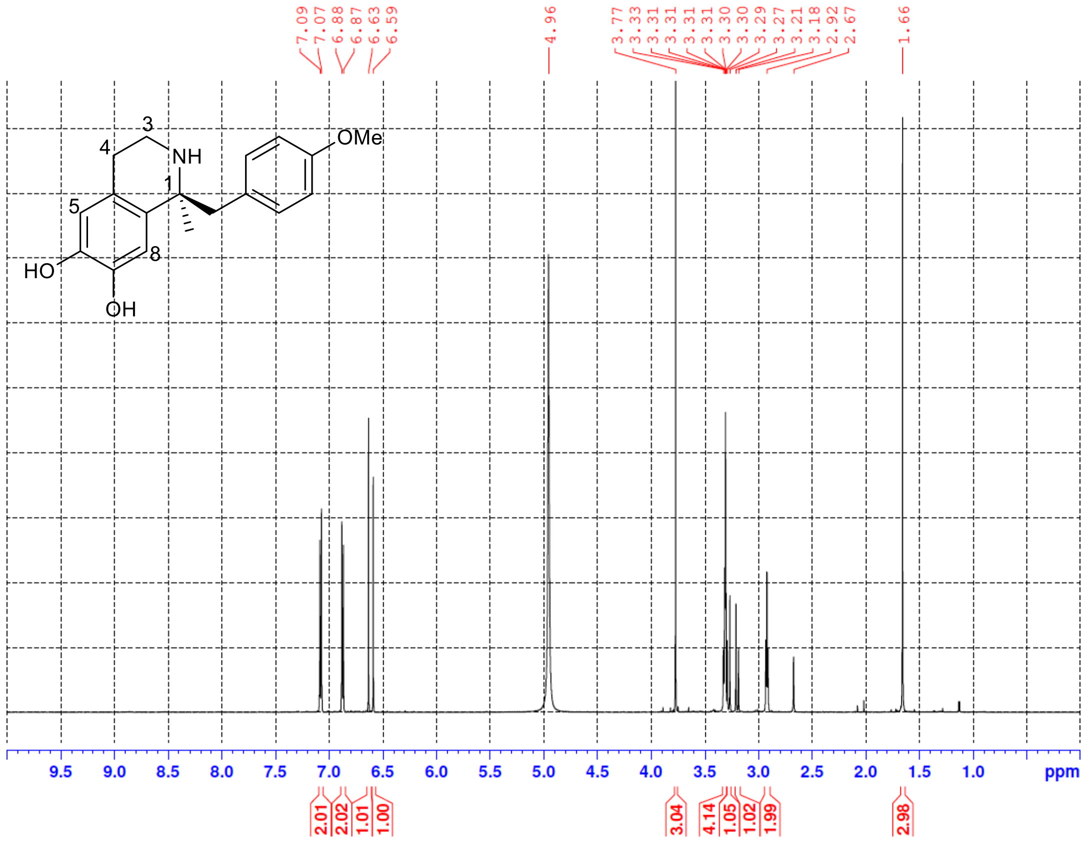

b. ^{13}C DEPT

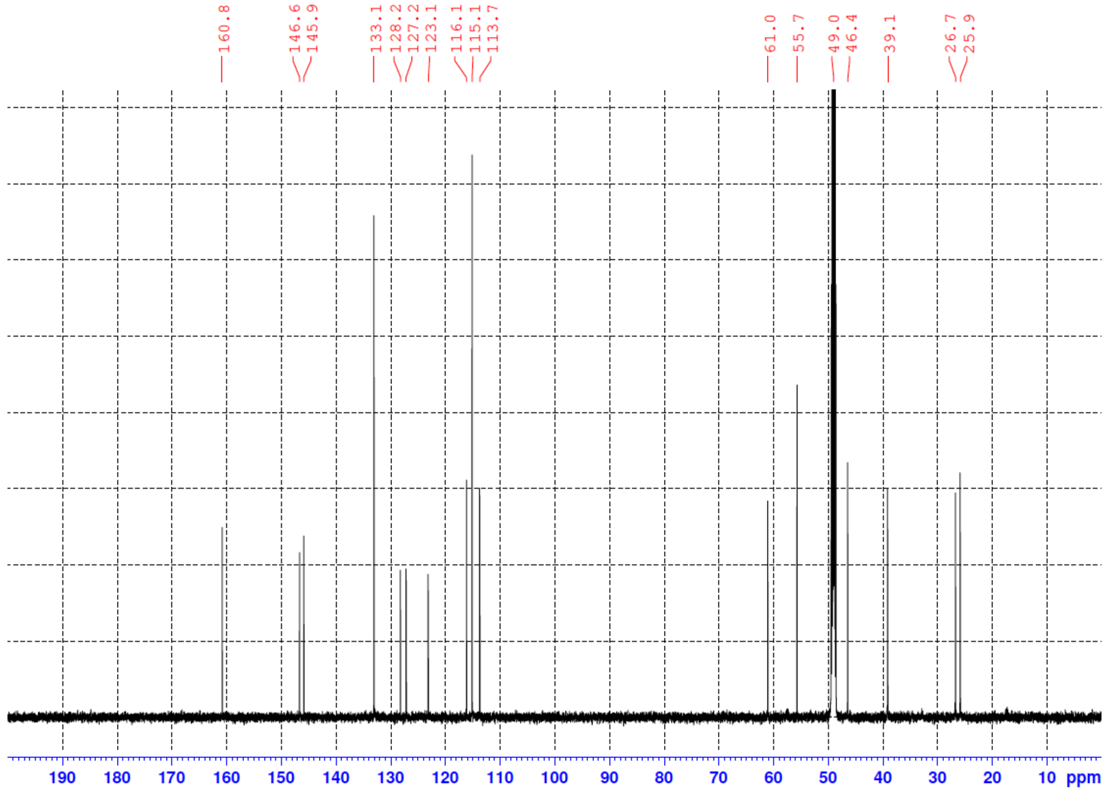

Supplementary Figure 12. NMR spectra of (*S*)-**12** (continued below).

c. COSY

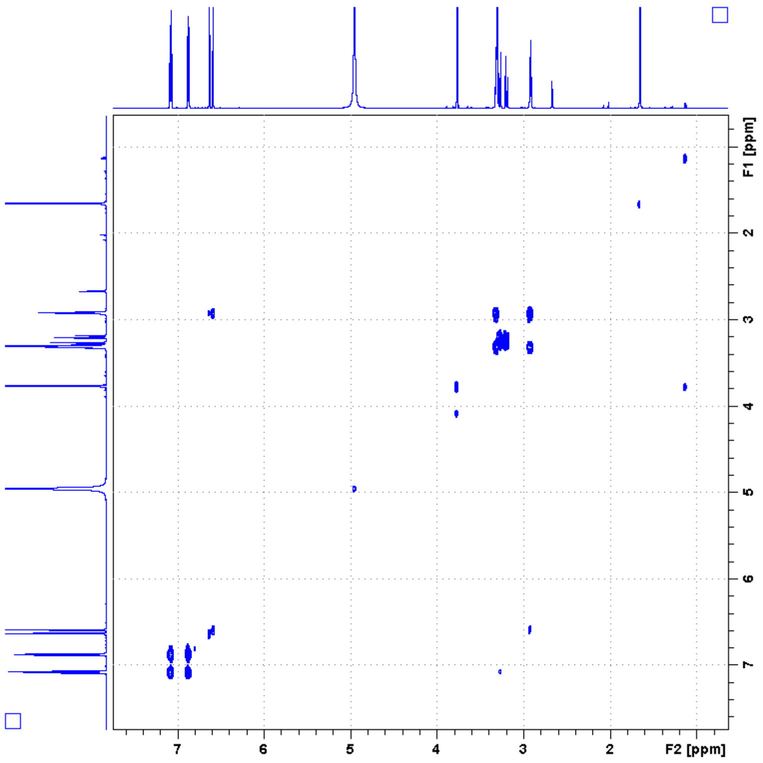

d. HSQC

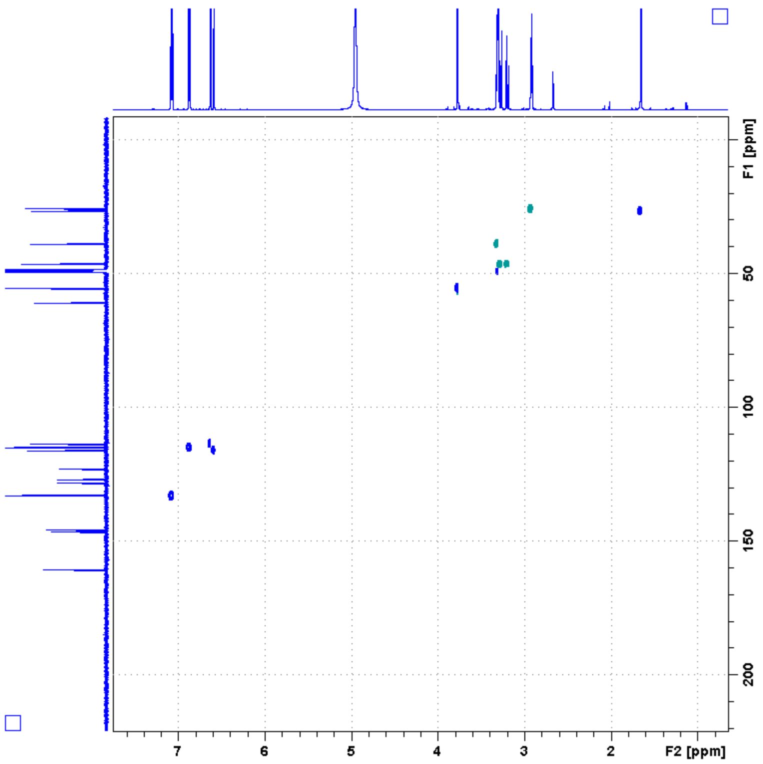

Supplementary Figure 12. NMR spectra of (*S*)-**12** (continued below).

e. HMBC

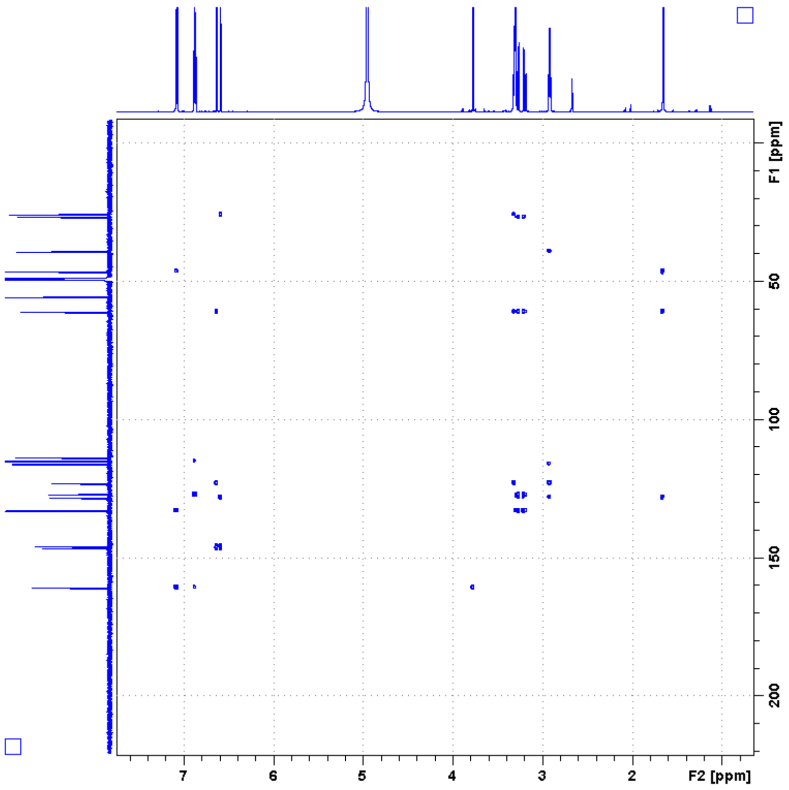

f. NOESY

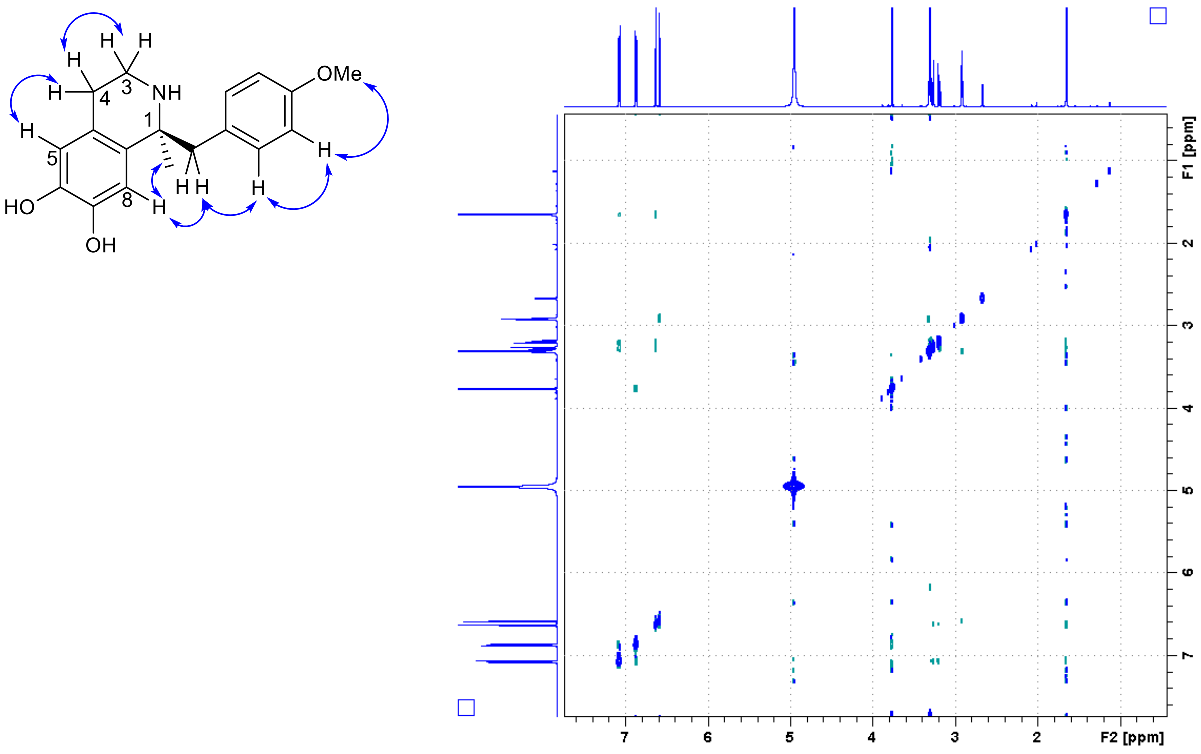

Supplementary Figure 12. NMR spectra of (*S*)-**12**.

a. Step 1 intermediate, ^{1}H

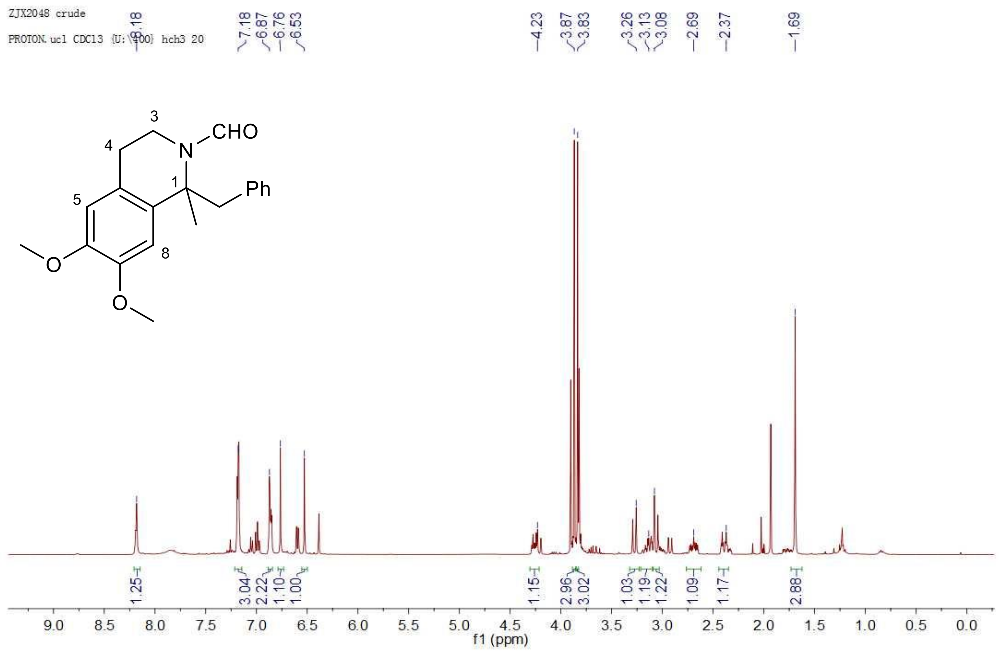

b. Step 2 intermediate, ^{1}H

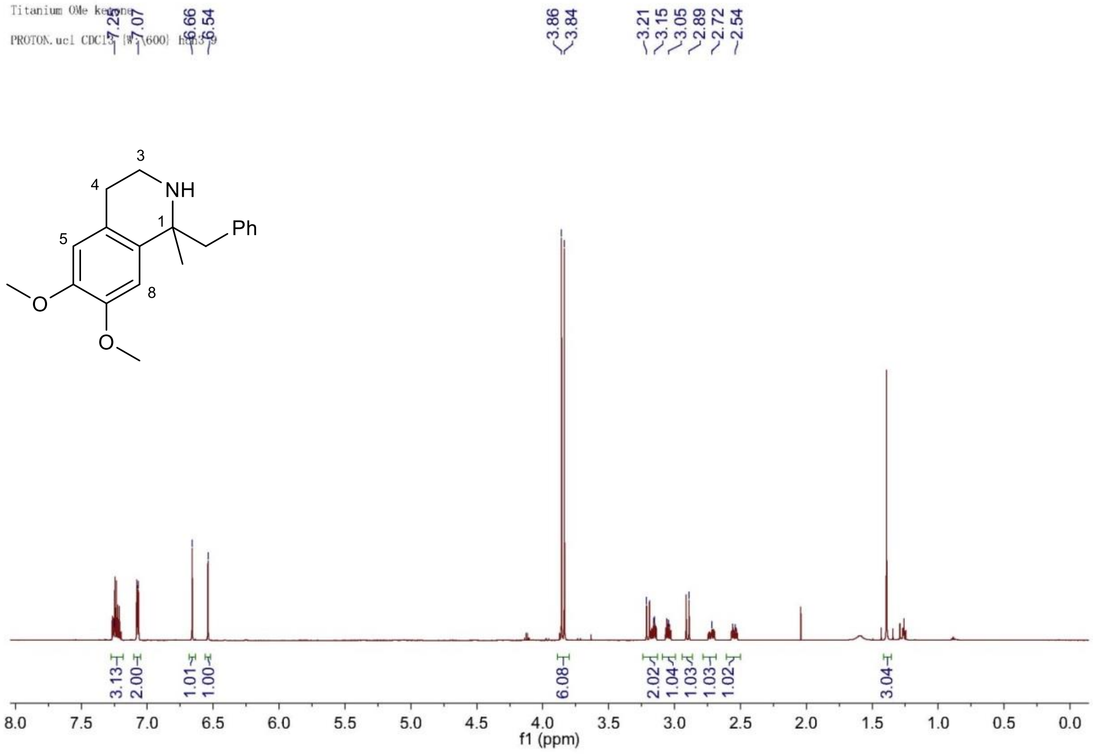

Supplementary Figure 13. NMR spectra of intermediates in *rac*-**11** synthesis (continued below).

c. Step 2 intermediate, ^{13}C

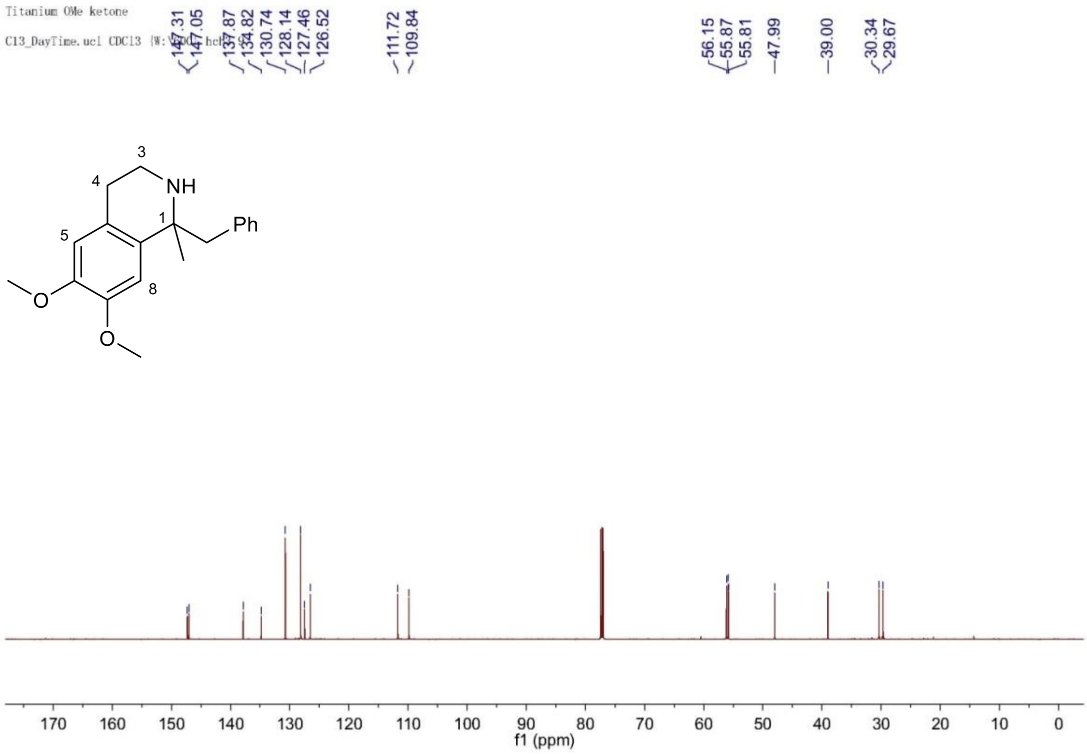

Supplementary Figure 13. NMR spectra of intermediates in *rac*-**11** synthesis.

a. ^{1}H

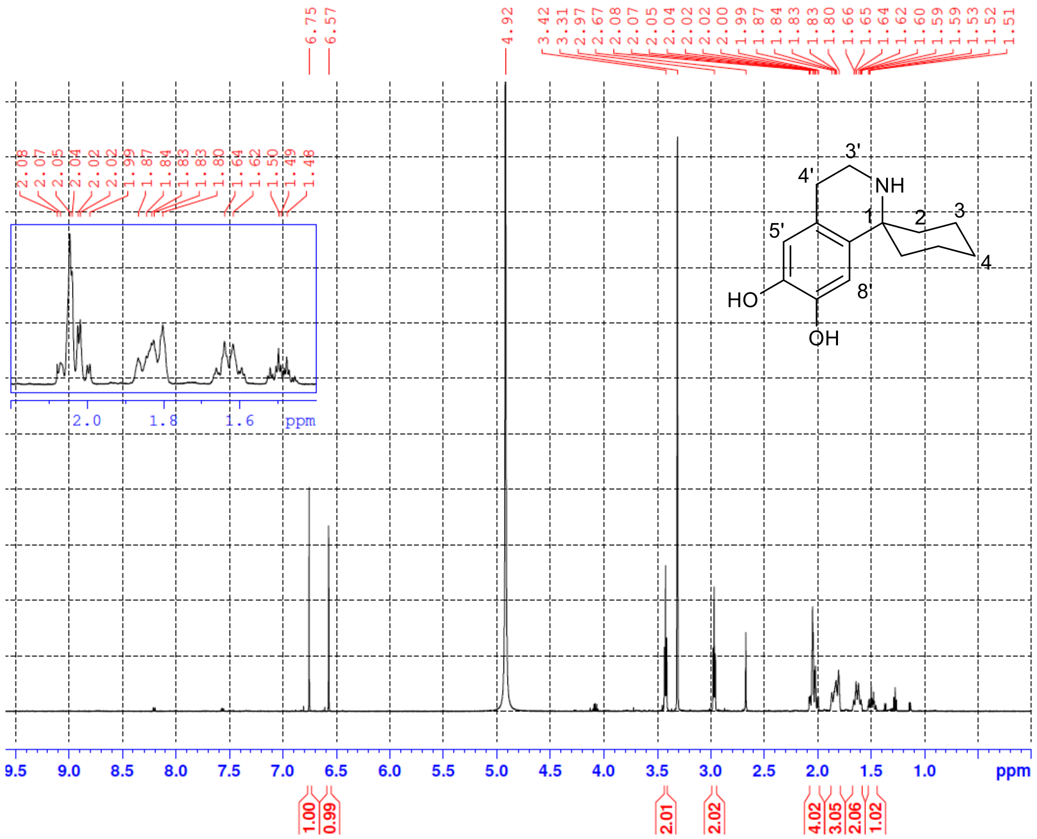

b. ^{13}C DEPT

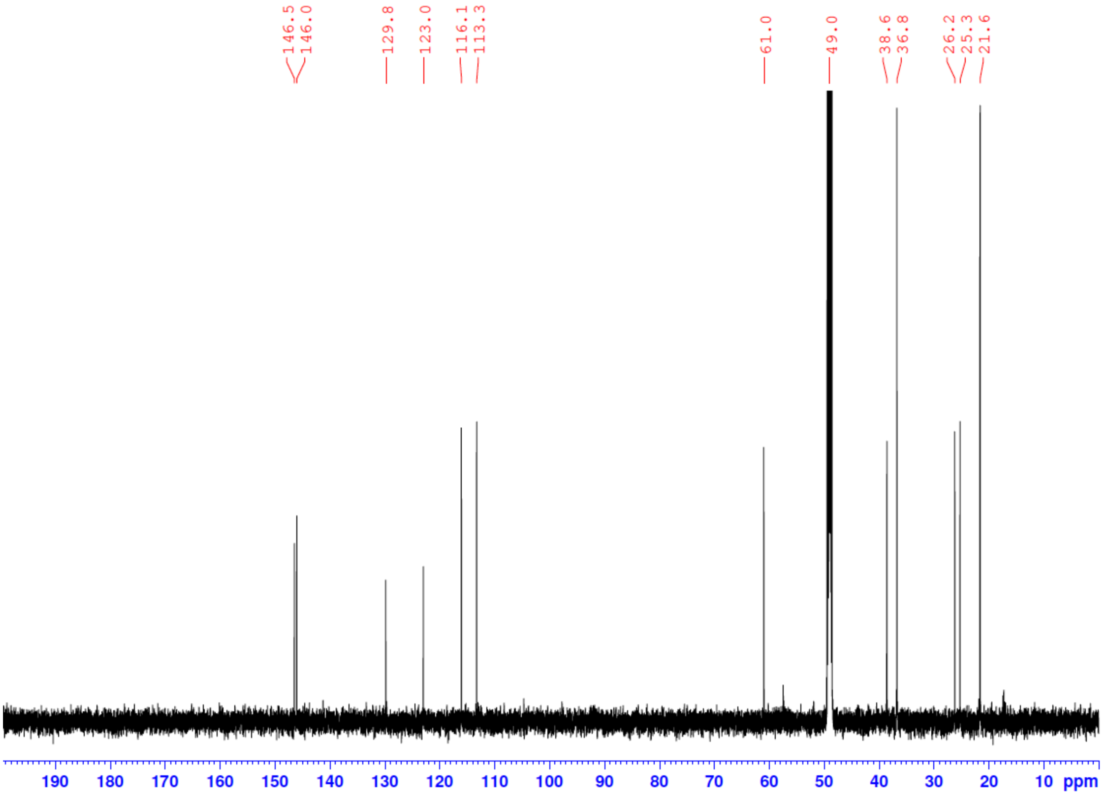

Supplementary Figure 14. NMR spectra of **13** (continued below).

c. COSY

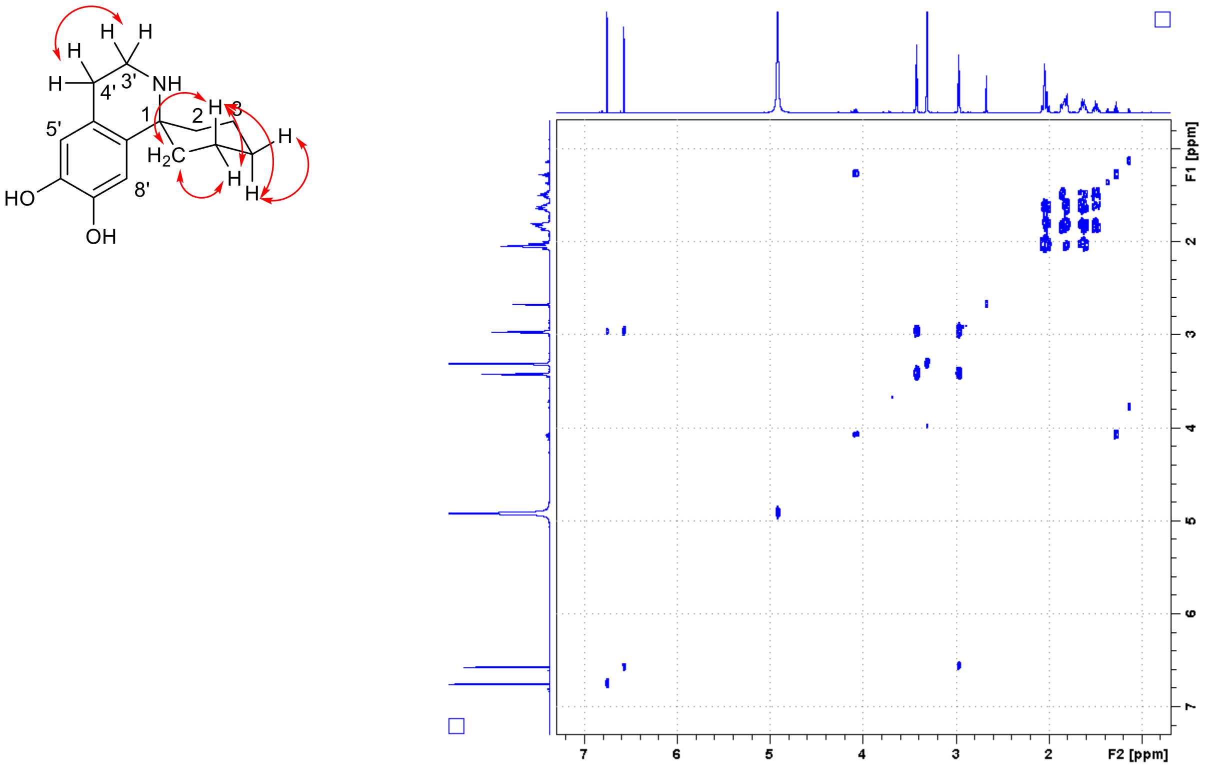

d. HSQC

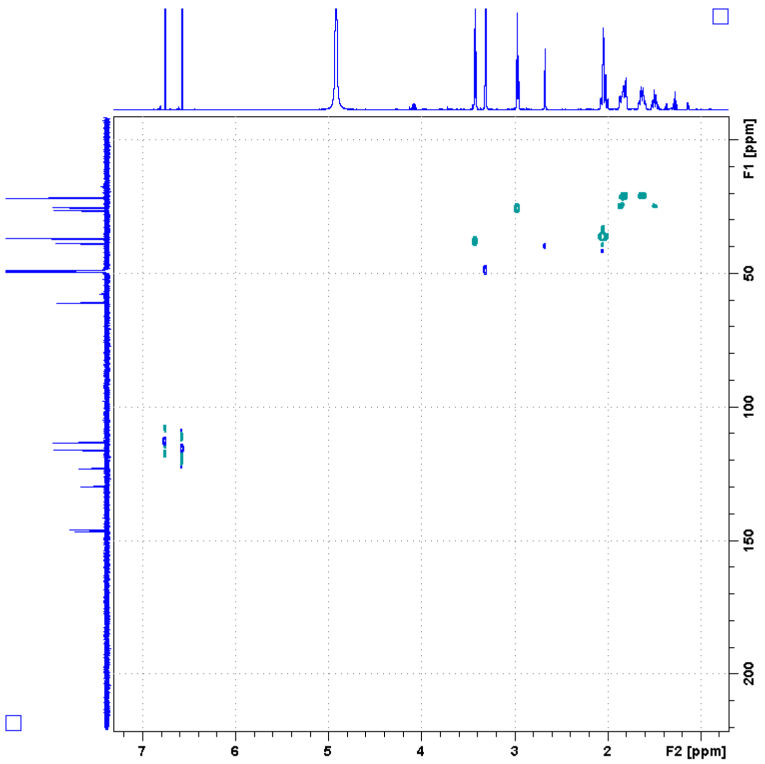

Supplementary Figure 14. NMR spectra of **13** (continued below).

e. HMBC

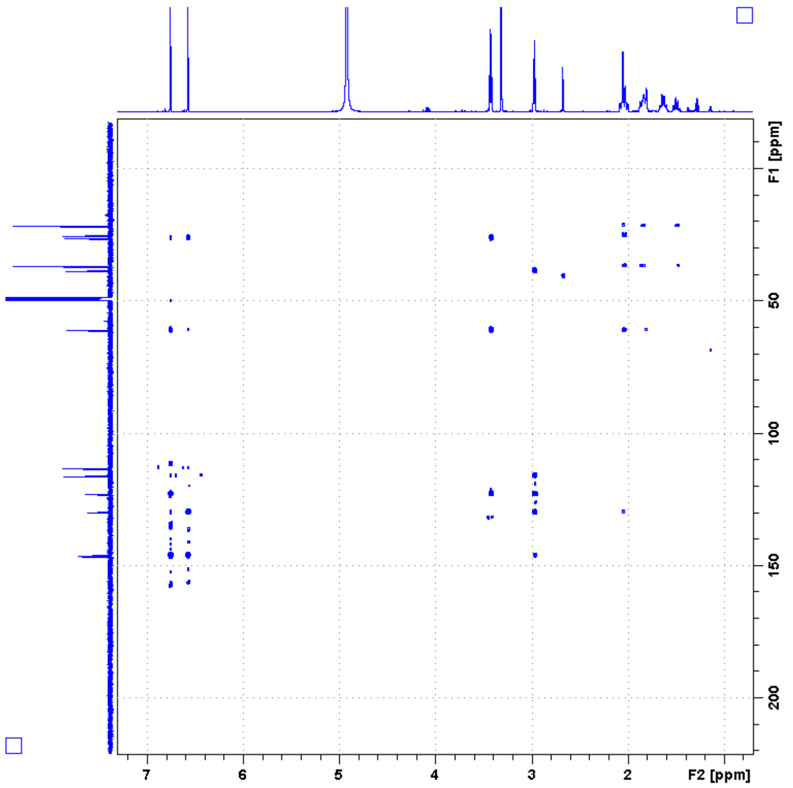

f. NOESY

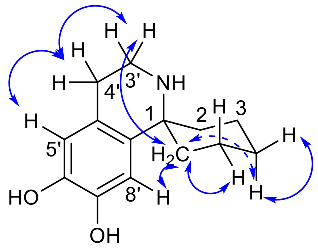

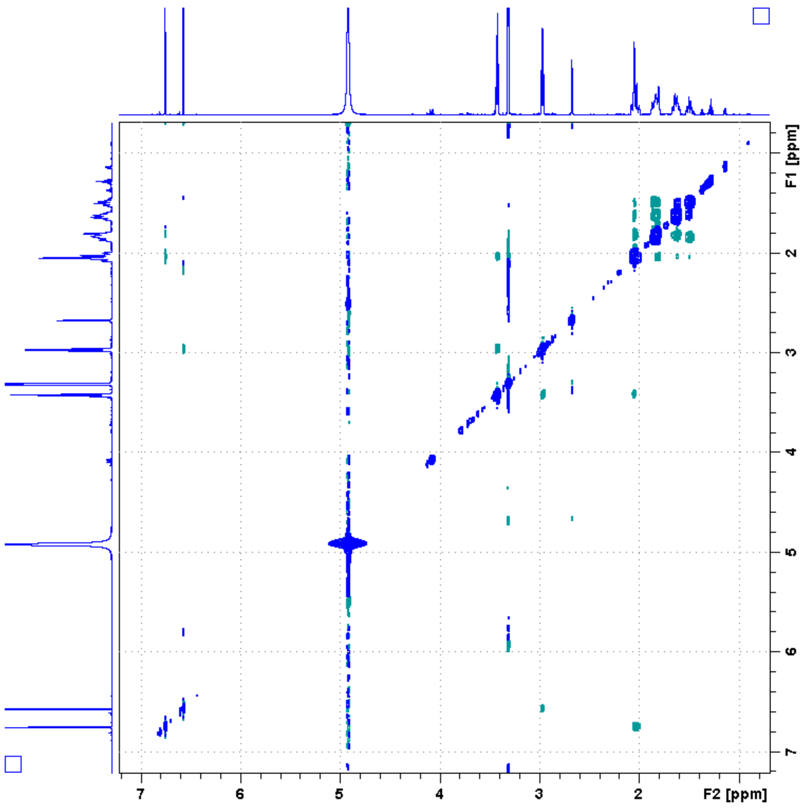

Supplementary Figure 14. NMR spectra of **13**.

a. ^{1}H

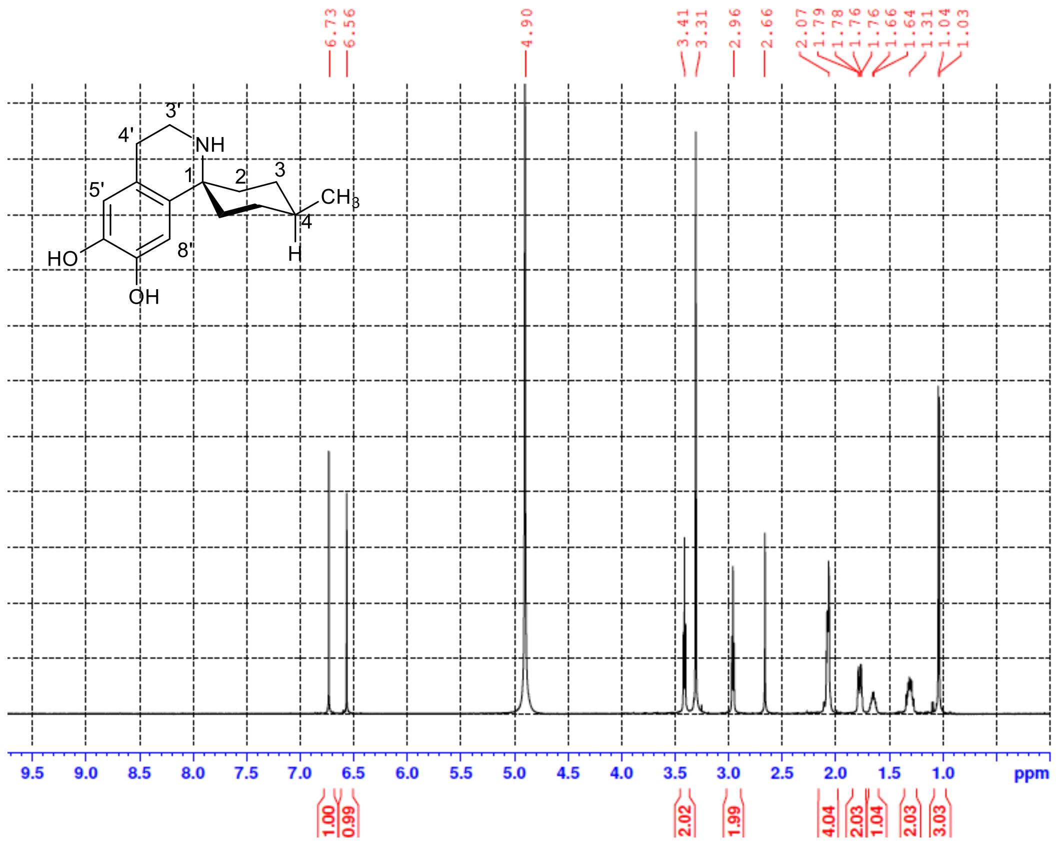

b. ^{13}C DEPT

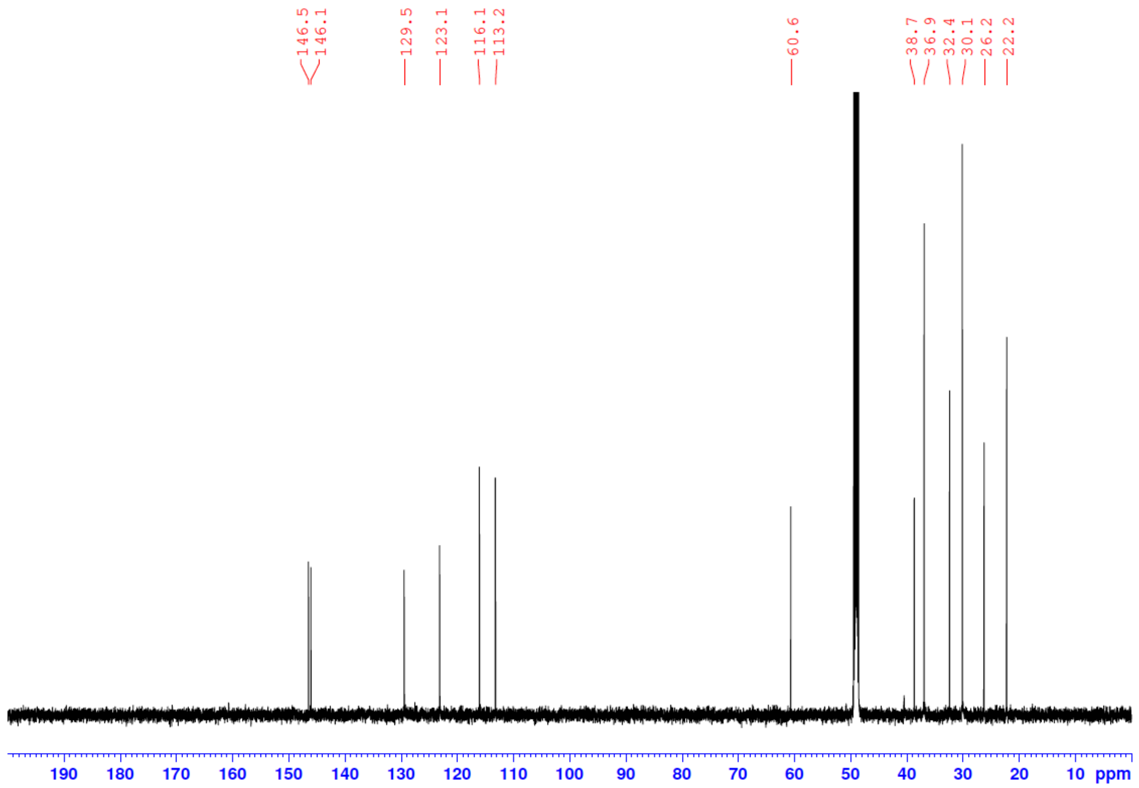

Supplementary Figure 15. NMR spectra of **14** (continued below).

c. COSY

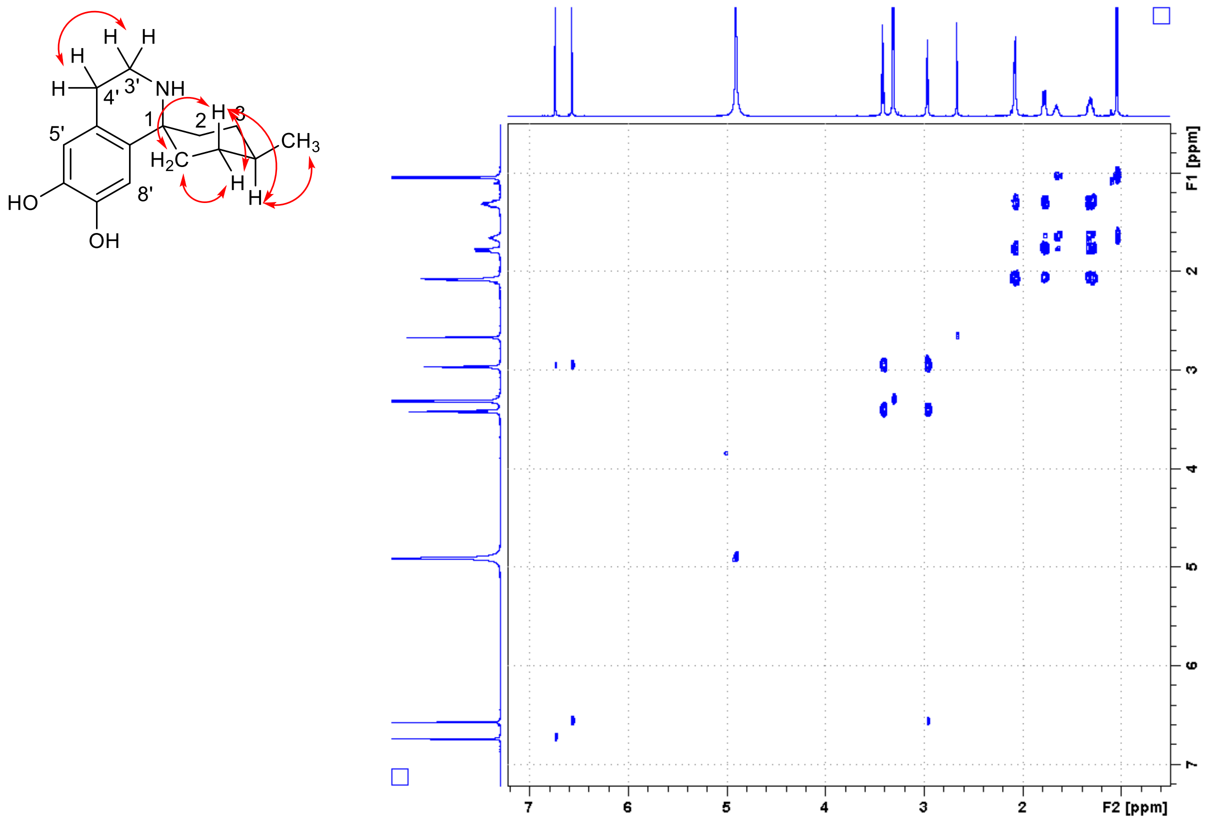

d. HSQC

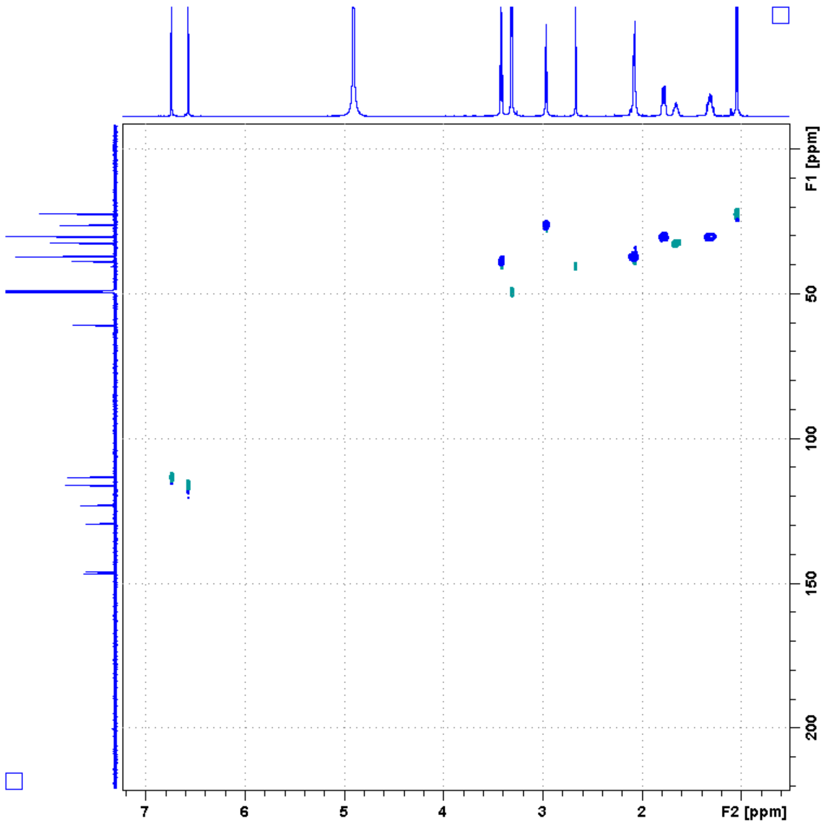

Supplementary Figure 15. NMR spectra of **14** (continued below).

e. HMBC

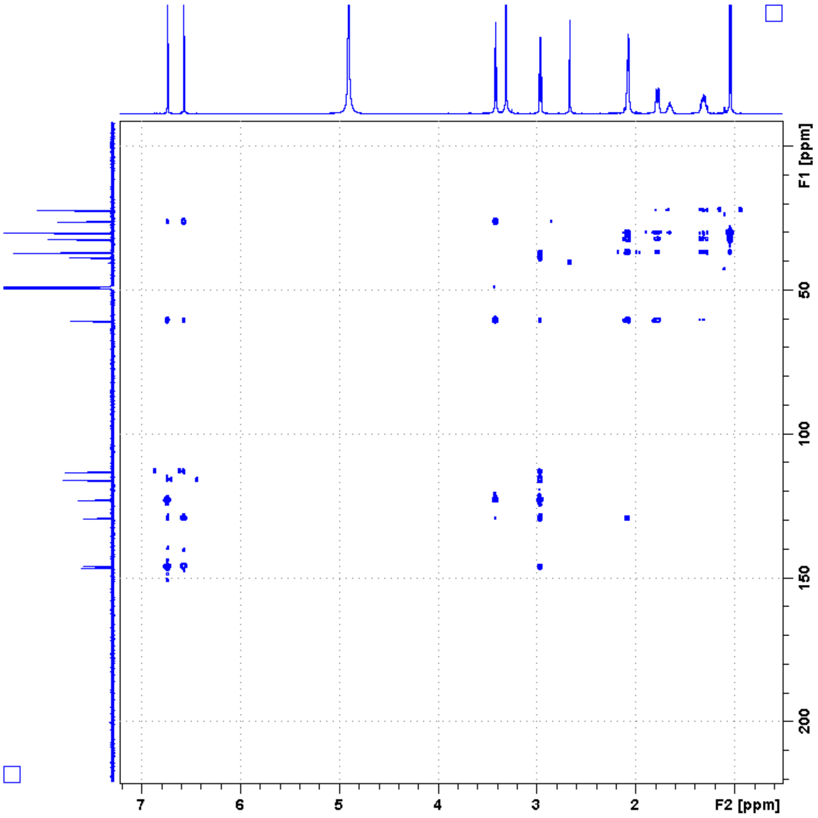

f. NOESY

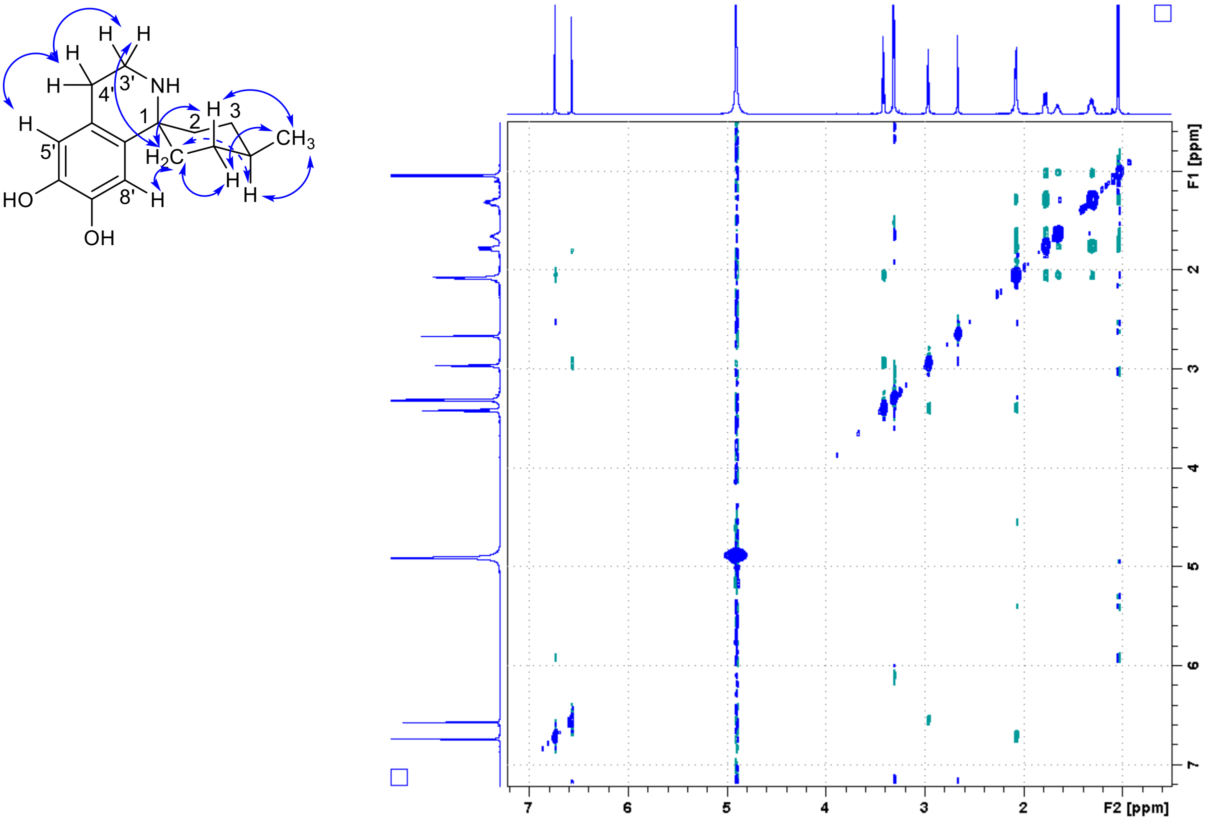

Supplementary Figure 15. NMR spectra of **14**.

a. ^{1}H

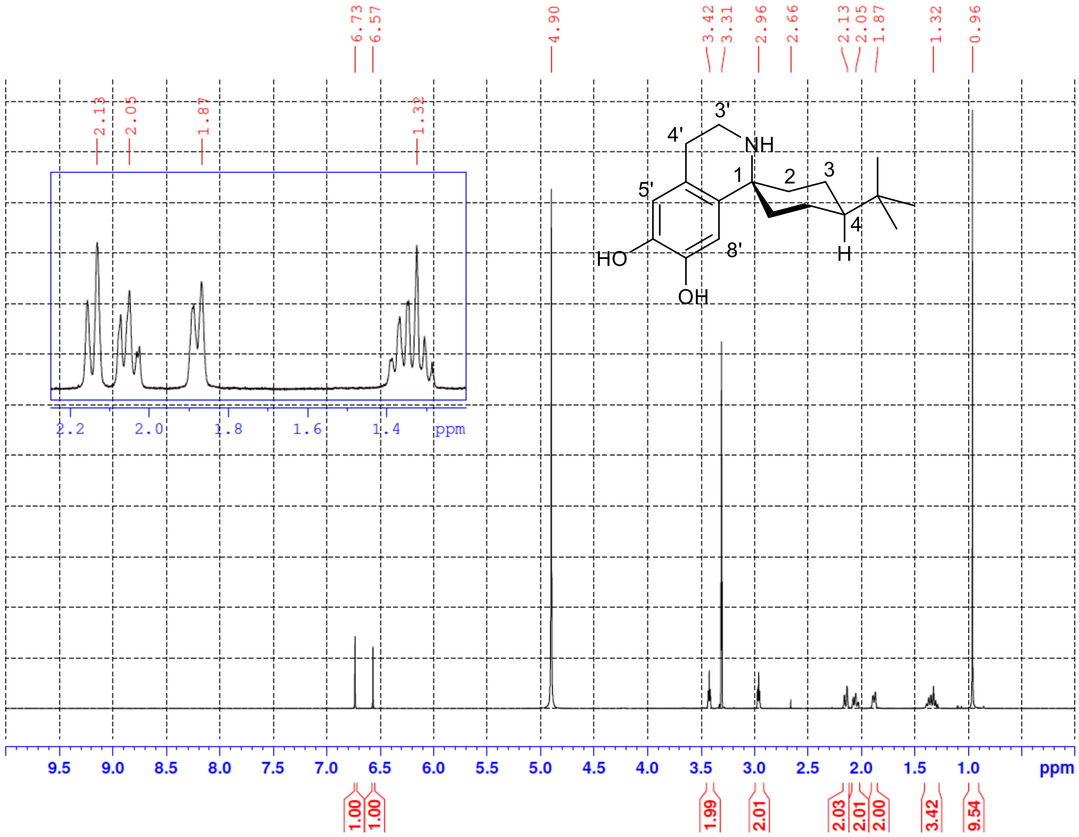

b. ^{13}C DEPT

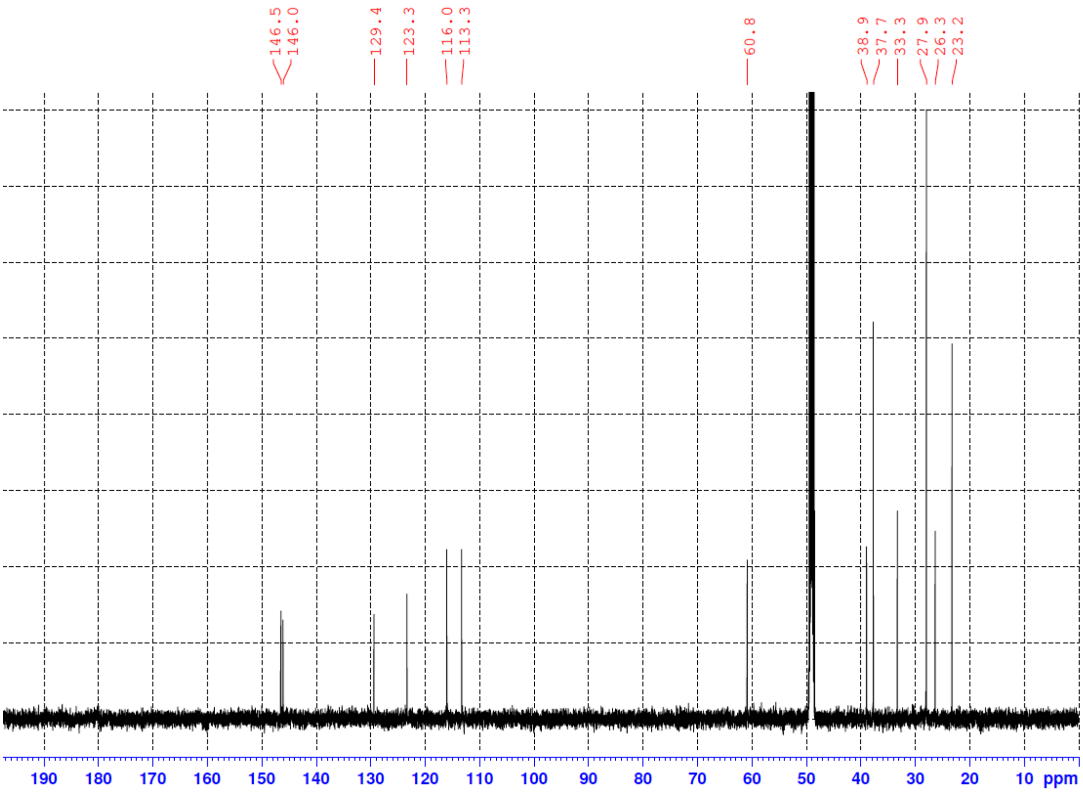

Supplementary Figure 16. NMR spectra of **15** (continued below).

c. COSY

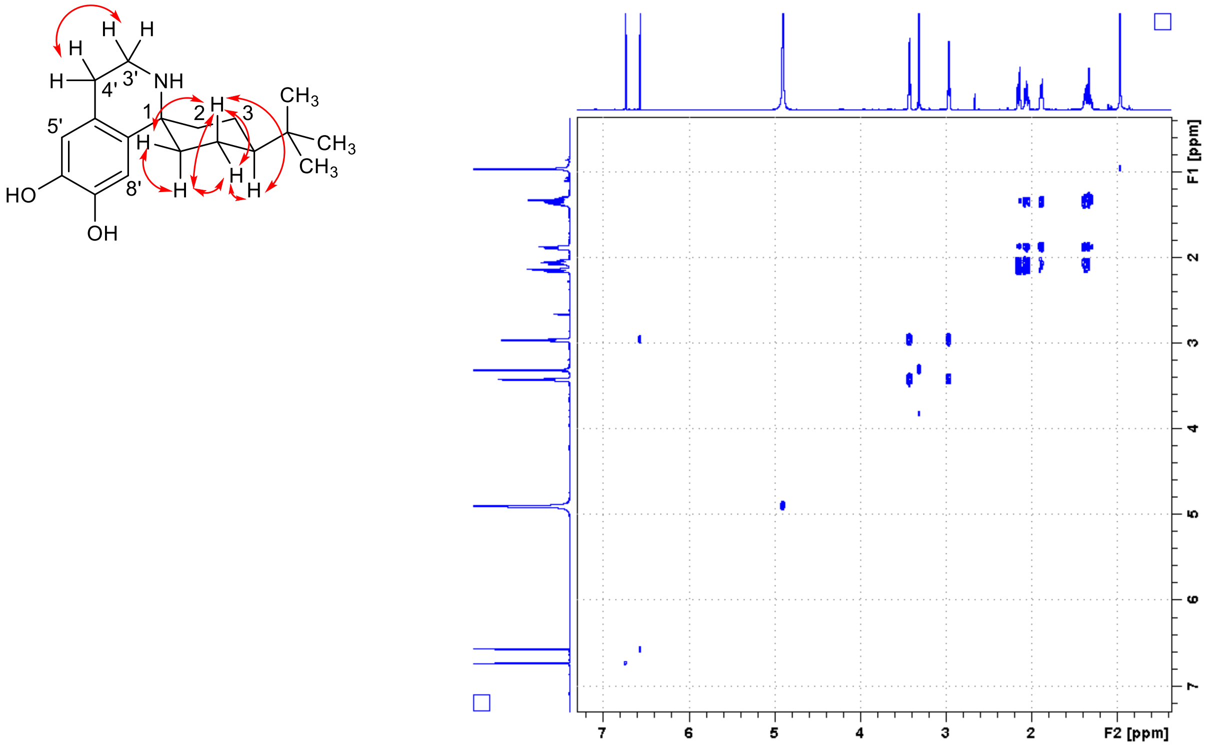

d. HSQC

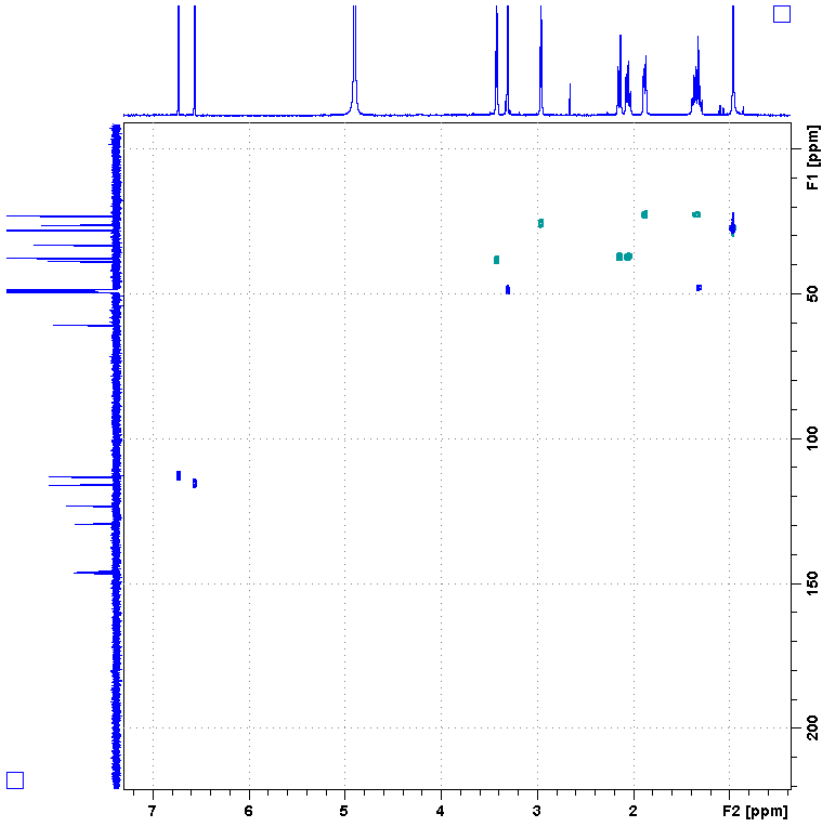

Supplementary Figure 16. NMR spectra of **15** (continued below).

e. HMBC

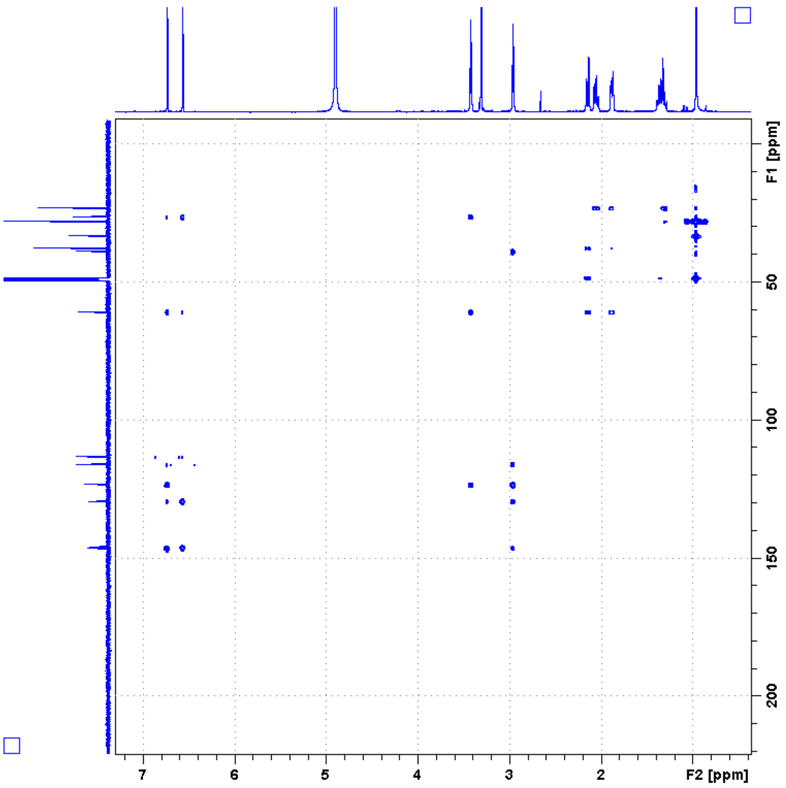

f. NOESY

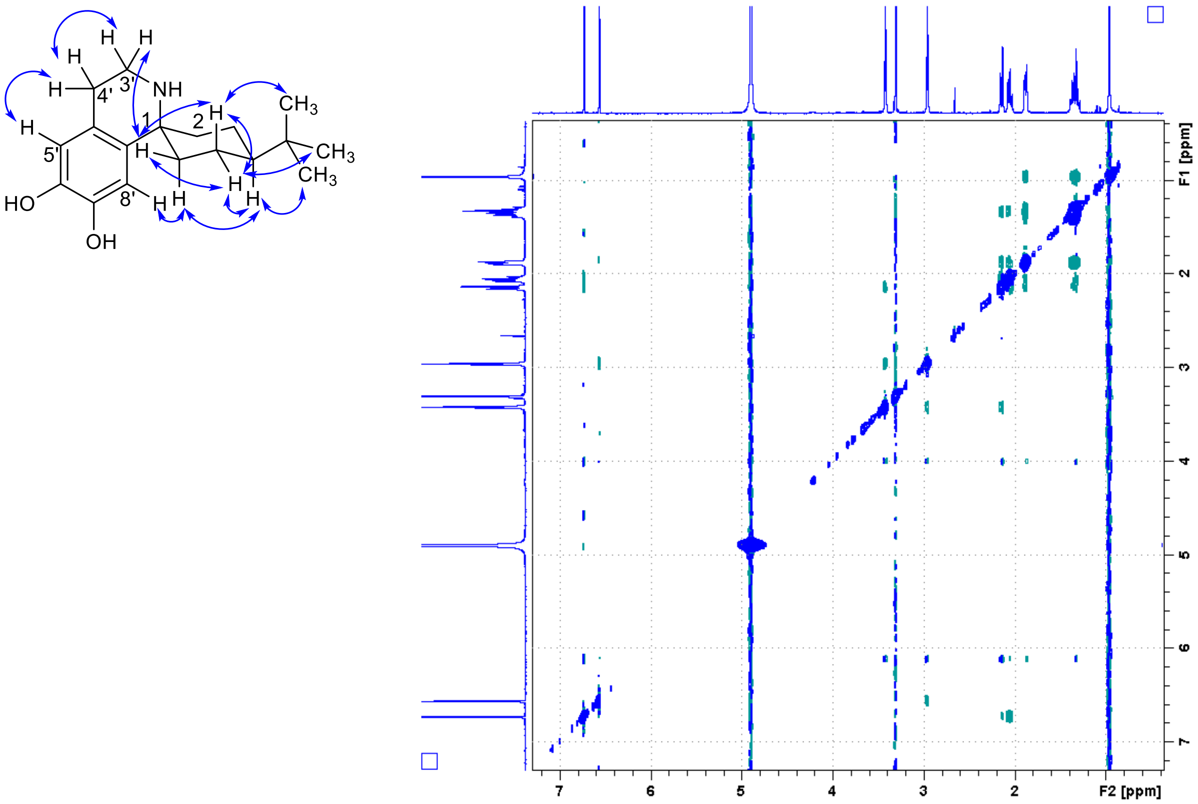

Supplementary Figure 16. NMR spectra of **15**.

a. ^{1}H

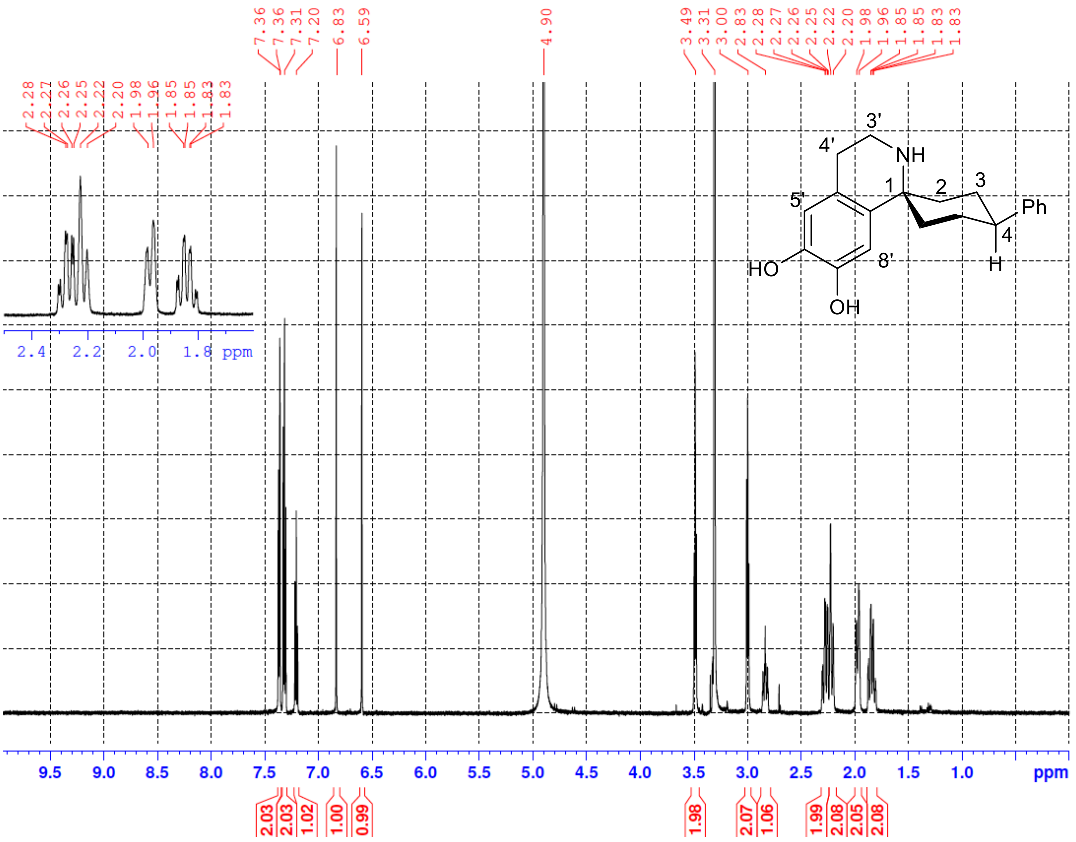

b. ^{13}C DEPT

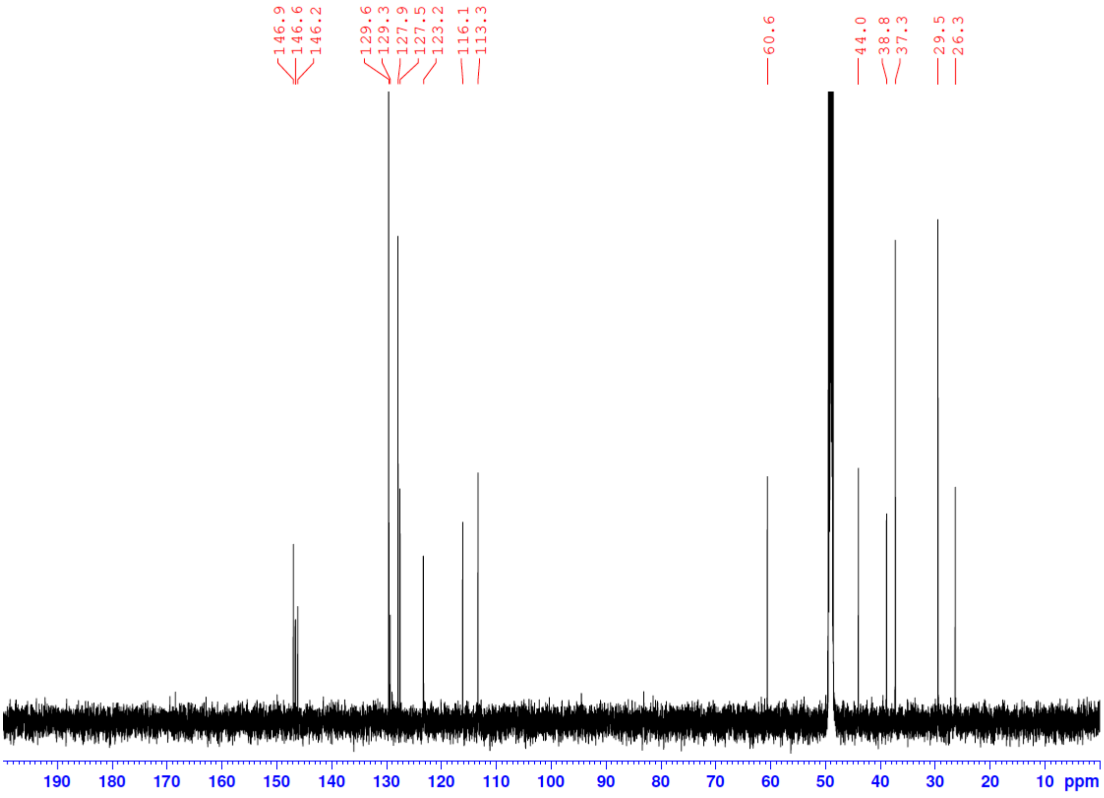

Supplementary Figure 17. NMR spectra of **16** (continued below).

c. COSY

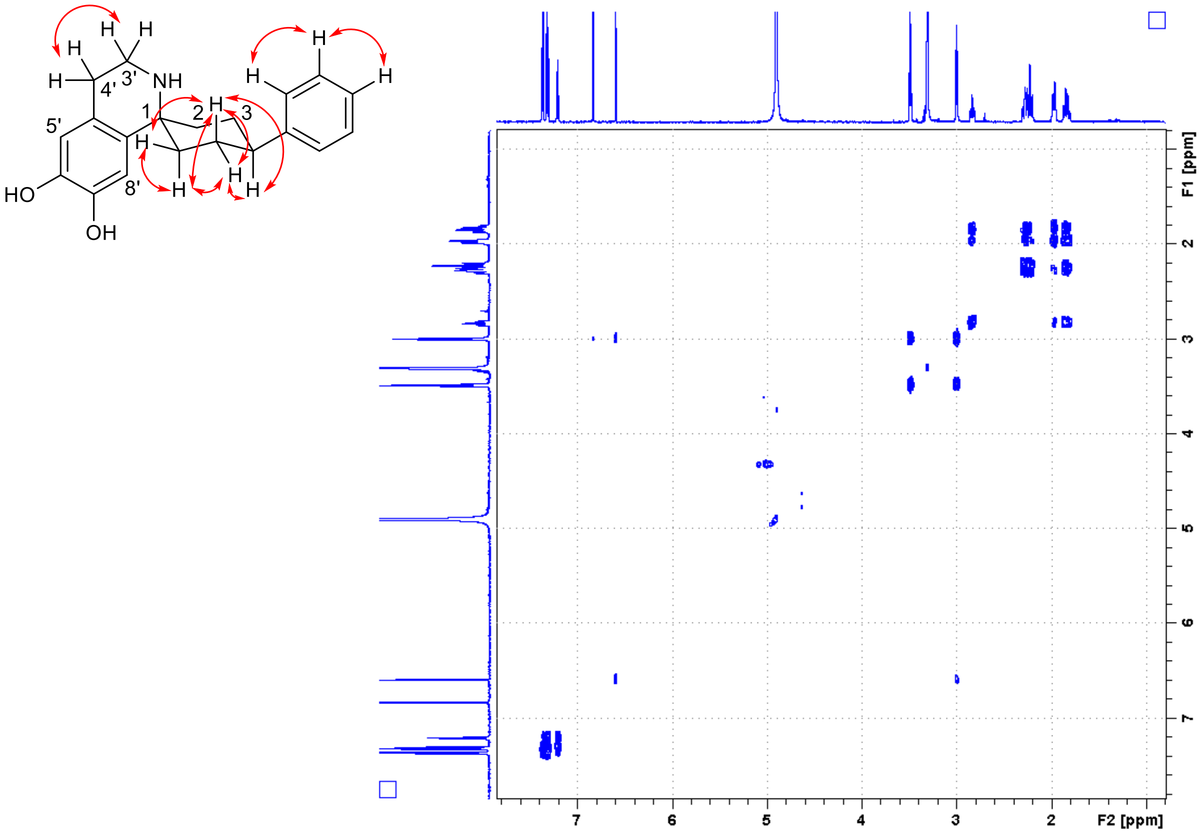

d. HSQC

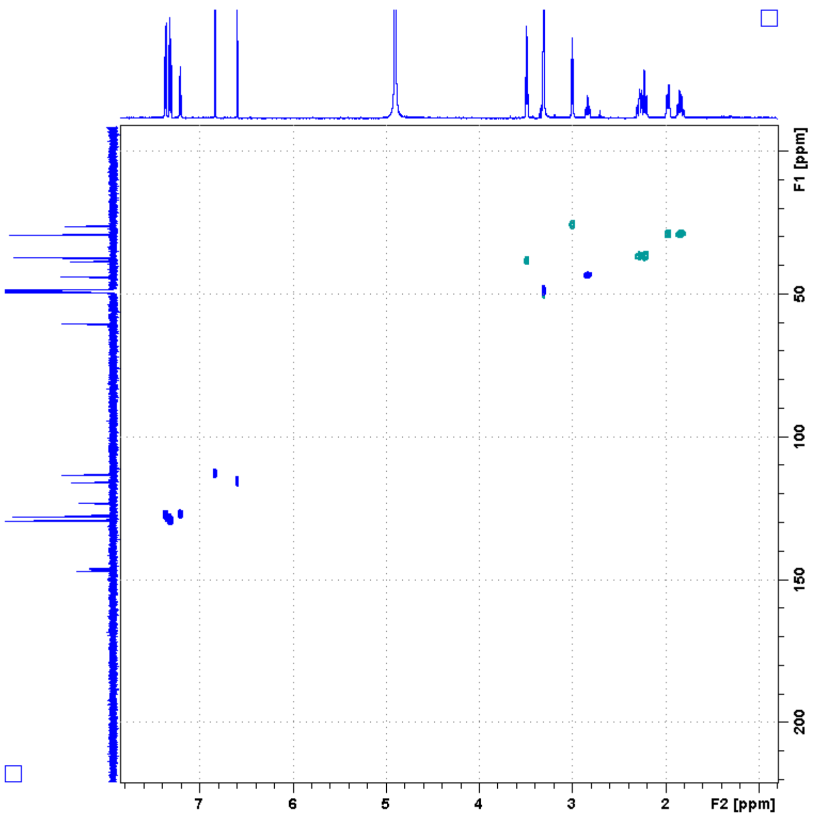

Supplementary Figure 17. NMR spectra of **16** (continued below).

e. HMBC

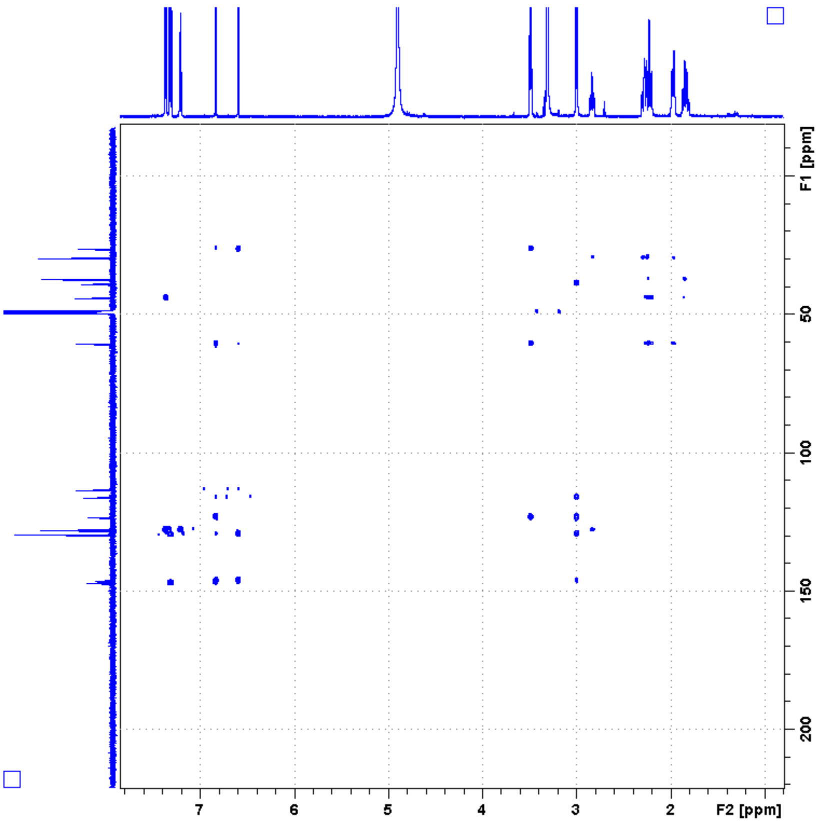

f. NOESY

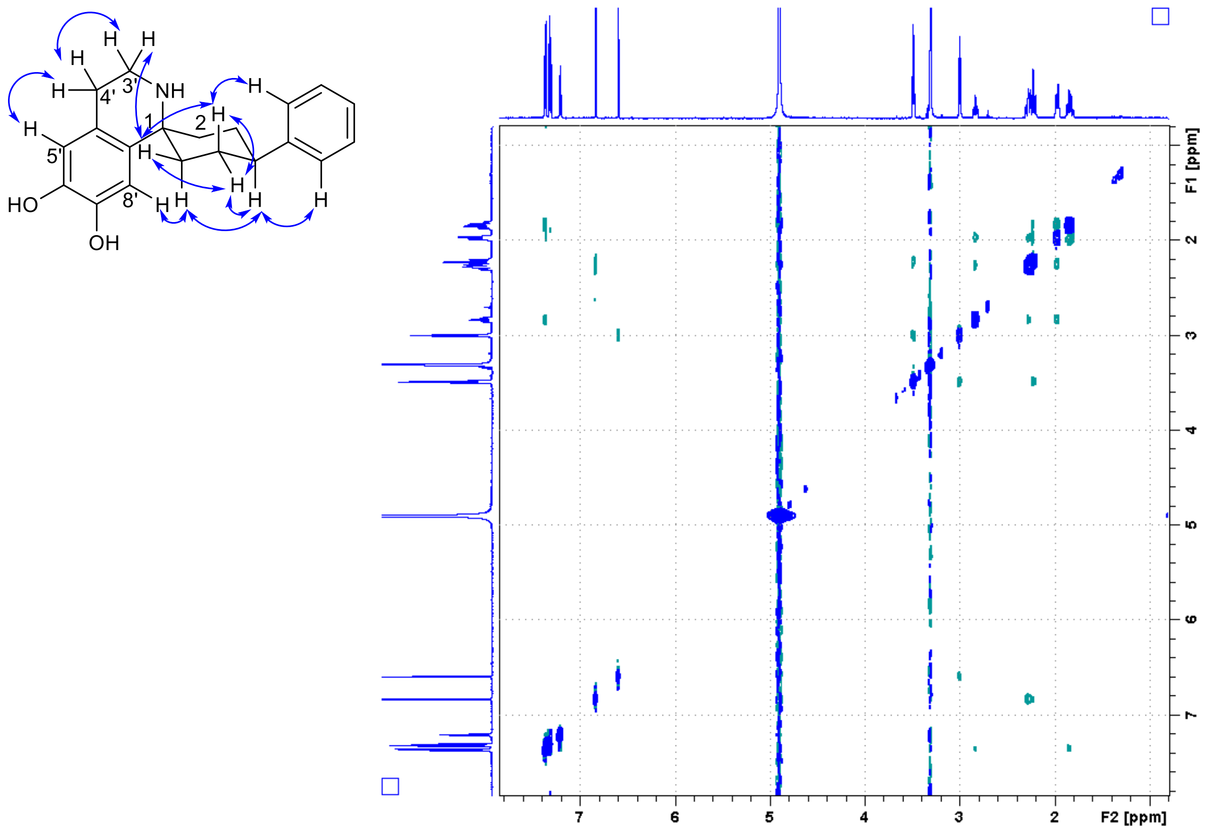

Supplementary Figure 17. NMR spectra of **16**.

a. ^{1}H

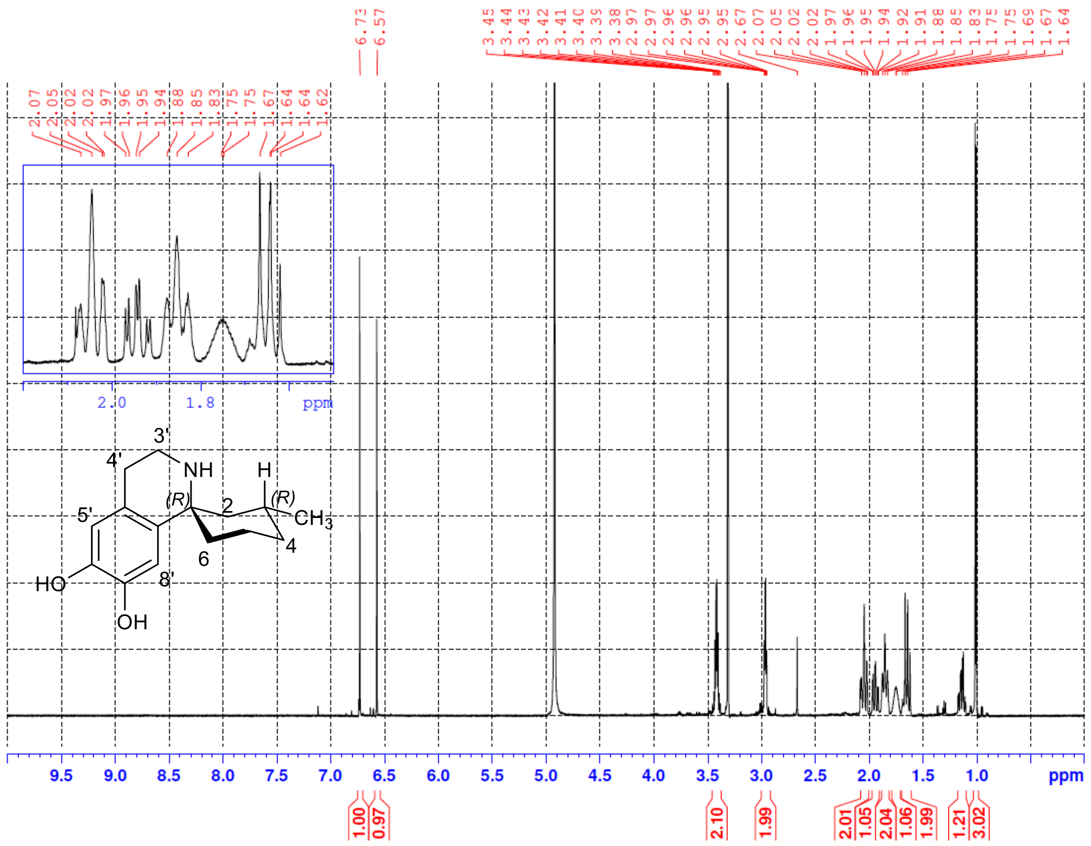

b. ^{13}C DEPT

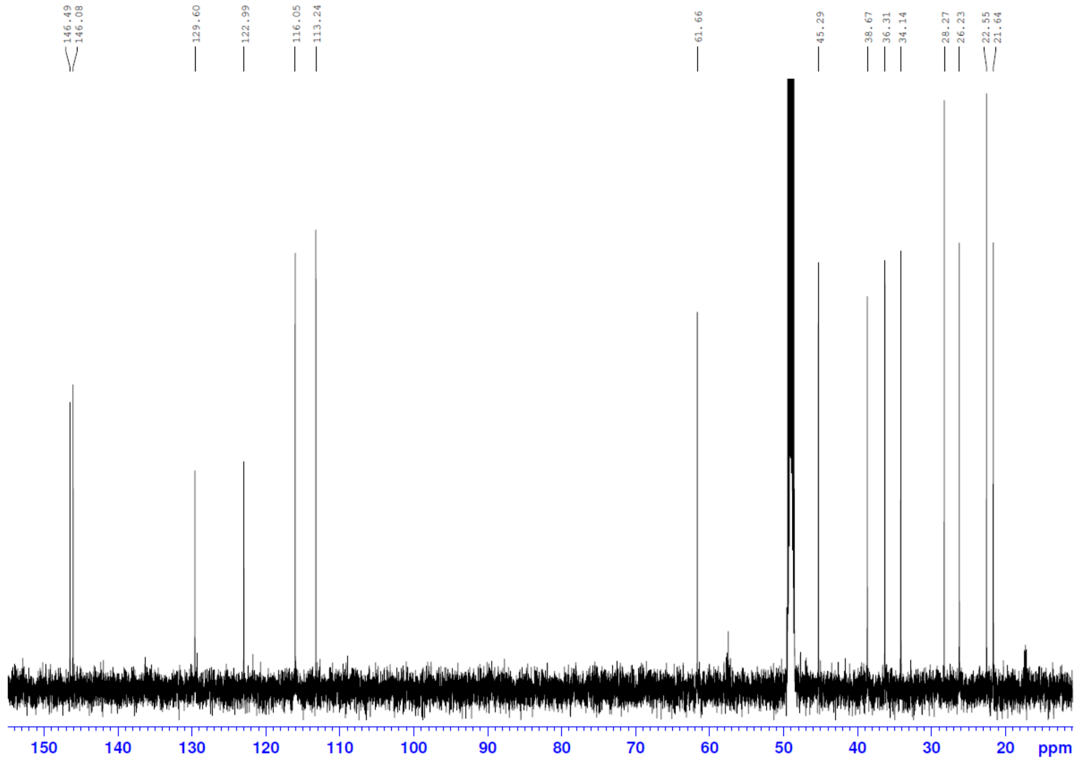

Supplementary Figure 18. NMR spectra of (*R*)-**17** (continued below).

c. COSY

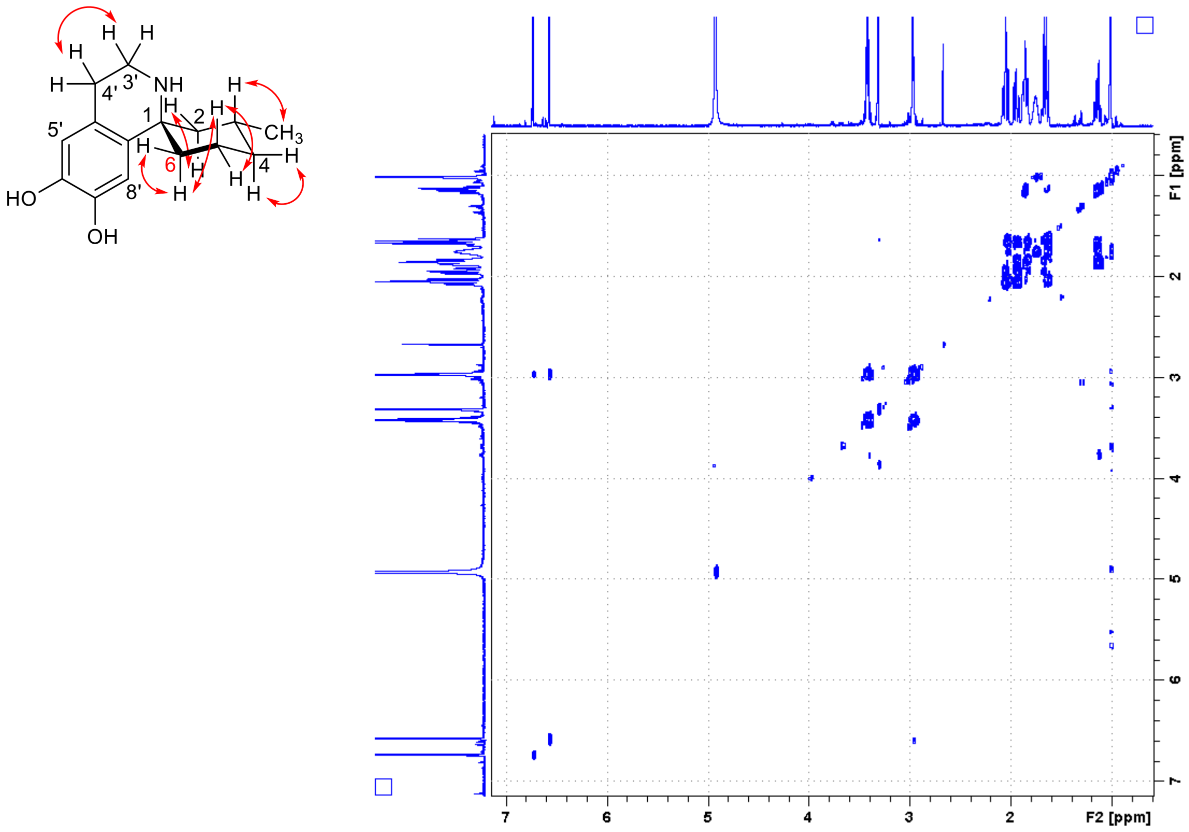

d. HSQC

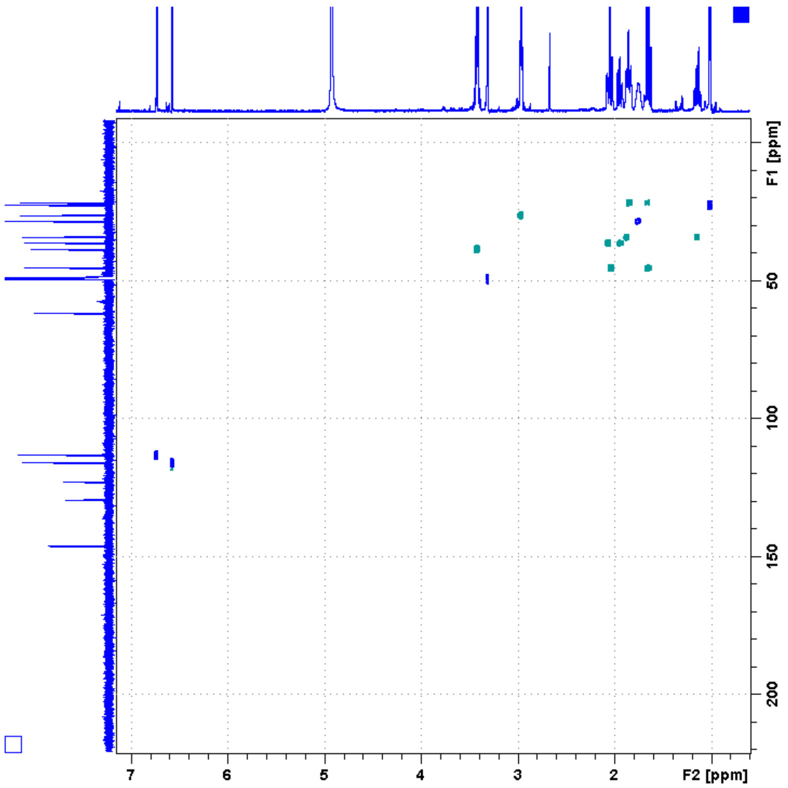

Supplementary Figure 18. NMR spectra of (*R*)-**17** (continued below).

e. HMBC

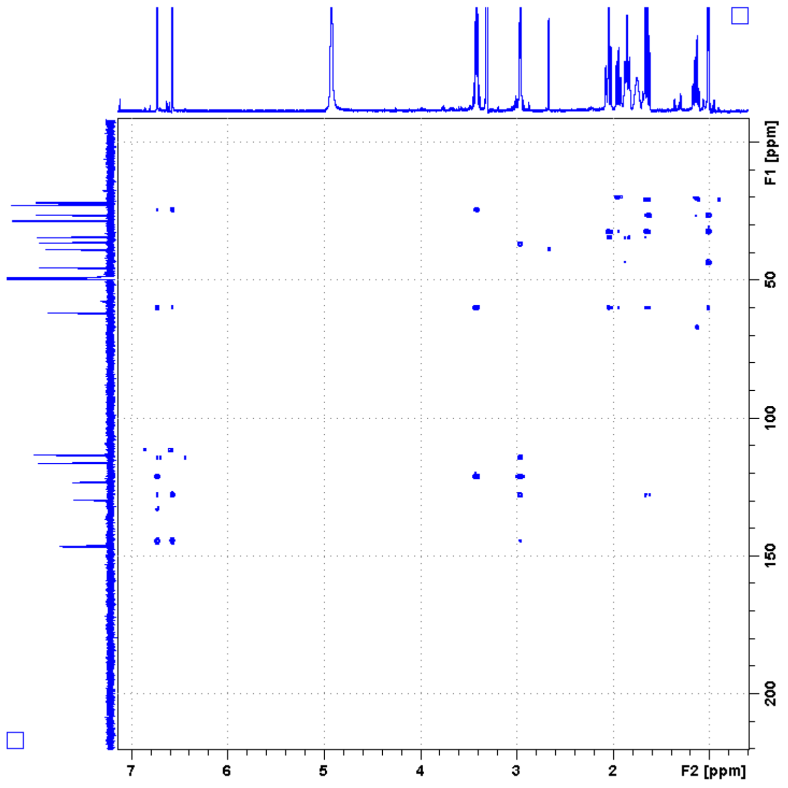

f. NOESY

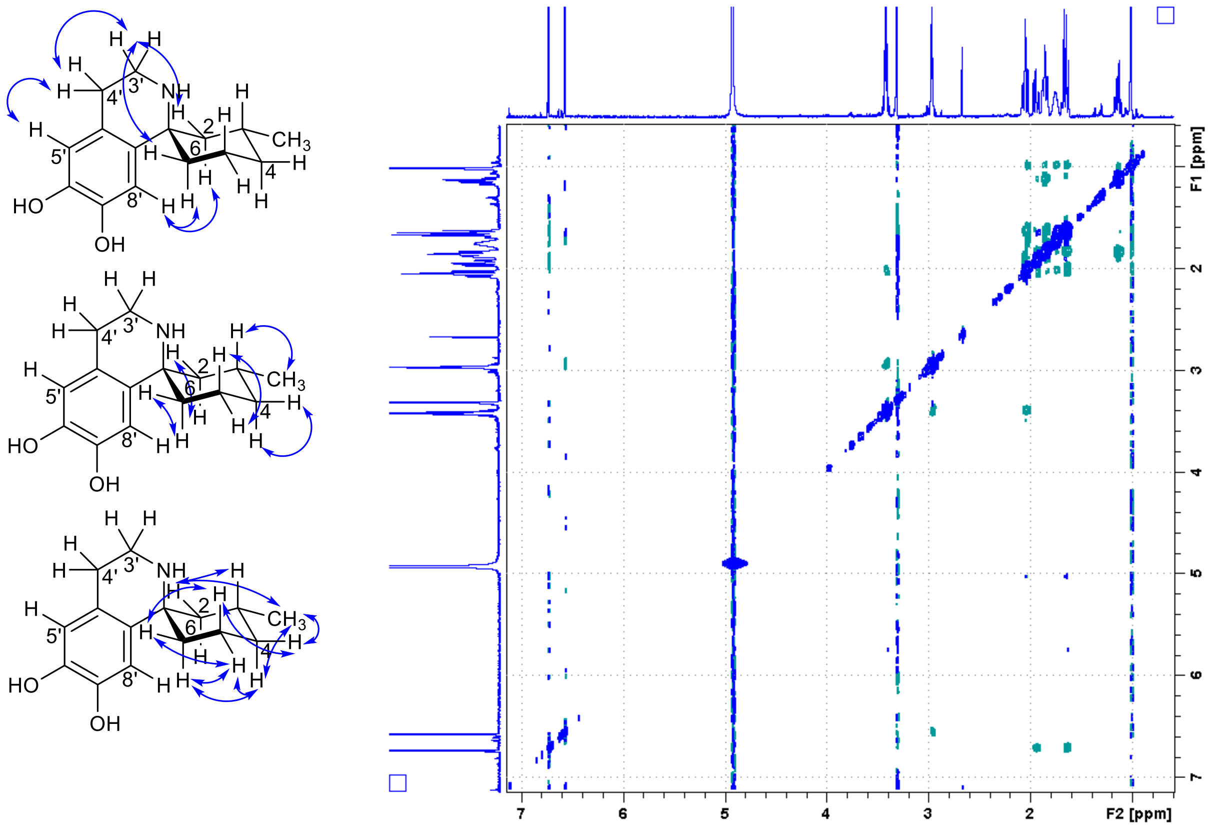

Supplementary Figure 18. NMR spectra of (*R*)-**17**.

Iminium intermediate	Rank	Affinity kcal/mol	Figure
4-HPAA	2	-7.2	7a
cyclohexanone **6**	2	-6.4	S7a
4-methylcyclohexanone **7**	3	-6.7	S7b
4-*tert*-butylcyclohexanone **8**	1	-7.4	S7c
4-phenylcyclohexanone **9**	1	-8.0	7b
(3*R*)-methylcyclohexanone* (*R*)-**10**	5	-6.1	S7d
phenylacetone (*trans*) **4**	2	-7.4	7c
phenylacetone (*cis*) **4**	3	-6.3	7d
4-methoxyphenylacetone (*trans*) **5**	3	-7.2	S7e
4-methoxyphenylacetone (*cis*) **5**	2	-6.9	S7f

Supplementary Table 1. Computational docking output. Mechanistically relevant ‘dopamine-first’ binding modes were selected from 9 ranked binding modes. The rank of the relevant binding mode is described, along with the predicted affinity. Reaction intermediates (Main **Figure 7** and **Supplementary Fig. 7**) were energy optimized using MM2 energy minimization. The receptor used was subunit A from the *Tf*NCS crystal structure 2VQ5 (residues 40 to 191, with ligands removed). Docking was performed with AutoDock Vina (exhaustiveness = 10). Docking box parameters (x,y,z): center (22.27, 21.16,-27.51), size (23.56, 17.37, 21.78). *The exception to the method was the iminium intermediate f (**17**): no MM2 minimization was conducted and modified docking box parameters were used (center (22.15, 21.15,-27.94), size (16.61, 16.56, 13.43)). Reported binding modes are depicted in **Supplementary Fig. 8**.

Supplementary Methods

(*S*)-1-Methyl-1-phenylmethyl-1,2,3,4-tetrahydroisoquinoline-6,7-diol (*S*)-11

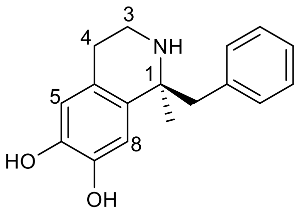

A 5 mL biotransformation for the formation of **11** was set up containing dopamine **1** (75 μmole, 15 mM), phenylacetone **4** (50 μmole, 10 mM), ascorbic acid (5 mM), DMSO (10% v v^{-1}), A79I-Δ29*Tf*NCS lysate (20% v v^{-1}) and HEPES buffer (50 mM, pH 7.5). The reaction was incubated for 6 hours (37 ˚C, 250 rev min^{-1}) before being centrifuged and filtered before extraction. At this stage the conversion yield was measured by determining depletion of dopamine by HPLC analysis. For extraction, the reaction was diluted to 10 mL with water and extracted into ethyl acetate (3 x 20 mL). The organic fractions were combined, washed with brine (3 x 20 mL), dried with magnesium sulfate and the solvent removed *in vacuo*. The residue was resuspended in 20 mL of a 1:1 mixture of dimethylcarbonate (DMC) and 0.1 M HCl. The organic layer was extracted with 0.1 M HCl (2 x 10 mL). The aqueous layers were combined and water was removed *in vacuo* (first by a rotary evaporator and the last few milliliters by freeze-drying) to yield the product as an orange solid (*S*)-**11** (13.3 mg, 91% conversion yield, 87% purified yield, HCl salt, 95% *ee*).

^{1}H NMR (600 MHz; CD_3OD): δ 1.68 (3H, s, Me), 2.94 (2H, t, *J* 6.1 Hz, 4-H_2), 3.27 (1H, d, *J* 14.2 Hz, C*H*HPh), 3.32 (2H, t, *J* 6.1 Hz, 3-H_2), 3.36 (1H, d, *J* 14.2 Hz, CH*H*Ph), 6.60 (1H, s, 5-H), 6.64 (1H, s, 8-H), 7.18 (2H, m, 2 x Ph 2-H), 7.32 (3H, m, 2 x Ph 3-H and Ph 4-H); ^{13}C NMR (150 MHz; CD_3OD): δ 25.8, 26.7, 39.2, 47.2, 61.0, 113.7, 116.2, 123.1, 128.1, 128.8, 129.7, 132.1, 135.5, 145.9, 146.7; IR (film): 3166, 2974, 2792, 1593, 1524 cm^{-1}; HRMS (*m/z*): $[MH]^+$ calcd. for $C_{17}H_{20}NO_2$, 270.1494; found 270.1502; $[\alpha]_D^{20}$ -7.4 (0.14, MeOH).

(*S*)-Methyl-1-(4-methoxy)phenylmethyl-1,2,3,4-tetrahydroisoquinoline-6,7-diol (*S*)-12

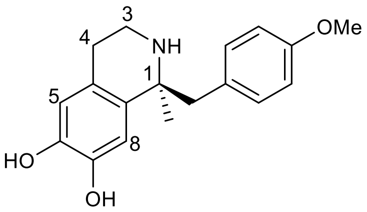

A 5 mL biotransformation for the formation of **12** was set up containing dopamine **1** (75 µmole, 15 mM), 4-methyoxyphenylacetone **5** (50 µmole, 10 mM), ascorbic acid (5 mM), DMSO (10% v v^{-1}), A79I-Δ29*Tf*NCS lysate (20% v v^{-1}) and HEPES buffer (50 mM, pH 7.5). The reaction was incubated for 6 hours (37 ˚C, 250 rev min^{-1}) before being centrifuged and filtered before extraction. At this stage the conversion yield was measured by determining depletion of dopamine by HPLC analysis. For extraction, the reaction was diluted to 10 mL with water and extracted into ethyl acetate (3 x 20 mL). The organic fractions were combined, washed with brine (3 x 20 mL), dried with magnesium sulfate and the solvent removed *in vacuo*. The residue was resuspended in 20 mL of a 1:1 mixture of dimethylcarbonate and 0.1 M HCl. The organic layer was extracted with 0.1 M HCl (2 x 10 mL). The aqueous layers were combined and water was removed *in vacuo* (first by a rotary evaporator and the last few milliliters by freeze-drying) to yield the product as a pale orange solid **12** (11.6 mg, 74% conversion yield, 69% purified yield, HCl salt).

^{1}H NMR (600 MHz; CD_3OD): δ 1.66 (3H, s, Me), 2.92 (2H, t, *J* 6.4 Hz, 4-H_2), 3.20 (1H, d, *J* 14.4 Hz, C*H*HPh), 3.28 (1H, d, *J* 14.2 Hz, CH*H*Ph), 3.31 (2H, t, *J* 6.4 Hz, 3-H_2), 3.77 (3H, s, OMe), 6.59 (1H, s, 5-H), 6.63 (1H, s, 8-H), 6.87 (2H, d, *J* 8.7 Hz, 2 x Ar 3-H), 7.08 (2H, d, *J* 8.7 Hz, 2 x Ar 2-H); ^{13}C NMR (150 MHz; CD_3OD): δ 25.9, 26.7, 39.1, 46.4, 55.7, 61.0, 113.7, 115.1, 116.1, 123.1, 127.2, 128.2, 133.1, 145.9, 146.6, 160.8; IR (film): 3346, 3194, 2973, 2795, 1611, 1512 cm^{-1}; HRMS (*m/z*): $[MH]^+$ calcd. for $C_{18}H_{22}NO_3$, 300.1600; found 300.1605; $[\alpha]_D^{20}$ -35 (0.19, MeOH).

(*rac*)-1-Methyl-1-phenylmethyl-1,2,3,4-tetrahydroisoquinoline-6,7-diol *rac*-11

Step 1

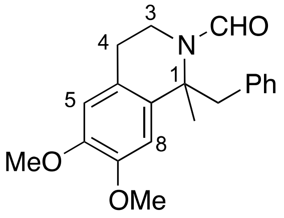

A mixture of 3,4-dimethoxyphenethylamine (1.0 g, 5.5 mmol), phenylacetone **4** (0.62 g, 4.6 mmol) and titanium (IV) isopropoxide (2.0 g, 7.0 mmol) was heated at 80 °C under N_2. After 3 hours, a solution of acetic-formic anhydride [prepared from formic acid (17 mL, 0.45 mol) and acetic anhydride (44 mL, 0.46 mol)] was added at 0 ˚C and the mixture was heated at 70 °C for 2 hours. Trifluoroacetic acid (71 mL, 0.93 mol) was then added and the reaction was stirred for 18 hours at 70 °C. The reaction mixture was diluted with methanol (100 mL) and filtered through silica to remove any TiO_2. The silica was washed with MeOH/$CHCl_3$ (1:1) and the filtrate was concentrated under vacuum to about 50 mL. The solution was diluted with $CHCl_3$ and concentrated to give a brown oil. The oil was purified by silica chromatography (hexane:ethyl acetate, 1:1) to give the crude *N*-formyl tetrahydroisoquinoline product (0.46 g, 31%) as a brown gum which was used directly in the next step.

^{1}H NMR (400 MHz; $CDCl_3$): δ 1.69 (3H, s, CH_3), 2.37 (1H, dt, *J* 15.6 and 4.4 Hz, 4-*H*H), 2.69 (1H, m, 4-H*H*), 3.08 (1H, d, *J* 14.0 Hz, C*H*HPh), 3.13 (1H, m, 3-*H*H), 3.26 (1H, d, *J* 14.0 Hz, CH*H*Ph), 3.83 (3H, s, OMe), 3.87 (3H, s, OMe), 4.23 (1H, dt, *J* 12.8 and 4.4 Hz, 3-H*H*), 6.53 (1H, s, 5-H), 6.76 (1H, s, 8-H), 6.87 (2H, m, 2 x Ph-H), 7.18 (3H, m, 3 x Ph-H), 8.18 (1H, s, CHO).

Step 2

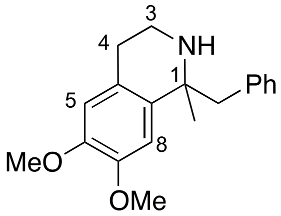

The crude *N*-formyl tetrahydroisoquinoline (0.36 g, 1.1 mmol) in ethanol (20 mL) and 20% NaOH (20 mL) was heated at reflux for 2 days at 100 °C. The reaction mixture was concentrated under vacuum, extracted with ethyl acetate (4 x 15 mL), dried (Na_2SO_4) and filtered. The filtrate was concentrated to give the crude product as a brown oil. The oil was purified by silica chromatography (hexane:ethyl acetate, 1:4) to give 6,7-dimethoxy-1-methyl-1-phenylmethyl-1,2,3,4-tetrahydroisoquinoline as a colourless gum (0.23 g, 70%) (Compound previously reported in: A. I. Meyers, M. A. Gonzalez, V. Struzka, A. Akahane, J. Guiles, J. S. Warmus, Tetrahedron Lett., 1991, 32, 5501-5504).

^{1}H NMR (600 MHz; $CDCl_3$): δ 1.39 (s, 3H, CH_3), 2.54 (1H, dt, *J* 15.6 and 5.4 Hz, 4-*H*H), 2.72 (1H, ddd, *J* 15.6, 7.2 and 5.4 Hz, 4-H*H*), 2.89 (1H, d, *J* 13.2 Hz, C*H*HPh), 3.05 (1H, dt, *J* 12.6 and 5.4 Hz, 3-*H*H), 3.18 (1H, *J* 12.6, 7.2 and 5.4 Hz, 3-H*H*), 3.21 (1H, d, *J* 13.2 Hz, CH*H*Ph), 3.84 (3H, s, OMe), 3.86 (3H, s, OMe), 6.54 (1H, s, 5-H), 6.66 (1H, s, 8-H), 7.07 (2H, m, 2 x Ph-H), 7.25 (3H, m, 3 x Ph-H); ^{13}C NMR (151 MHz; $CDCl_3$): δ 29.7, 30.3, 39.0, 48.0, 55.8, 55.9, 56.2, 109.8, 111.7, 126.5, 127.5, 128.1, 130.7, 134.8, 137.9, 147.1, 147.3.

Step 3

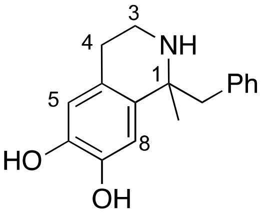

Boron tribromide (1 M in CH_2Cl_2; 4 mL, 4.0 mmol) was added to a solution of 6,7-dimethoxy-1-methyl-1-phenylmethyl-1,2,3,4-tetrahydroisoquinoline (188 mg, 0.632 mmol) in anhydrous dichloromethane (10 mL) under Ar at -78 °C. The reaction was stirred at room temperature for 2 days, then cooled to 0 °C and methanol (5 mL) was added dropwise. After stirring for another 3 hours, the mixture was concentrated under vacuum. The residue was re-suspended in methanol and concentrated under vacuum 3 further times, giving the crude product as brown oil. The oil was dissolved in water (20 mL) and adjusted to pH 7.5 (using 1 M NaOH), then extracted with ethyl acetate (4 x 15 mL), dried (Na_2SO_4), filtered and concentrated in vacuo to give a brown solid. The solid was re-suspended in HCl (1 M) and dimethyl carbonate (DMC) (1:1, 20 mL). The aqueous phase was washed with DMC (2 x 5 mL) and the combined DMC fractions washed with HCl (1 M; 2 x 5 mL). The aqueous phase was combined and evaporated under vacuum to obtain (*rac*)-1-methyl-1-phenylmethyl-1,2,3,4-tetrahydroisoquinoline-6,7-diol hydrochloride salt (*rac*-**11**·HCl) (100 mg, 52%) as a colourless solid. The characterisation data was identical to that for **11** produced by NCS (see above).

3',4'-Dihydro-2'H-spiro[cyclohexane-1,1'-isoquinoline]-6',7'-diol 13

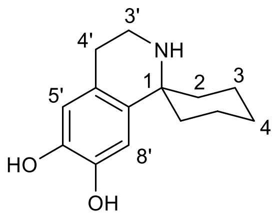

A 5 mL biotransformation for the formation of **13** was set up containing dopamine **1** (75 μmole, 15 mM), cyclohexanone **6** (50 μmole, 10 mM), ascorbic acid (5 mM), DMSO (10% v v^{-1}), A79F-Δ29*Tf*NCS lysate (20% v v^{-1}) and HEPES buffer (50 mM, pH 7.5). The reaction was incubated for 6 hours (37 ˚C, 250 rev min^{-1}) before being centrifuged and filtered before extraction. At this stage the conversion yield was measured by determining depletion of dopamine by HPLC analysis. For extraction, the reaction was diluted to 10 mL with water and extracted into ethyl acetate (3 x 20 mL). The organic fractions were combined, washed with brine (3 x 20 mL), dried with magnesium sulfate and the solvent removed *in vacuo*. The residue was resuspended in 20 mL of a 1:1 mixture of dimethylcarbonate and 0.1 M HCl. The organic layer was extracted with 0.1 M HCl (2 x 10 mL). The aqueous layers were combined and water was removed *in vacuo* (first by a rotary evaporator and the last few milliliters by freeze-drying) to yield the product as a pale brown solid **13** (5.8 mg, 99% conversion yield, 43% purified yield, HCl salt).

^{1}H NMR (600 MHz; CD_3OD): δ 1.40-1.53 (1H, m, 4-H_{ax}), 1.59-1.67 (2H, m, 2 x 3-*H*H), 1.78-1.88 (3H, m, 2 x 3-H*H* and 4-H_{eq}), 1.99-2.07 (4H, m, 2 x 2-H_2), 2.97 (2H, t, *J* 6.3 Hz, 4'-H_2), 3.42 (2H, t, *J* 6.3 Hz, 3'-H_2), 6.57 (1H, s, 5'-H), 6.75 (1H, s, 8'-H); ^{13}C NMR (150 MHz; CD_3OD): δ 21.6, 25.3, 26.2, 36.8, 38.6, 61.0, 113.3, 116.1, 123.0, 129.8, 146.0, 146.5; IR (film): 3345, 3187, 2933, 2858, 1593, 1524 cm^{-1}; HRMS (*m/z*): $[MH]^+$ calcd. for $C_{14}H_{20}NO_2$, 234.1494; found 234.1498.

4-Methyl-3',4'-dihydro-2'H-spiro[cyclohexane-1,1'-isoquinoline]-6',7'-diol 14

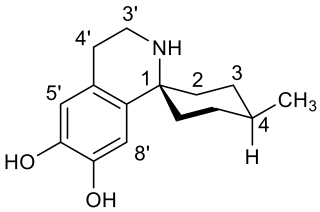

A 5 mL biotransformation for the formation of **14** was set up containing dopamine **1** (75 µmole, 15 mM), 4-methycyclohexanone **7** (50 µmole, 10 mM), ascorbic acid (5 mM), DMSO (10% v v^{-1}), A79F-Δ29*Tf*NCS lysate (20% v v^{-1}) and HEPES buffer (50 mM, pH 7.5). The reaction was incubated for 6 hours (37 ˚C, 250 rev min^{-1}) before being centrifuged and filtered before extraction. At this stage the conversion yield was measured by determining depletion of dopamine by HPLC analysis. For extraction, the reaction was diluted to 10 mL with water and extracted into ethyl acetate (3 x 20 mL). The organic fractions were combined, washed with brine (3 x 20 mL), dried with magnesium sulfate and the solvent removed *in vacuo*. The residue was resuspended in 20 mL of a 1:1 mixture of dichloromethane and 0.1 M HCl. The organic layer was extracted with 0.1 M HCl (2 x 10 mL). The aqueous layers were combined and water was removed *in vacuo* (first by a rotary evaporator and the last few milliliters by freeze-drying) to yield the product as a pale grey solid **14** (7.3 mg, 99% conversion yield, 51% purified yield, HCl salt).

^{1}H NMR (600 MHz; CD_3OD): δ 1.04 (3H, d, *J* 6.5 Hz, CH_3), 1.26-1.35 (2H, m, 2 x 3-H_{ax}), 1.61-1.69 (1H, m, 4-H_{ax}), 1.78 (2H, app. d, *J* 14.3 Hz, 2 x 3-H_{eq}), 2.05-2.10 (4H, m, 2 x 2-H_2), 2.96 (2H, t, *J* 6.3 Hz, 4'-H_2), 3.41 (2H, t, *J* 6.3 Hz, 3'-H_2), 6.56 (1H, s, 5'-H), 6.73 (1H, s, 8'-H); ^{13}C NMR (150 MHz; CD_3OD): δ 22.2 (Me), 26.2, 30.1, 32.4, 36.9, 38.7, 60.6, 113.2), 116.1, 123.1, 129.5, 146.1, 146.5; IR (film): 3345, 3223, 2950, 2868, 2794, 1613, 1525 cm^{-1}; HRMS (*m/z*): $[MH]^+$ calcd. for $C_{15}H_{22}NO_2$, 248.1651; found 248.1650.

4-(*tert*-Butyl)-3',4'-dihydro-2'H-spiro[cyclohexane-1,1'-isoquinoline]-6',7'-diol 15

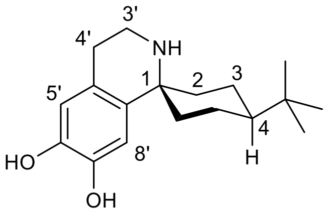

A 5 mL biotransformation for the formation of **15** was set up containing dopamine **1** (75 μmole, 15 mM), 4-*tert*-butyl-cyclohexanone **8** (50 μmole, 10 mM), ascorbic acid (5 mM), DMSO (10% v v^{-1}), A79F-Δ29*Tf*NCS lysate (20% v v^{-1}) and HEPES buffer (50 mM, pH 7.5). The reaction was incubated for 6 hours (37 ˚C, 250 rev min^{-1}) before being centrifuged and filtered before extraction. At this stage the conversion yield was measured by determining depletion of dopamine by HPLC analysis. For extraction, the reaction was diluted to 10 mL with water and extracted into ethyl acetate (3 x 20 mL). The organic fractions were combined, washed with brine (3 x 20 mL), dried with magnesium sulfate and the solvent removed *in vacuo*. The residue was resuspended in 20 mL of a 1:1 mixture of dichloromethane and 0.1 M HCl. The organic layer was extracted with 0.1 M HCl (2 x 10 mL). The aqueous layers were combined and water was removed *in vacuo* (first by a rotary evaporator and the last few milliliters by freeze-drying) to yield the product as a colorless solid **15** (5.4 mg, 66% conversion yield, 33% purified yield, HCl salt).

^{1}H NMR (600 MHz; CD_3OD): δ 0.96 (9H, s, $C(CH_3)_3$), 1.28-1.40 (3H, m, 4-H_{ax}, 2 x 3-H_{ax}), 1.87 (2H, app. d, *J* 13.0 Hz, 2 x 3-H_{eq}), 2.05 (2H, dd, *J* 15.0 and 13.4 Hz, 2 x 2-H_{ax}), 2.13 (2H, d, *J* 15.0 Hz, 2 x 2-H_{eq}), 2.96 (2H, t, *J* 6.3 Hz, 4'-H_2), 3.42 (2H, t, 6.3 Hz, 3'-H_2), 6.57 (1H, s, 5'-H), 6.73 (1H, s, 8'-H); ^{13}C NMR (150 MHz; CD_3OD): δ 23.2, 26.3, 27.9, 33.3, 37.7, 38.9), 48.4, 60.8, 113.3, 116.0, 123.3, 129.4, 146.0, 146.5; IR (film): 3330, 3227, 2952, 2868, 1612, 1525 cm^{-1};HRMS (*m/z*): $[MH]^+$ calcd. for $C_{18}H_{28}NO_2$, 290.2120; found 290.2120.

4-Phenyl-3',4'-dihydro-2'H-spiro[cyclohexane-1,1'-isoquinoline]-6',7'-diol 16

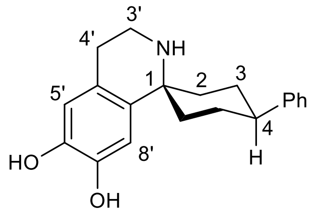

A 5 mL biotransformation for the formation of **16** was set up containing dopamine **1** (75 µmole, 15 mM), 4-phenyl-cyclohexanone **9** (50 µmole, 10 mM), ascorbic acid (5 mM), DMSO (10% v v^{-1}), A79F-Δ29*Tf*NCS lysate (20% v v^{-1}) and HEPES buffer (50 mM, pH 7.5). The reaction was incubated for 6 hours (37 ˚C, 250 rev min^{-1}) before being centrifuged and filtered before extraction. At this stage the conversion yield was measured by determining depletion of dopamine by HPLC analysis. For extraction, the reaction was diluted to 10 mL with water and extracted into ethyl acetate (3 x 20 mL). The organic fractions were combined, washed with brine (3 x 20 mL), dried with magnesium sulfate and the solvent removed *in vacuo*. The residue was resuspended in 20 mL of a 1:1 mixture of dichloromethane and 0.1 M HCl. The organic layer was extracted with 0.1 M HCl (2 x 10 mL). The aqueous layers were combined and water was removed *in vacuo* (first by a rotary evaporator and the last few milliliters by freeze-drying) to yield the product as a white solid **16** (10.0 mg, 82% conversion yield, 58% purified yield, HCl salt).

^{1}H NMR (600 MHz; CD_3OD): δ 1.80-1.88 (2H, m, 2 x 3-H_{ax}), 1.97 (2H, d, *J* 14.6 Hz, 2 x 3-H_{eq}), 2.21 (2H, d, *J* 14.9 Hz, 2 x 2-H_{eq}), 2.23-2.30 (2H, m, 2 x 2-H_{ax}), 2.83 (1H, tt, *J* 12.3 and 3.4 Hz, 4-H_{ax}), 3.00 (2H, t, *J* 6.3 Hz, 4'-H_2), 3.49 (2H, t, *J* 6.3 Hz, 3'-H_2), 6.59 (1H, s, 5'-H), 6.83 (1H, s, 8'-H), 7.20 (1H, tt, *J* 7.3 and 1.2 Hz, Ph 4-H), 7.31 (2H, m, 2 x Ph 3-H), 7.36 (2H, app. d, *J* 7.9 Hz, 2 x Ph 2-H); ^{13}C NMR (150 MHz; CD_3OD): δ 26.3, 29.5, 37.3, 38.8, 44.0, 60.6, 113.3, 116.1, 123.2, 127.5, 127.9, 129.3, 129.6, 146.2, 146.6, 146.9; IR (film): 3528, 3419, 3152, 3063, 2936, 2762, 1594, 1518 cm^{-1};HRMS (*m/z*): $[MH]^+$ calcd. for $C_{20}H_{24}NO_2$, 310.1807; found 310.1808.

(1*R*,3*R*)-3-(*M*ethyl)-3',4'-dihydro-2'H-spiro[cyclohexane-1,1'-isoquinoline]-6',7'-diol 17

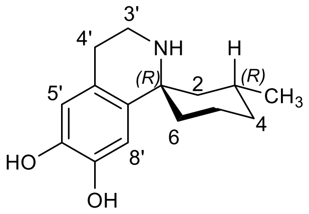

A 5 mL biotransformation for the formation of **17** was set up containing dopamine **1** (100 μmole, 20 mM), (3*R*)-methylcyclohexanone (*R*)-**10** (50 μmole, 10 mM), ascorbic acid (5 mM), DMSO (10% v v^{-1}), A79F-Δ29*Tf*NCS lysate (50% v v^{-1}) and HEPES buffer (50 mM, pH 7.5). The reaction was incubated for 16 hours (37 ˚C, 250 rev min^{-1}) before being centrifuged and filtered before extraction. At this stage the conversion yield was measured by determining depletion of dopamine by HPLC analysis. For extraction, the reaction was diluted to 10 mL with water and extracted into ethyl acetate (3 x 20 mL). The organic fractions were combined, washed with brine (3 x 20 mL), dried with magnesium sulfate and the solvent removed *in vacuo*. The residue was resuspended in 20 mL of a 1:1 mixture of dimethylcarbonate and 0.1 M HCl. The organic layer was extracted with 0.1 M HCl (2 x 10 mL). The aqueous layers were combined and water was removed *in vacuo* (first by a rotary evaporator and the last few milliliters by freeze-drying) to yield the product as a green solid **17** (3.9 mg, 98% conversion yield, 27% purified yield, HCl salt).

^{1}H NMR (600 MHz; CD_3OD): δ 1.00 (3H, d, *J* 6.3 Hz, CH_3), 1.14 (1H, dq, *J* 12.6 and 4.2 Hz, 4-H_{ax}), 1.61-1.69 (2H, m, 2H, 2-H_{ax} and 5-H_{ax}), 1.75 (1H, br m, 3-H_{ax}), 1.82-1.88 (2H, m, 4-H_{eq} and 5-H_{eq}), 1.93 (1H, td, *J* 14.4 and 4.3 Hz, 6-H_{ax}), 2.00-2.08 (2H, m, 2-H_{eq} and 6-H_{eq}), 2.96 (2H, td, *J* 6.3 and 2.3 Hz, 4'-H_2), 3.42 (2H, m, *J* 6.3 and 4.3 Hz, 3'-H_2), 6.57 (1H, s, 5'-H), 6.73 (1H, s, 8'-H); ^{13}C NMR (150 MHz; CD_3OD): δ 21.6, 22.6, 26.2, 28.3, 34.1, 36.3, 38.7, 45.3, 61.7, 113.2, 116.0, 123.0, 129.6, 146.1, 146.5; IR (film): 3346, 2952, 2799, 1592, 1525 cm^{-1}; HRMS (*m/z*): $[MH]^+$ calcd. for $C_{15}H_{22}NO_2$, 248.1651; found 248.1653.
